# Targeting *Streptococcus mutans* Antigen I/II To Inhibit Dual-Species Biofilms with *Candida albicans*

**DOI:** 10.1021/acsmedchemlett.6c00275

**Published:** 2026-07-27

**Authors:** B. Owen Garrett, Parvathy Babu, Bhavitavya Nijampatnam, Abhishek Govindan, Norbert Schormann, Manisha Patel, Gregory Harber, Suzanne M. Michalek, Hui Wu, Champion Deivanayagam, Sadanandan E. Velu

**Affiliations:** †Department of Chemistry, ‡Department of Biochemistry and Molecular Genetics, §Department of Microbiology, and ∥Global Center for Craniofacial Oral and Dental Disorders, University of Alabama at Birmingham, Birmingham, Alabama 35294, United States; ⊥ Center for Clinical and Translational Sciences, University of Alabama at Birmingham, Birmingham, Alabama 35294, United States; # Department of Biomaterial and Biomedical Science, Oregon Health and Science University, Portland, Oregon 97239, United States

**Keywords:** *S. mutans*, Antigen I/II, *C*. *albicans*, dental caries, biofilm, inhibitor

## Abstract

We employed structure-based drug design to target the V-region of *Streptococcus mutans* antigen I/II (V^AgI/II^) and identified inhibitors of *S. mutans* (*SM*) – *C. albicans* (*CA*) dual-species biofilms. The lead compound, **SN195**, inhibited *SM* single-species biofilms and *SM–CA* dual-species biofilms at IC_50_ values of 49.9 and 23.6 μM, respectively, without affecting the planktonic growth of either microbe. **SN195** at 100 μM did not affect the growth of the tested oral commensals. The X-ray cocrystal structure of **SN195** bound to V^AgI/II^ revealed crucial interactions in the vicinity of the conserved Ca^2+^ ion in the binding pocket. Structure activity relationship studies resulted in the development of more potent dual-species biofilm inhibitors, **1b** and **1j**, with IC_50_ values of 12.9 μM and 0.41 μM, respectively. In a dual-species gnotobiotic rat caries model coinfected with *SM* and *CA*, treatment with **SN195**, **1b**, or **1j** significantly reduced buccal and sulcal caries scores compared to infected and untreated control rats.

## Graphical abstract

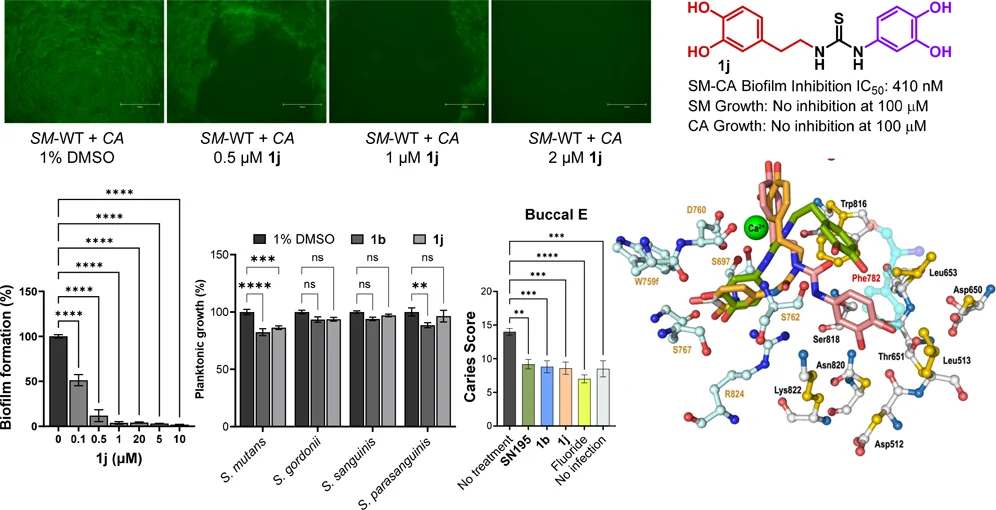

Dental caries, a ubiquitous disease affecting the vast majority of human populations worldwide,^1^ is a multifactorial condition in which microorganisms, including bacteria and fungi, play significant roles in disease initiation and progression. *Streptococcus mutans* (*SM*), consistently observed in carious lesions, is considered one of the primary etiological agents of this disease.^2^ Interkingdom interactions between *SM* and the fungal pathogen *Candida albicans* (*CA*) increase both the severity and incidence rate of this disease.^3–9^

Although the mechanism of the synergistic interaction between *SM* and *CA* is not completely understood, recent literature suggests that multiple surface- and/or secreted proteins of *SM* are involved in this interaction. Among the secreted factors, *SM*’s glucosyl transferases (Gtfs) bind to the cell surface of *CA* and enhance dual-species biofilm formation in a sucrose-dependent manner.^10^ Gtf-mediated sucrose-dependent matrix assembly provides a key mechanism for cross-kingdom biofilm development and enhanced cariogenic potential. In addition to Gtfs, the interaction of *SM*’s surface adhesin antigen I/II (*SM*-AgI/II), a member of the AgI/II family of proteins in many oral streptococci, plays a critical role in incorporating *CA* into dual-species biofilms^9^ via sucrose-independent mechanisms.^11^ AgI/II contains two distinct regions: a variable V-region (V^AgI/II^) at the head of the stalk and a C-terminal region (C_123_^AgI/II^).^12,13^ The V-region features a prominent central groove, which hosts a conserved Ca^2+^ ion binding site. Both the V-region and C-terminal regions of AgI/II bind to salivary agglutinin glycoprotein (Gp340), and this redundant adherence is mediated through scavenger receptor cysteine-rich (SRCR) domains of Gp340.^14^ Other AgI/II family members such as *SM*’s GbpC and *S. gordonii* SspB have also been implicated in bacterial adherence to Gp340.^14,15^ Recent reports suggest that *CA* surface adhesins Als3 and potentially Als5 may be involved in the interaction with bacterial cell-surface proteins, contributing to mixed-species biofilm formation.^16^ However, interaction sites of *CA* with *SM* AgI/II remain less clearly defined.

Our previous studies have shown that deletion of AgI/II in *SM* (*SM*-ΔAgI/II) reduced the ability of *SM* to incorporate *CA* into the dual-species biofilm.^9^ Together, these findings suggest that *SM*-AgI/II alongside Gtfs plays a central role in orchestrating cross-kingdom interactions between *SM* and *CA*.^17^ Targeting Gtf-mediated dual-species interactions has already shown promise in attenuating cariogenic biofilms and reducing caries in preclinical models.^18–21^ In contrast, it remains unexplored whether targeting AgI/II-mediated interactions will be equally effective. In this context, we investigated three V-region-containing surface adhesins, namely, V^AgI/II^, V^GbpC^, and V^SspB^, that display similar protein folding with subtle differences in certain regions.^22^ Site-directed mutagenesis studies resulted in the identification of specific residues in each V-region that played a significant role in adherence to SRCR domains of Gp340.^22^

These findings suggested that the SRCR adhesion site within the V regions could facilitate other interactions, such as with *CA*. In this study, we confirm that the V region interacts with *CA* and present the results of our structure-based drug design, wherein we have identified inhibitors that target biofilm formation aided by the V region and specifically target the pathogenic species and spare the growth of commensal bacteria in the oral cavity. This work is significant for being one of the first to demonstrate the efficacy of novel therapeutics for dental caries that target AgI/II family adhesins.

Over the last two decades, we have investigated the structure–function correlations of adhesive surface proteins on *SM* and its interactions to identify methods for inhibiting bacterial adhesion to tooth surfaces and consequently preventing biofilm development. Toward this, we have resolved the crystal structures of various surface proteins of *SM* and identified specific residues involved in adhesion to Gp340.^12,13,15,22–24^ We recently reported the X-ray crystal structures of the V-region of several AgI/II family proteins including V^AgI/II^ (6TZL**)**, V^SspB^ (6Q2L), and V^SspB^ + TEV^pep^ (6Q2K) complexed with a 12-mer peptide (^778^ENLYFQGAAALE^789^, TEV^pep^_,_ Figure 1A).^22^ By superposing V^AgI/II^, V^GbpC^, and the V^SspB^ + TEV^pep^ cocrystal structure and through alanine mutational analysis, distinct residues within V^AgI/II^, V^GbpC^, and V^SspB^ were identified to influence binding. In V^AgI/II^, four residues, Phe656, Ser697, Asp760, and Asn820, affected binding (Figure 1A). Because the TEV^pep^ binding area within V^AgI/II^ is relatively large for small molecule targeting, we identified three smaller subpockets for inhibitor design: (1) the ‘tyrosine pocket’ around Tyr781, (2) the ‘phenylalanine pocket’ near Phe782, and (3) the ‘basal opening’ near the C-terminal end of the peptide (Figure 1B,C). Among these, the ‘phenylalanine pocket’ was selected for *in silico* screening as it is close to residue Asn820 (Figure 1A), which has the most pronounced effect on adhesion to Gp340.^22^

**Figure 1:**
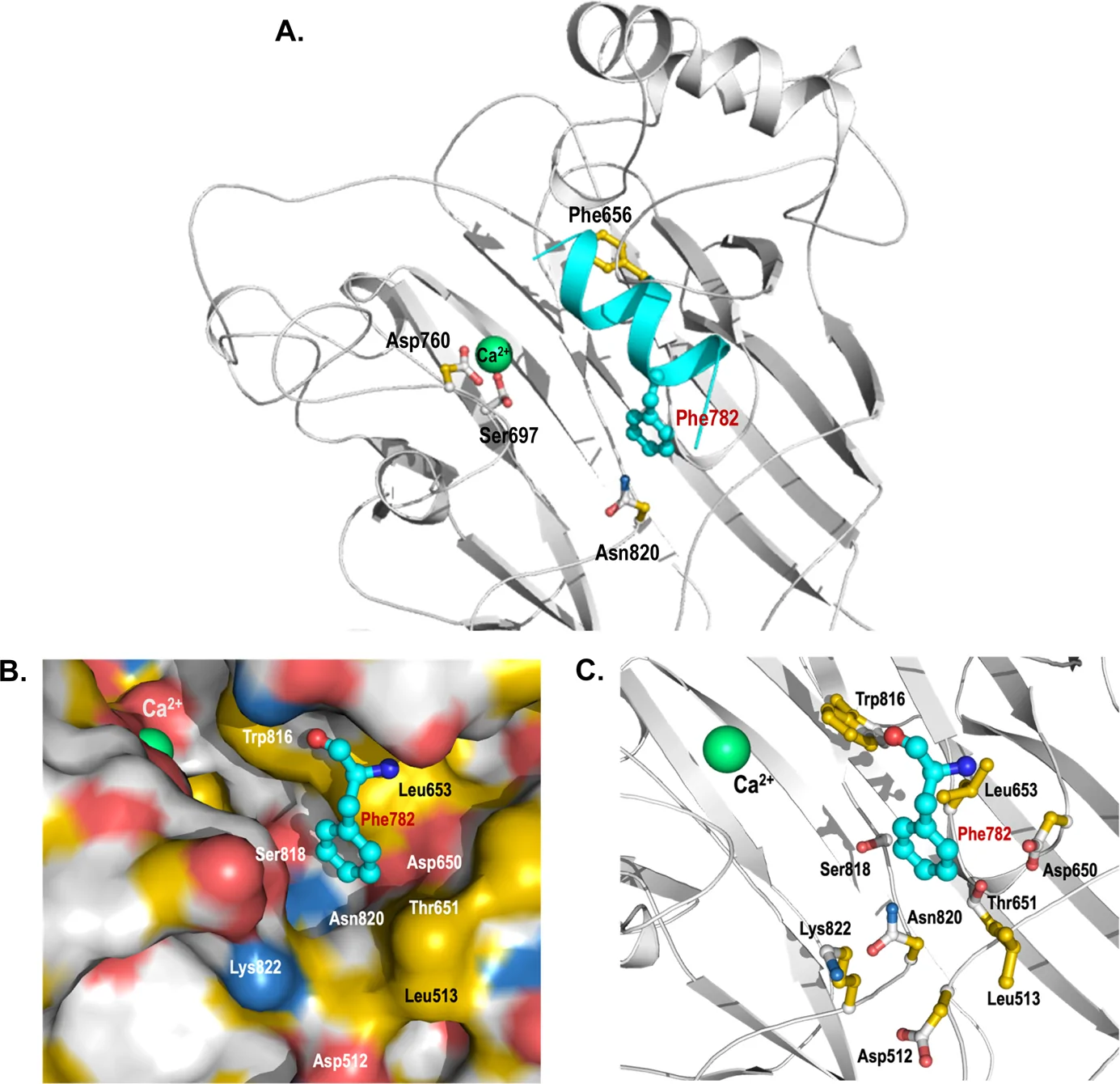
(A) Co-crystal structure of V^AgI/II^ superposed with the TEV^pep^. The residue phenylalanine 782 of TEV^pep^ (cyan, ball and stick) is highlighted to describe the pocket. The residues that displayed diminished binding to Gp340, namely, Phe656, Ser697, Asp760, and Asn820, are shown in the cartoon figure. (B) Zoomed in surface model of the phenylalanine pocket identified in the TEV^pep^ binding region of V^AgI/II^ with the peptide residue Phe782 displayed in cyan. (C) Zoomed in cartoon representation of phenylalanine pocket with the peptide residue Phe782 displayed in cyan and the key binding residues displayed as ball and stick.

Because *SM*-AgI/II is a sucrose-independent virulence factor of *SM*, we investigated dual-species biofilm formation in the absence of sucrose, which differed from our earlier investigation, where 1% sucrose was used in biofilm studies.^9^ Using green fluorescent protein (GFP) tagged *SM* wild type UA159 (*SM*-WT) and AgI/II mutant (*SM*-ΔAgI/II), we first measured the baseline fluorescence for *SM*, *SM*-ΔAgI/II, and *CA*, where each individually displayed very low signals (Figure 2A). In dual-species biofilms with *SM*-WT and *CA*, there was a considerable increase in fluorescence (Figure 2A). When using *SM*-ΔAgI/II and *CA*, fluorescence intensity was reduced by approximately 50%. Single-species biofilms of *SM*-WT and *SM*-ΔAgI/II showed minimal biomass, whereas *CA* alone produced substantial biofilm (Figure 2B). In dual-species biofilms, *SM*-ΔAgI/II–*CA* displayed 40% less biofilm biomass, compared to the *SM*-WT–*CA* biofilm (Figure 2B). *SM*-ΔAgI/II showed a comparable, albeit minor, decrease in colony forming units (CFUs) compared to *SM*-WT (Figure 2C). Growth curves monitored for *SM*-WT and *SM*-ΔAgI/II were identical, indicating that the absence of AgI/II does not affect *SM’*s planktonic growth (Figure 2D). Taken together, these results suggest that AgI/II enhances the interaction between *SM* and *CA*, promoting biofilm formation, and deletion of AgI/II (*SM*-ΔAgI/II) reduced dual-species biofilms. Importantly, both *SM* and *SM*-ΔAgI/II exhibited comparable planktonic growth, suggesting that the absence of AgI/II does not impair the organism’s growth (Figure 2D). These results confirm AgI/II’s involvement in the sucrose-independent interkingdom interaction between *SM* and *CA*.^9^

**Figure 2:**
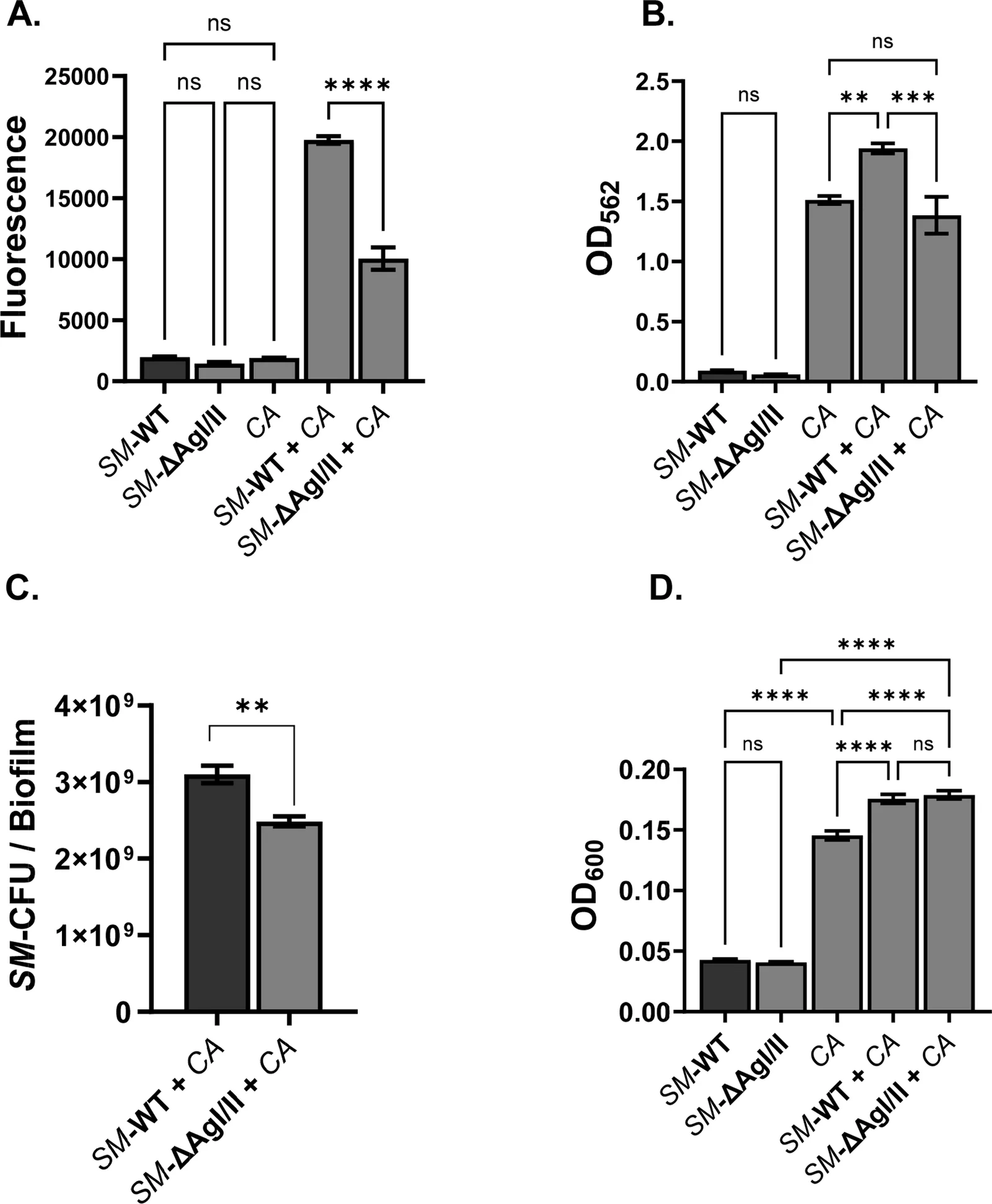
Dual-species biofilm formation and growth of *S. mutans* (*SM*-WT) and *C. albicans* (*CA*). (A) Fluorescence of *SM*-WT, *SM*-ΔAgI/II, *CA*, *SM*-WT + *CA*, and *SM*-ΔAgI/II + *CA* biofilms measured at excitation 485 nm and emission 528 nm after 16 h of growth. (B) Biofilm biomass measured at OD_562_ using crystal violet staining for *SM*-WT, *SM*-ΔAgI/II, *CA*, *SM*-WT + *CA*, and *SM*-ΔAgI/II + *CA*. (C) *S. mutans* CFUs in dual-species biofilms for *SM*-WT + *CA*, and *SM*-ΔAgI/II + *CA*. (D) Planktonic growth of *SM*-WT, *SM*-ΔAgI/II, *CA*, *SM*-WT + *CA*, and *SM*-ΔAgI/II + *CA* measured at OD_600_.

*In silico* screening of a 200,000 ‘drug-like’ small molecule library against the ‘phenylalanine pocket’ was carried out using the FlexX/LeadIT (BIOSolveIT gmbh). The V^AgI/II^ binding site included residues Asp512, Leu513, Asp650, Thr651, Leu653, Gly654, Trp816, Ser818, Asn820, and Lys822 and cofactors within 6.5 Å from the ‘phenylalanine pocket’ (Figure 1B). The 77 top-scoring compounds were examined based on their binding energy, binding site interactions, drug-like properties, and synthetic feasibility, and then tested in the *SM* single-species biofilm assay.^25,26^ The most active compound was a urea derivative, **SN195** (Figure 3A), which inhibited *SM* single-species biofilms with an IC_50_ value of 49.9 μM (Figure 3B) and caused only a modest inhibition of *SM’s* growth (15%) at 100 μM, at double the concentration of its biofilm inhibition IC_50_, indicating a preference for biofilm inhibition over growth reduction (Figure 3B,C). Conversely, **SN195** did not inhibit growth of the commensal species *S. gordonii*, *S. sanguinis*, and *S. parasanguinis* at 100 μM (Figure 3C).

**Figure 3:**
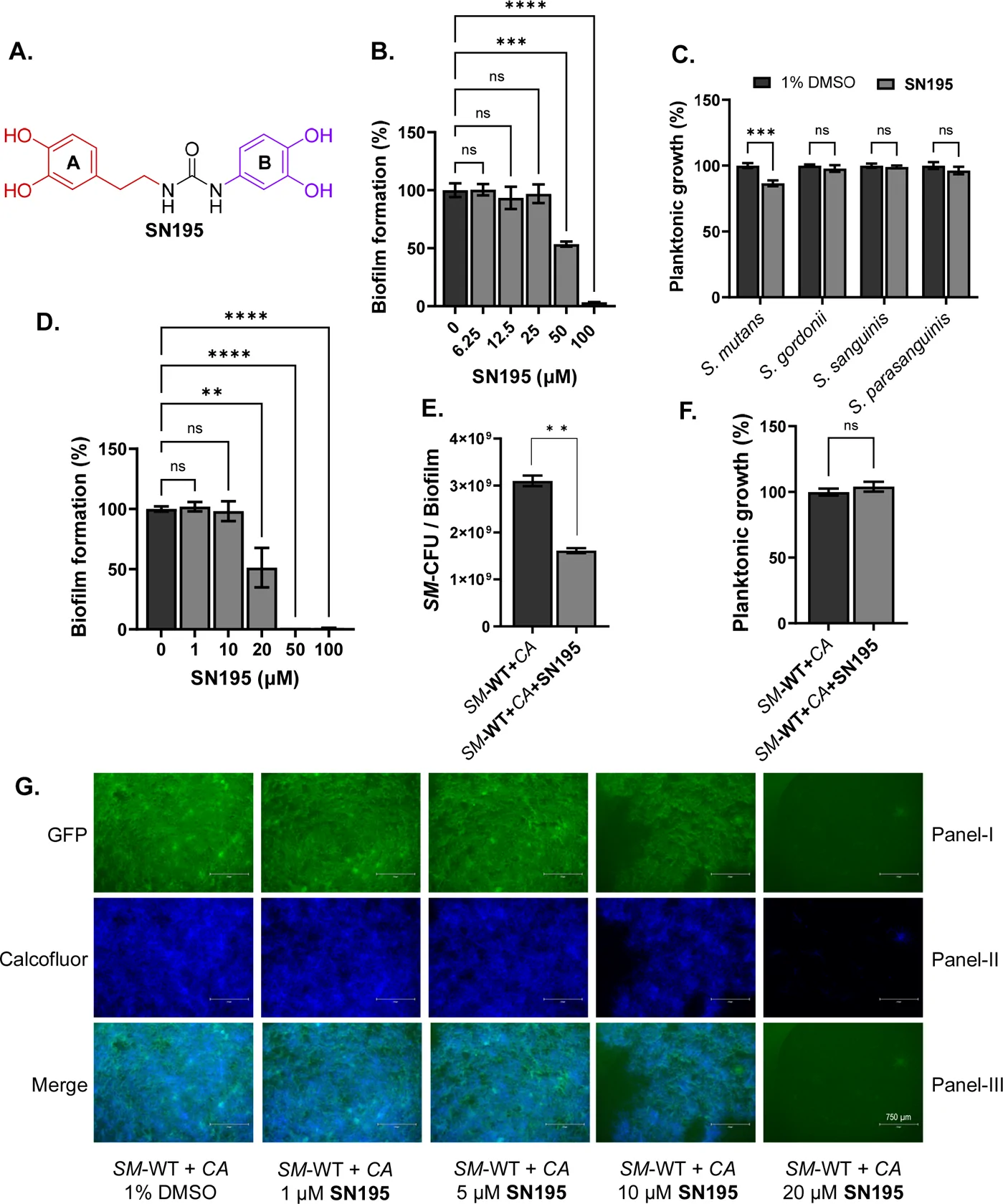
(A) Chemical structure of **SN195**. (B) *S. mutans* UA159 was co-incubated with **SN195** at the indicated concentrations, and single-species biofilm formation was measured at OD_562_ using the crystal violet protocol. (C) *S. mutans* UA159, *S. gordonii* DL1, *S. sanguinis* SK36, and *S. parasanguinis* FW213 were co-incubated with **SN195** at 100 μM, and their growth was measured at OD_600_. (D) Dose-dependent inhibition of dual-species biofilms of *SM*-WT with *CA* at various concentrations of **SN195**. Biofilm biomass was determined after 16 h using crystal violet staining at 562 nm, where *SM*-WT + *CA* was normalized to 100%. (E) CFUs in dual-species biofilms. CFUs were determined at 16 h of growth with *SM*-WT + *CA*, vehicle control of 1% DMSO, and 100 μM **SN195**. (F) Planktonic growth of dual species with *SM*-WT + *CA* with 1% DMSO as vehicle and with 100 μM **SN195** after 16 h of incubation measured at OD_600_. (G) Panel I: Representative fluorescent microscopy images of *SM*-WT + *CA* dual species biofilms treated with various concentrations of **SN195** using GFP as fluorophore. Panel II: Representative images of *SM*-WT + *CA* dual species biofilms with various concentrations of **SN195** generated using Calcofluor White as fluorophore. Panel III: Merged images. The scale bar measures 750 μm. All biofilm and growth experiments were performed in triplicate, and statistical significance was determined using one-way ANOVA, two-way ANOVA, or Student’s *t* test. ns = *p* > 0.05, ** = *p* < 0.01, *** = *p* < 0.001, **** = *p* < 0.0001.

Using established assays,^27^ we examined the role of AgI/II in *SM*–*CA* dual-species biofilms without sucrose. **SN195** inhibited dual-species biofilms at an IC_50_ of 23.6 μM (Figure 3D). Both fluorescence microscopy and biofilm biomass amounts were consistent, showing reduced *SM* incorporation at 10 μM and almost complete loss of incorporation at 20 μM of **SN195** (Figure 3D,G). CFU counts further supported these observations, where ∼50% fewer *SM* colonies were recovered from dual-species biofilms treated with 100 μM of **SN195** compared to controls (Figure 3E). More importantly, **SN195** did not affect the planktonic growth of either microorganism (Figure 3F). Together these data indicate that **SN195** robustly suppressed dual-species biofilm formation and reduced *SM*’s colony numbers while not affecting the planktonic growth of *SM* and *CA*. **SN195** appears to be a specific inhibitor, given its preference to disrupt dysbiotic biofilm formation over planktonic growth.

The **V** ^**AgI/II**^ **+ SN195** complex crystal structure was solved to confirm target engagement of **SN195** and define its binding site interactions (Figure 4, Table S1 in Supporting Information). A notable feature of V-region structures is the presence of a highly conserved Ca^2+^ ion binding site (Figure 1A,B) that is located at the other end of the phenylalanine pocket and basal opening.^15,22,24,28^ Among the four molecules in the asymmetric unit, two distinct conformations of **SN195** were observed (Figure 4A,B). Three molecules (in ABC subunits) adopted conformation I (Figure 4A), while one molecule (in the D subunit) adopted a different conformation, II (Figure 4B). In all four cases, the inhibitor binds in the vicinity of the phenylalanine pocket adjacent to the highly conserved Ca^2+^-binding site. In conformation I, the binding poses overlapped in their interaction with Trp759 and included a H-bond of the urea oxygen atom with Arg824 (Figure 4A).

**Figure 4:**
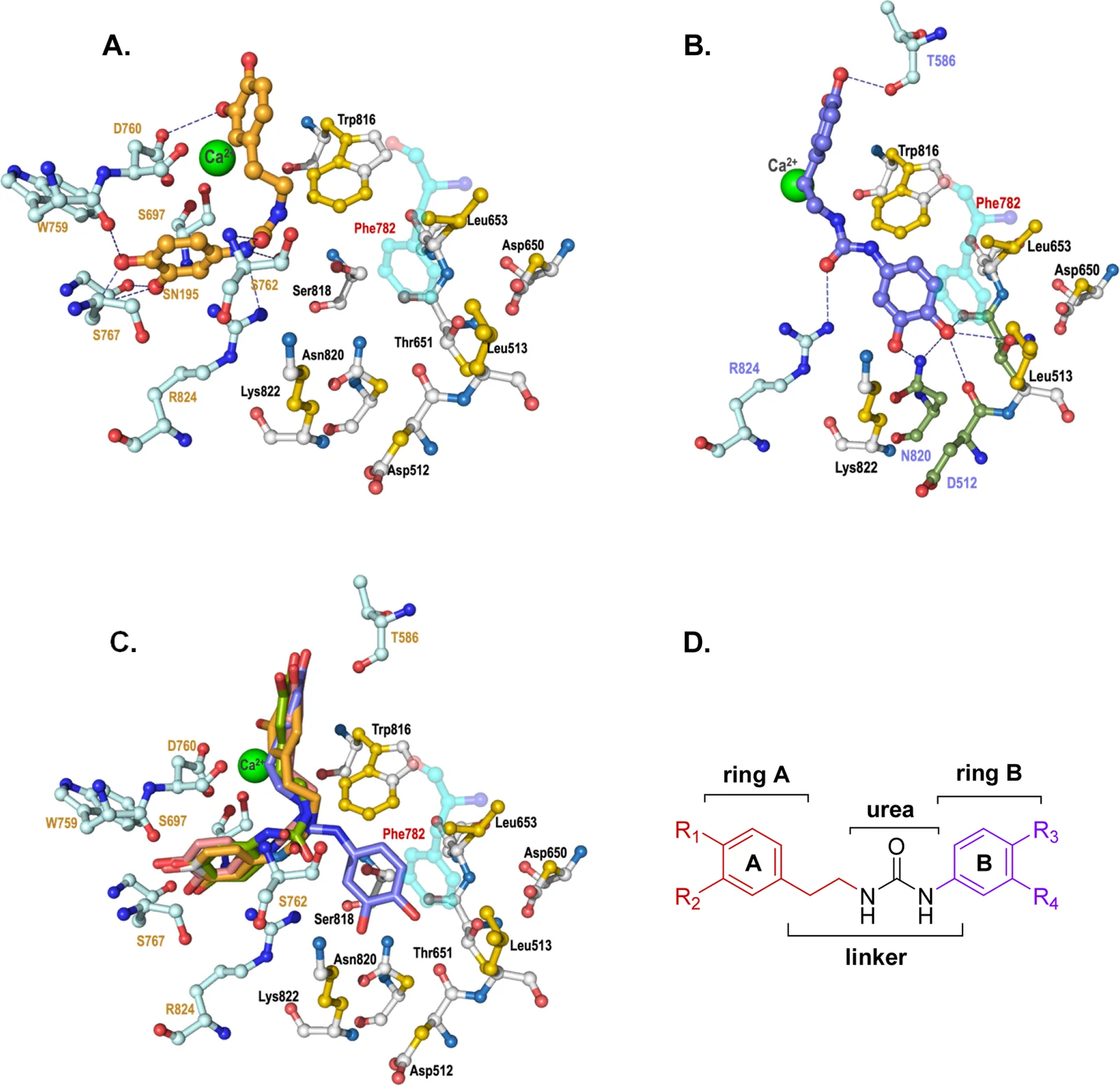
**V** ^**AgI/II**^ + **SN195** cocrystal structure analysis. (A) X-ray crystal structure of the inhibitor **SN195** in complex with **V** ^**AgI/II**^. **SN195** in conformation I (gold) binds in the vicinity of the phenylalanine pocket adjacent to the Ca^2+^ binding site. (B) **SN195** in conformation II (violet). Key hydrogen bonding interactions of **SN195** with the binding site residues are depicted as dashed lines. (C) Superposition of all four **SN195** molecules within the crystal structure showing that three **SN195** adopt a very similar conformation, whereas one molecule adopted an extended conformation comparable to the pose that we generated from docking studies. The amino acids that are shown in three letter codes were chosen for modeling/studies, while those with the single letter codes are previously unanticipated interactions. (D) Regions in **SN195** chosen for structural modifications in structure–activity relationship studies.

In both conformations, the *m*-OH on ring A coordinated with the Ca^2+^ ion. Conformation I engaged the main-chain carbonyl oxygens of Ser697 and Asp760 while conformation II interacted with Thr586 (Figure 4A,B). Ring A also participated in π–π stacking with Trp816. In conformation I, ring B extended deep into a pocket adjacent to the phenylalanine pocket forming hydrogen bonds with Ser767 and Ser697. In conformation II, ring B flipped outward and formed hydrogen bonds with Asp512, Asn820, and Thr651. The presence of two distinct conformations in the crystal structure provided insight into how **SN195** forms multiple stable interactions with V^AgI/II.^

Based on the binding interactions observed for both conformations and their contacts with nearby residues and the Ca^2+^ ion (Figure 4C), four regions in the structure of **SN195** were rationally modified to modulate and fine-tune ligand–protein interactions (Figure 4D): (a) ring A substitutions to enhance interactions with the Ca^2+^ ion and Asp760 and Trp816; (b) ring B substitutions to strengthen its interactions with Ser697, Ser767, and Arg824; (c) linker between rings A and B for optimal linker distance; and (d) replacement of urea with functional groups such as amide or thiourea to improve interactions with Ser762 and Arg824 (Figure 4C). Using this structure-based rationale, 294 new analogs were designed and modeled into the **SN195** binding site of V^AgI/II^ using SeeSAR 13.0.1,^29^ and their binding energies were predicted using the HYDE scoring function.^30,31^ Among these, ten high-scoring analogs (**1a**–**j**, Table 1) were chosen and chemically synthesized. Detailed procedures for the synthesis and characterization of **SN195** and analogs are included in the Supporting Information for this manuscript.

**Table 1:**
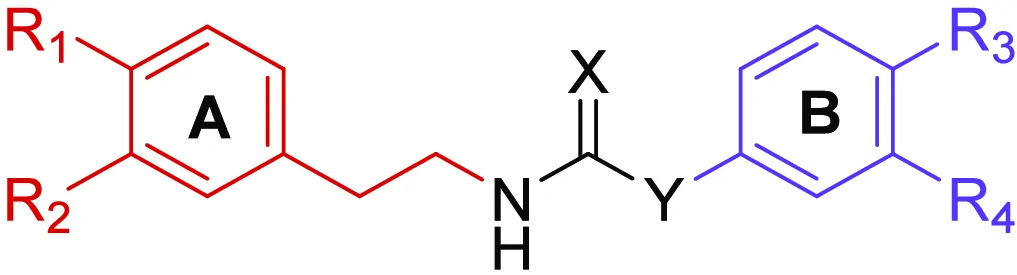
Analogs of **SN195** and Their Predicted Binding Energies, Dual-Species Growth, and Biofilm Inhibition Activities

compd no.	R_1_	R_2_	R_3_	R_4_	X	Y	AgI/II binding energy^a^ (kJ/mol)	Growth inhibition at 100 μM^b^	Abrogation at 100 μM^c^	Dual-species biofilm IC_50_ ^c^ (μM)
**SN195**	OH	OH	OH	OH	O	-NH-	–26.55	NI	Complete	23.6 ± 2.6
**1a**	OH	OH	OH	H	O	-NH-	–21.29	NI	Partial	>100
**1b**	OH	H	OH	OH	O	-NH-	–21.91	NI	Complete	12.9 ± 0.7
**1c**	OH	OH	NO_2_	H	O	-NH-	–28.81	NI	Complete	55.3 ± 6.3
**1d**	OH	OH	COOMe	H	O	-NH-	–23.95	NI	Complete	32.9 ± 14.7
**1e**	NH_2_	H	OH	OH	O	-NH-	–19.13	NI	Complete	35.7 ± 3.1
**1f**	OH	OH	NH_2_	H	O	-NH-	–19.89	NI	None	>100
**1g**	OH	OH	COOH	H	O	-NH-	–28.60	NI	Partial	>100
**1h**	OH	OH	OH	OH	O	-CH_2_–	–24.17	NI	Partial	>100
**1i**	OH	OH	OH	OH	O	-NHCH_2_CH_2_-	–25.03	NI	Partial	>100
**1j**	OH	OH	OH	OH	S	-NH-	–26.87	NI	Complete	0.41 ± 0.3

a Predicted using the SeeSAR HYDE scoring function.

b The planktonic growth of *SM*-WT was measured at 600 nm.

c Biomass of *SM*-WT + *CA* dual-species biofilms measured at OD_562_ using the crystal violet protocol. NI indicates that no inhibition was observed. Data for biofilms and growth are from three independent experiments. Statistical significance was tested with one-way ANOVA.

All analogs (**1a**–**j**) were initially screened at a concentration of 100 μM to assess the inhibition of biofilm and/or growth. No inhibition of planktonic growth was noted at 100 μM for all compounds. Most compounds exhibited varying degrees of dual-species biofilm inhibition from 0.41 μM to >100 μM (Table 1). Compounds **1b**–**e** and **1j** completely suppressed biofilm formation, whereas compounds **1a** and **1g**–**i** showed partial inhibition, and compound **1f** had no effect. The active compounds were further evaluated to obtain their IC_50_ values ranging from 0.41 μM to >100 μM. Two analogs that were more active than **SN195** are **1b** and **1j**. Compound **1b** that lacked *m*-OH on ring A exhibited an IC_50_ value of 12.9 μM (Figure 5A), and the thiourea analog **1j** with an IC_50_ value of 0.41 μM emerged as the most potent analog (Figure 5B). Fluorescence microscopy images on both **1b** and **1j** show that they essentially abrogate biofilm formation above their IC_50_ values (Figure 5D,E). All three strains tested at 100 μM of **1b** and **1j** had negligible effect on the growth of commensals *S. gordonii*, *S. sanguinis*, and *S. parasanguinis* (Figure 5C). In contrast, the same compounds inhibited the growth of *SM* by approximately 10–15%. Notably, screening the synthetic precursors of analogs **1a**–**j** with protected OH groups in the same dual-species biofilm assay resulted in no activity. Data for three precursors of our most active compounds **SN195**, **1b**, and **1j** is included in the Supporting Information (Table S2).

**Figure 5:**
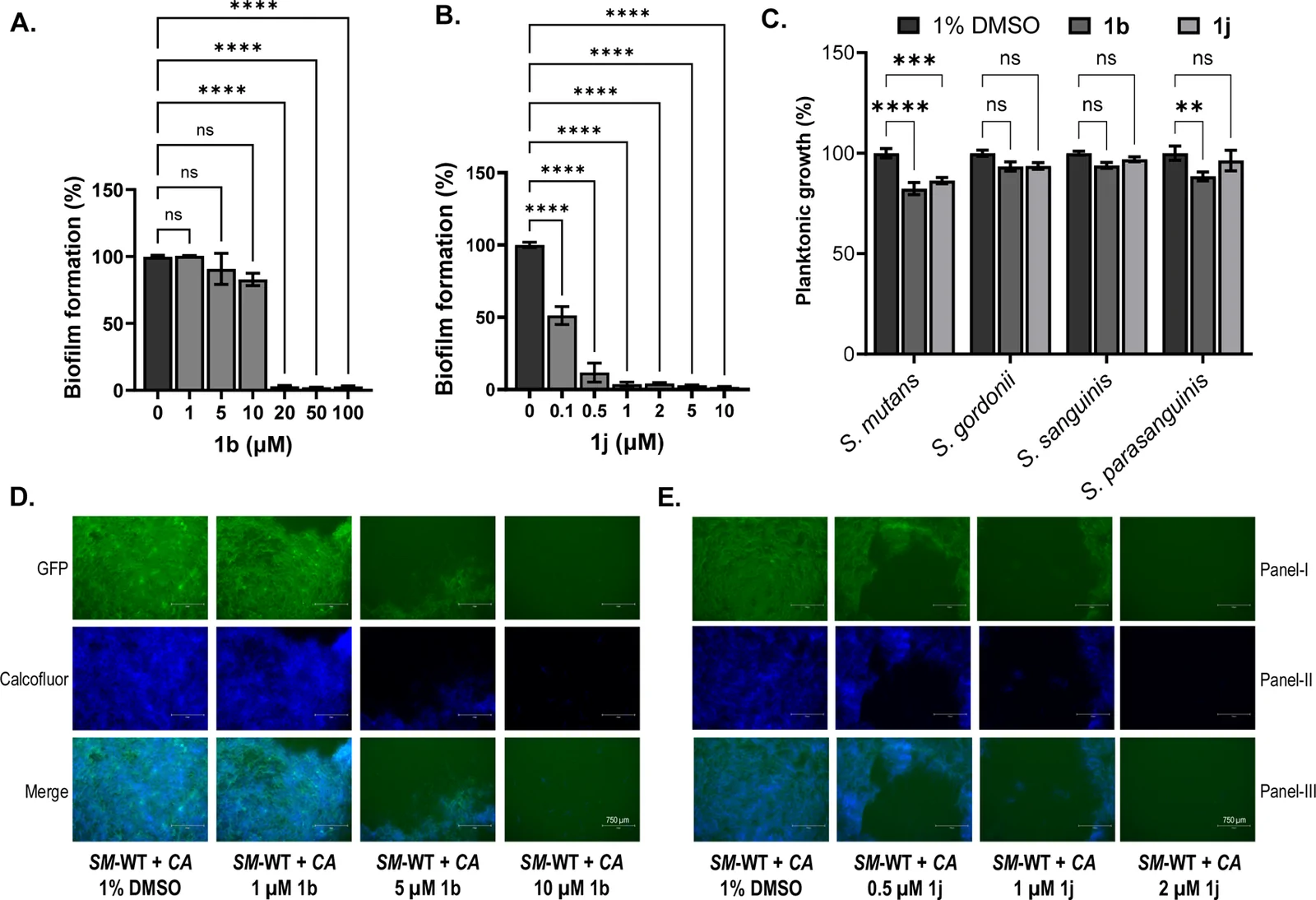
(A) Dose-dependent inhibition of *SM*-WT + *CA* dual-species biofilms by treatment with indicated concentrations of **1b** determined by crystal violet staining at OD_562_, where *SM*-WT + *CA* was normalized to 100%. (B) Dose-dependent inhibition of *SM*-WT + *CA* dual-species biofilm by treatment with indicated concentrations of **1j** determined by crystal violet staining at OD_562_, where *SM*-WT + *CA* was normalized to 100%. (C) *S. mutans* UA159, *S. gordonii* DL1, *S. sanguinis* SK36, and *S. parasanguinis* FW213 were co-incubated with compounds **1b** or **1j** at 100 μM, and their growth was measured at OD_600_. (D) Panel I: Representative fluorescent images of *SM*-WT + *CA* dual species biofilms treated with various concentrations of **1b** using GFP fluorophore. Panel II: Representative images of *SM*-WT–*CA* dual species biofilms treated with various concentrations of **1b** generated using Calcofluor White as fluorophore. Panel III: Merged images. (E) Panel I: Representative fluorescent images of *SM*-WT + *CA* dual species biofilms treated with various concentrations of **1j** using GFP fluorophore. Panel II: Representative images of *SM*-WT–*CA* dual species biofilms treated with various concentrations of **1j** generated using Calcofluor White as fluorophore. Panel III: Merged images. The scale bar measures 750 nm. Each biofilm and growth assay was conducted in triplicate, and statistical significance was determined using one-way ANOVA or two-way ANOVA, ns = *p* > 0.05, ** = *p* < 0.01, *** = *p* < 0.001, **** = *p* < 0.0001.

The optimized lead compounds **1b** and **1j** (Figure 6A) underwent docking analysis with SeeSAR 13.0.1 to further investigate their binding modes within the **SN195** binding site. Both analogs occupied the same binding site as **SN195** but exhibited slightly altered binding modes compared to **SN195** in the crystal structure, engaging several critical residues (Figure 6B) and introducing a few additional interactions to enhance their binding. In the case of analog **1b**, the SeeSAR model indicates that Ring A is positioned similarly to that of **SN195**, interacting with the Ca^2+^ ion. Ring B stretched into the phenylalanine pocket instead of maintaining the same orientation as Ring B of **SN195**. One nitrogen in the urea rests directly between Ser762 and Arg824, allowing H-bonding. The *m*-OH on Ring B interacts with Asn820 and the carbonyl O on Asp512. The *p*-OH on Ring B engaged both Asp512 and Thr651 in a H-bonding network. While the engagement of residues Ser 697 and Ser767 was absent in **1b**, the interaction of Ring B with Asn820 along with Asp512 and Thr651 in the phenylalanine pocket likely accounted for its marginally enhanced activity compared to **SN195**. Our SeeSAR model indicates that Ring A of the most active thiourea analog **1j** (pink), adopted a different orientation, turning away from the Ca^2+^ ion and instead interacting with Ser762 and by π–π stacking with Trp816. Ring B of **1j** adopted a similar orientation to that of **SN195** interacting at the *m*-OH of Ring B to form a strong H-bond with Ser767. The S atom of thiourea interacted with the conserved Ca^2+^ ion and maintained the hydrogen bonding interaction with Ser697. Furthermore, the S atom formed two H-bonds with Ser697 and Asp760, leading to enhanced binding and significantly increased activity. The observed enhanced activity can be likely attributed to these additional interactions in comparison to **SN195**.

**Figure 6:**
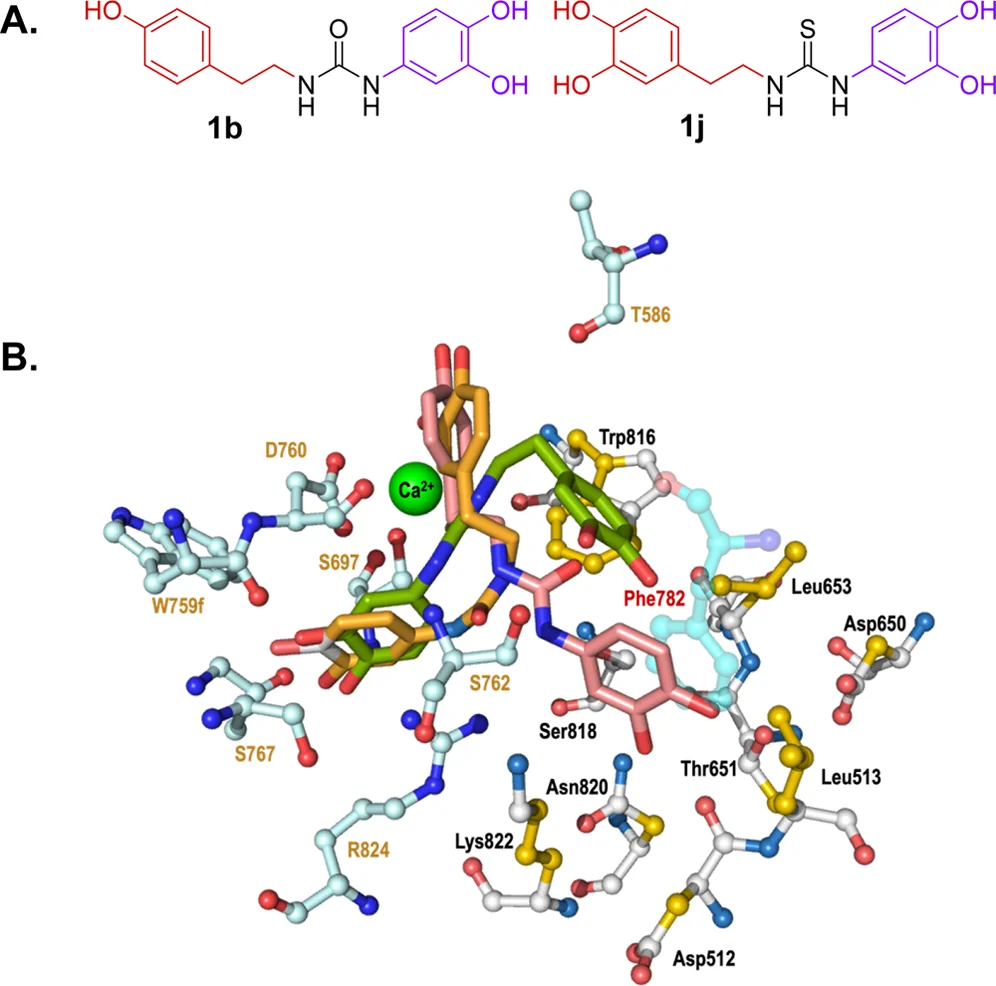
(A) Structures of the more active analogs identified from SAR studies **1b** and **1j**. (B) Docking models of compounds **1b** (salmon) and **1j** (green) in the **V** ^**AgI/II**^ binding site compared with the crystal structure of **SN195** (orange) in the **V** ^**AgI/II**^ **+SN195** cocrystal structure. Docking analysis was carried out with SeeSAR 13.0.1.

Multiple structure–activity relationships were identified by correlating the IC_50_ values of analogs with their anticipated binding site interactions. These observed structure–activity relationships primarily pertained to the presence or absence of OH groups at specific positions on the rings, the substitution of OH groups with other polar moieties such as NH_2_, NO_2_, COOH, or COOMe, the alteration of the linker length, and variation in linker functional groups. The inactive analog **1a**, which lacks a *m*-OH group on ring B, underscores the significance of the H-bond contributed by this group to Ser767. Analogs **1f** and **1g** in which the *p*-OH on ring B was replaced with *p*-NH_2_ and *p*-COOH groups also exhibited IC_50_ values of >100 μM. The reduced H-bonding capacity of *p*-NH_2_ together with steric hindrance from the bulkiness of the *p*-COOH at this position could be two contributing factors for the reduced activity. Additionally, at neutral pH, the *p*-COOH would exist as a carboxylate, reducing the ability of this substituent to bind into the hydrophobic pocket. The substitution of the *p*-OH group on ring B with *p*-NO_2_ (**1c**) and *p*-COOMe (**1d**) groups resulted in a decrease in activity with IC_50_ values of 55.3 μM and 32.9 μM, respectively. This decrease could be attributed to the weaker H-bonding of the NO_2_ and COOMe groups in comparison to the OH group, alongside the unfavorable geometry of the NO_2_ and COOMe groups resulting from their bulkiness leading to diminished binding. Substituting the urea with an amide in **1h** resulted in the loss of biofilm inhibition, indicating that the urea NH group next to the aromatic ring and its interaction with Ser762 plays a crucial role in the activity. Increasing the linker length by two C atoms in **1i** proved to be disadvantageous, resulting in diminished biofilm activity with an IC_50_ value of >100 μM, suggesting that the longer linker may have introduced steric hindrance within the site due to the increased molecular size.

Anticariogenic effects of **1b** and **1j** together with the lead compound **SN195** were evaluated using a dual-species caries model by co-infecting rats with *SM*-UA159 and *CA-*SC5314 in the presence of 5% sucrose to induce caries.^4,32^ Treatment with 100 μM of **SN195**, **1b**, or **1j** led to statistically significant reduction in buccal caries scores compared with untreated infected controls (Figure 7A). Comparable reduction in sulcal caries scores was also observed (Figure 7B). The decrease in buccal caries (enamel, Ds, Dm, and Dx) was particularly notable, with treated groups exhibiting carious lesions in all four areas akin to the uninfected control group. Reductions in sulcal carious lesions were comparable to the enamel and Ds scores, closely aligning with those of the uninfected controls, although the effects on Dm and Dx were somewhat less pronounced. Essentially these compounds were able to disrupt *SM*–*CA* interactions, resulting in caries scores comparable to the uninfected group. Thus, treatment with **SN195**, **1b**, or **1j** significantly reduced caries scores relative to untreated control groups, and the reduction was comparable to that achieved with 250 ppm NaF.

**Figure 7:**
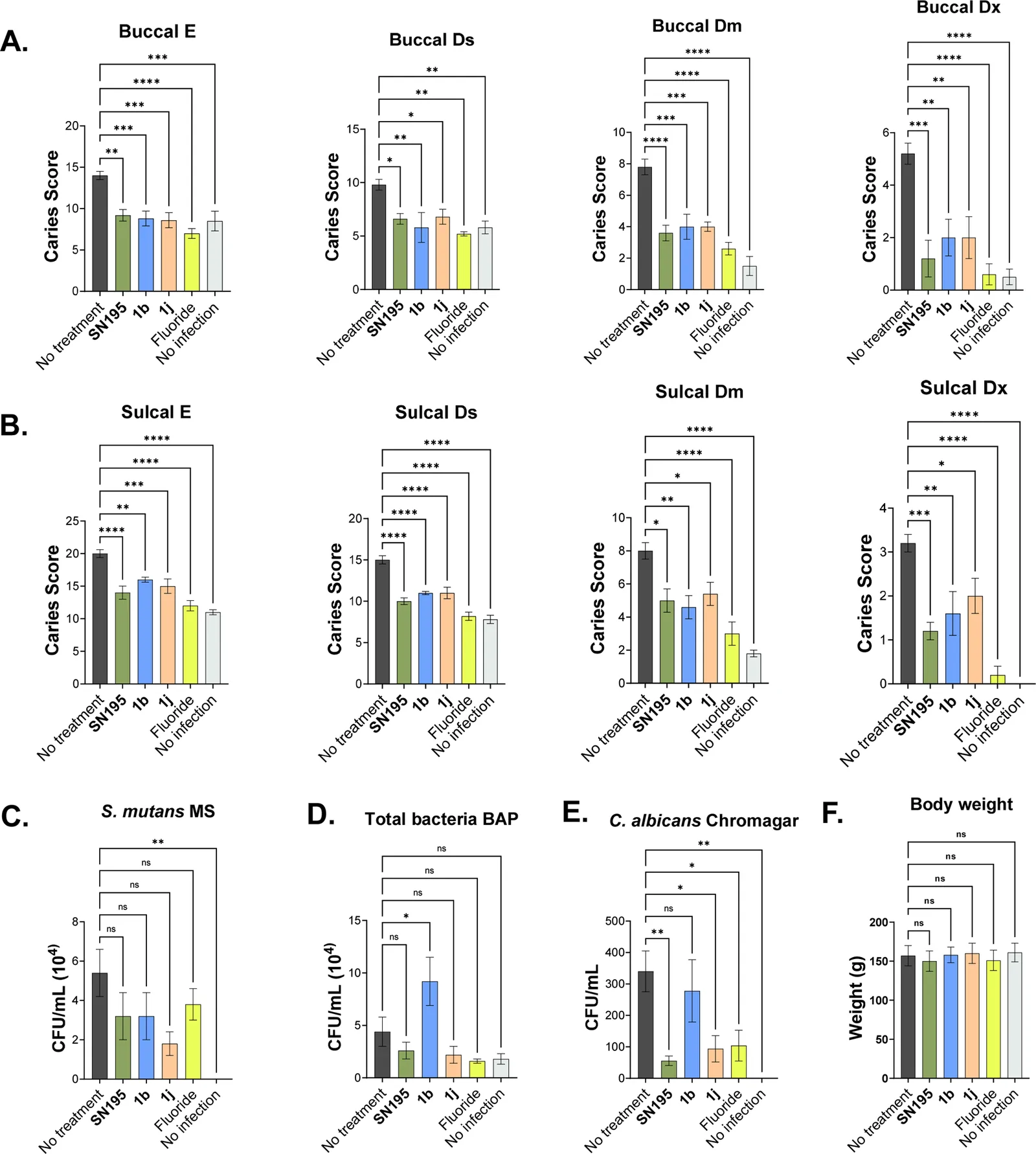
Rodent model of dental caries evaluating the effect of **SN195**, **1b**, or **1j**. (A) Significant reduction in buccal carious scores was observed. Enamel (E); Dentinal slight (Ds); Dentinal moderate (Dm); Dentinal extensive (Dx). (B) Significant reduction in sulcal carious scores was observed. Enamel (E); Dentinal slight (Ds); Dentinal moderate (Dm); Dentinal extensive (Dx). (C) *S. mutans* colonization CFU/mL using Mitis Salivarius agar (MS). (D) Total bacteria colonization CFU/mL using blood agar plates (BAPs). (E) *C. albicans* colonization CFU/mL. (F) Body weights of the animals. Statistical significance was determined using one-way ANOVA. ns = *p* > 0.05, * = *p* < 0.05, ** = *p* < 0.01, *** = *p* < 0.001, **** = *p* < 0.0001.

CFU analyses showed that each of the compounds resulted in diminished levels of *SM*, yet none of them were statistically significant (Figure 7C,D). Except for **1b** treatment, both **SN195** and **1j** considerably reduced *CA* levels, implying that these two compounds are more effective against dual-species biofilms (Figure 7E). Treatment with compounds at 100 μM did not cause weight loss, indicating no observable toxicity in rats (Figure 7F). These findings indicate that small molecule inhibitors can selectively target *SM* surface adhesins, reducing the formation of dysbiotic biofilms and lowering the disease burden of dental caries.

In conclusion, the results of this study suggest that *SM*-AgI/II plays a key role in mediating interspecies interactions between *SM* and *CA*. The lead compound **SN195** inhibited *SM* single-species and *SM*–*CA* dual-species biofilms without affecting their growth or that of commensals. Target engagement was supported by the X-ray crystal structure of the **V** ^**AgI/II**^ **+ SN195** complex. The SAR studies guided by the cocrystal structure led to the identification of more potent biofilm inhibitors, **1b** and **1j**. In a rat caries model, compounds **SN195**, **1b**, and **1j** demonstrated significant anticariogenic activity. These findings suggest that biofilm inhibitors produced in this study could be used to prevent and treat dental caries by targeting *SM*’s surface adhesins. More crucially, our study showed that we can target and develop pathogenic species inhibitors without impacting oral commensal bacteria. The precise mechanism of the involvement of *SM* AgI/II in the interaction with *CA* and the details on the surface components of *CA* partnering with *SM* in this interaction are still elusive, and additional studies are needed to discern the details of the mechanisms of this interaction.

## Supplementary Material



## Notes

Description of organic synthesis and experimental procedures for microbiological studies, dual-species rat caries model, structural studies, and copies of ^1^H NMR spectra, ^13^C NMR spectra, and HPLC chromatograms of all new compounds (PDF)

## Notes

Atomic coordinates and structure factors for the **V** ^**AgI/II**^ **-SN195** cocrystal structure are available in the Protein Data Bank under PDB ID 6UBV.

## List of Abbreviations

AgI/II: Antigen I/II
bs: broad singlet
*CA*: *Candida albicans*
CDM: chemically defined medium
CFU: colony forming unit
ECC: early childhood caries
DMSO: dimethyl sulfoxide
eDNA: extracellular DNA
EPS: extracellular polysaccharide
GFP: green fluorescent protein
Gp340: glycoprotein 340
Gtf: glucosyltransferase
HRMS: high resolution mass spectrometry
IC_50_: half maximal inhibitory concentration
NMR: nuclear magnetic resonance
SAR: structure–activity relationship
SAG: salivary agglutinin
SDS-PAGE: sodium dodecyl sulfate polyacrylamide gel electrophoresis
SRCR peptide: scavenger receptor cysteine-rich peptide
SPR: surface plasmon resonance
*SG*: *Streptococcus gordonii*
*SM*: *Streptococcus mutans*
*SS*: *Streptococcus sanguinis*
THB: Todd Hewitt Broth.
