# Supplementary material for: Targeting Streptococcus mutans Antigen I/II To Inhibit Dual-Species Biofilms with Candida albicans
Source: ACS Med Chem Lett. 2026 Jul 27;17(8):1878–87. doi: 10.1021/acsmedchemlett.6c00275 (PMC13488099; doi:10.1021/acsmedchemlett.6c00275)

Supporting Information

Targeting *Streptococcus mutans* antigen I/II to inhibit dual-species biofilms with *Candida albicans*

B. Owen Garrett[1], Parvathy Babu[1], Bhavitavya Nijampatnam[1], Abhishek Govindan[1], Norbert Schormann[2], Manisha Patel[2], Gregory Harber[3], Suzanne M. Michalek[3], Hui Wu[6], Champion Deivanayagam[2,4]*, and Sadanandan E. Velu[1,4,5]*.*

[1]Department of Chemistry, [2]Department of Biochemistry and Molecular Genetics, [3]Department of Microbiology, [4]Global Center for Craniofacial Oral and Dental Disorders, University of Alabama at Birmingham, Birmingham, AL 35294, USA, [5]Center for Clinical and Translational Sciences, University of Alabama at Birmingham, Birmingham, AL 35294, USA.

[6]Department of Integrative Biomedical and Diagnostic Sciences, Oregon Health and Science University, Portland, OR 97239, USA.

Contents:

1. Microbial strains, culture conditions and assay descriptions……………………… S2
2. *S. mutans* single species biofilm assay…………………………………………. S2
3. *S. mutans – C. albicans* dual-species biofilm assay……………………………… S2
4. *S. mutans*, *S. gordonii, S. sanguinis, and S. parasanguinis* growth assays……….. S2
5. Structural studies on **SN195** in $V^{AgI/II}$ binding site……………………………... S3
6. Dual-species biofilm inhibition activities of the precursor compounds…………... S4
7. Organic synthesis and experimental procedures……….……………………….. S4
8. Rodent model studies with compounds **SN195**, **1b**, and **1j**……………………….. S13
9. References……………………………………………………………………. S14
10. ^{1}H NMR and ^{13}C NMR spectra of compounds……………………..…...………S15
11. HPLC chomatograms of compounds……………..…………………...………S37

METHODS

1. Microbial strains, culture conditions and assay descriptions

Streptococcus strains *S. mutans* UA159, *S. sanguinis* SK36, *S. gordonii* DL1, and *S. parasanguinis* FW213 were grown statically for 24 h at 37 °C, 5 % CO_2 in autoclaved Todd Hewitt Broth (THB) (5 mL). Green fluorescent protein tagged (GFP) *S. mutans* UA159 (*SM*) was used as the wild type (WT) along with its gene deletion strain (*SM*-ΔAgI/II) for all the biofilm studies. *C. albicans* SC5314 (*CA*) strain *was* grown in Yeast-Peptone-Dextrose broth (YPD). Biofilms were grown in the RPMI 1640 media containing HEPES, glutamine, and Phenol Red (Gibco™).

Colony forming units (CFUs) for *SM* and *CA* were also determined as follows. Homogenized biofilm was prepared, where 10-fold dilutions were made, and thereafter each dilution was grown in petri dishes containing Gold's media in 1 % agar. A similar approach was carried out for *CA*, where Candida CHROMagar™ plates were used in our rodent model studies. Plated Petri dishes were grown at 37 °C in 5 % CO_2 for 24 or 36 h, respectively, for *SM* and *CA*.

Fluorescent imaging was performed on an EVOS5000 imaging system at 4x magnification, where *SM* was visualized using GFP, and *CA* was visualized after staining with calcofluor white. All fluorescence measurements were determined using 365 nm excitation and 425 nm emission. All wells containing compounds also contained 1 % DMSO.

2. *S. mutans* single species biofilm assay

Stock solutions prepared in chemically defined medium (CDM, Hazleton Research Products Inc.) were used to examine biofilm as described previously [1, 2]. Biofilm inhibition assays were performed for various concentrations of compounds using polystyrene microtiter Corning® 96-well Clear Flat Bottom Polystyrene (3370) in triplicate and incubated at 37 ºC and 5 % CO_2 for 16 h. Subsequently, the plate was gently washed with water, stained with crystal violet, and thereafter dissolved in 200 µL of 30 % acetic acid. The absorbance at 562 nm was used to determine biomass of biofilms. IC_{50} values (biofilm inhibition) of each compound were evaluated using various concentrations, and their respective values were calculated using GraphPad Prism (v 10.6.1).

3. *S. mutans – C. albicans* dual-species biofilm assay

From overnight cultures, *SM* and *CA* were reinoculated into fresh cultures (THB for *SM* and YPD for *CA*), and grown until an OD_{600} of 0.4 for *SM* and an OD_{600} of 0.65 for *CA*. Only *SM* culture was centrifuged and resuspended in 1 ml of RPMI media. Thereafter, 10 µl of each *SM* and *CA* culture were transferred into RPMI media to a total volume of 200 µL in each well, and biofilms were grown in 96-well plates for 16 h at 37 °C in 5 % CO_2. Biofilm biomass and CFU's were quantified as described in the earlier section.

4. *S. mutans*, *S. gordonii, S. sanguinis, and S. parasanguinis* growth assays

Effects of compounds on the planktonic growth of *SM* and commensal bacterial species, *S. gordonii, S. sanguinis, and S. parasanguinis* were evaluated using the growth assay as described previously [1]. Each inhibitor was used at a concentration of 100 µM and experiments were conducted in triplicates. All growth analysis were performed at OD_{600} and reported.

5. Structural studies on SN195 in $V^{AgI/II}$ binding site

To determine engagement of **SN195** and discern its binding site interactions an X-ray crystal structure of **SN195** in complex with the $V^{AgI/II}$ has been resolved. The apo protein of $V^{AgI/II}$ crystallized in space group $P2_12_12_1$ with four non-crystallographically related molecules (chains A, B, C and D) within the asymmetric unit (6TZL) These crystals were soaked for 1-2 hours with 100 µM (final concentration) of the inhibitor **SN195**. Data collected were reduced and the structure was resolved by molecular replacement (**Table S1**). Electron difference densities were observed in the vicinity of the pocket to which we had designed the inhibitor. After refining the protein residues of $V^{AgI/II}$ first, the **SN195** compound was fitted into electron difference density after which the protein co-complex structure PDB was further refined. Real space correlation coefficients (RSCC) for **SN195** are 0.80 in chains ACD and 0.75 in chain B. Two different conformations were observed for **SN195**. The structure has been deposited into the Protein Data Bank.

Table S1. Data collection and refinement statistics for $V^{AgI/II}$ in complex with **SN195** (6UBV)

Data collection	**6UBV**
Space Group	$P2_12_12_1$
Unit cell parameters [Å]	a = 65.81, b = 132.70, c = 243.82
Resolution [Å]	29.72 - 2.70 (2.78 - 2.70)
Unique reflections	56119 (3400)
Completeness [%]	94.0 (71.3)
Multiplicity	4.2 (2.0)
R_{merge} [%]	10.8 (41.9)
R_{pim} [%]	5.8 (32.1)
$CC_{1/2}$ (2.78 - 2.70Å)	0.816
CC* (2.78 - 2.70Å)	0.948
I/σ(I)	11.6 (1.7)
Refinement	
Resolution [Å]	29.73 - 2.70 (2.80 - 2.70)
No. of reflections	56040 (4232)
Completeness [%]	94.0 (71.7)
R_{work} [%]	20.6 (25.3)
R_{free} [%]	24.9 (29.7)
Wilson B [$Å^2$]	25.7
Average B-factors [$Å^2$]	
Overall	26.3 (12619 atoms)
Protein, Waters	26.2 (12122 atoms), 21.1 (380 atoms)
Ligands (SNZ, Ca^{2+}, Na^+, SO_4^{2-}, EDO)	50.8 (88, 4, 3, 10, 12 atoms)
Rmsd bonds [Å]	0.005
Rmsd angles [º]	0.81
CC (Fo-Fc)	0.91
Ramachandran	97.3% favored (no outlier)
Clash score	4.98
Molprobity score	1.39

6. Dual-species biofilm inhibition activities of the precursor compounds

Table S2. Dual-species biofilm inhibition activities of the precursor compounds of the most active analogs.

Compd No	Structure	*SM+CA* Dual-species biofilm IC_{50} (μM) [a]
**4**	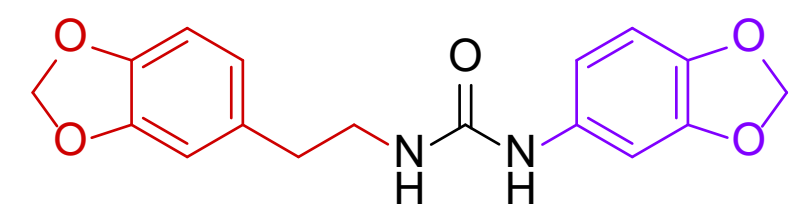	>100
**5b**	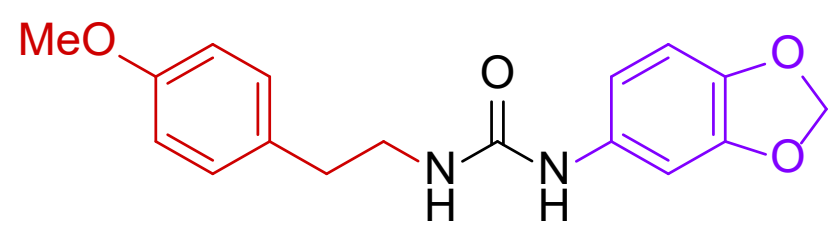	>100
**15**	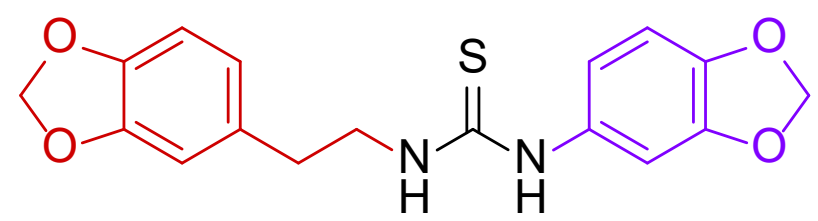	>100

_a Biomass of SM-WT + CA dual-species biofilms measured at OD_{562} using the crystal violet protocol. Data for biofilm inhibition are from three independent experiments. Statistical significance was tested with one-way ANOVA.

7. Organic synthesis

Synthesis of SN195 and its analogs 1a-j

Compound **SN195** is synthesized as shown in **Scheme S1.** Treatment of 3,4-methylenedioxyphenethylamine (**2b**) with 3,4-methylenedioxy phenyl isocyanate (**3a**) in toluene in the presence of Et_3N at room temperature afforded the urea intermediate compound **4**. Removal of the methylenedioxy group from compound **4** using BBr_3 in CH_2Cl_2 at 0 °C resulted in the formation of **SN195** in 82 % yield.

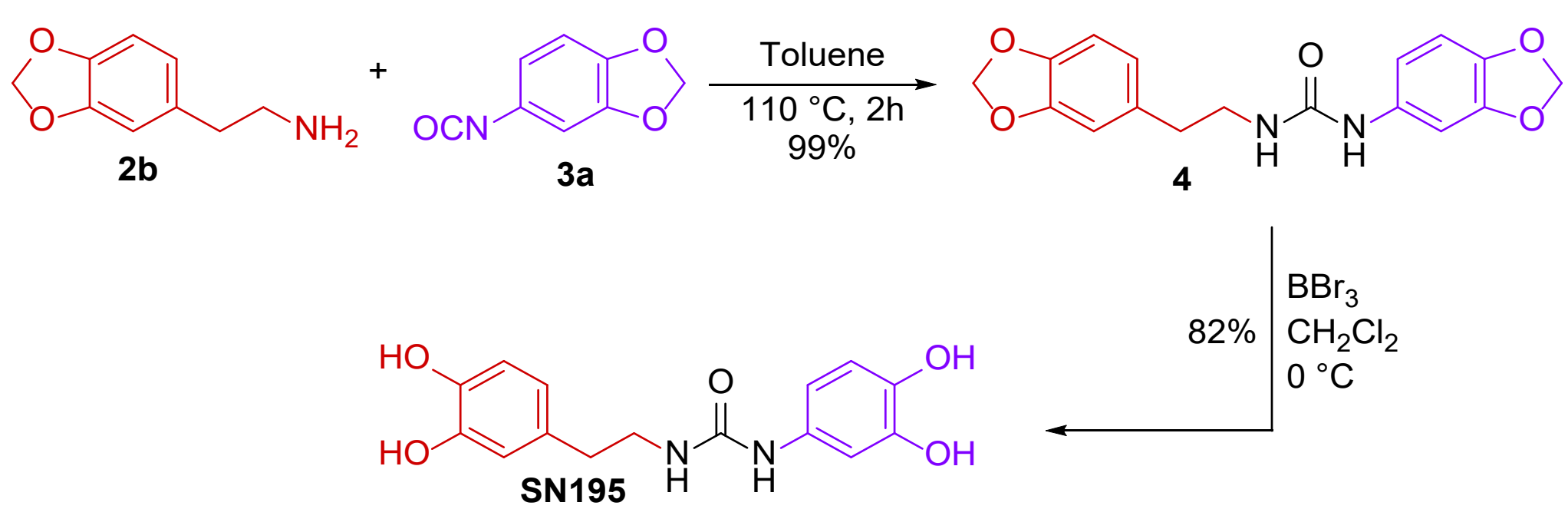

Scheme S1. Synthesis of **SN195**

The analogs **1a-d** were synthesized using a general procedure similar to the one used for the synthesis of **SN195** as shown in **Scheme S2**. The amines **2a-b** were treated with the isocyanates **3a-d** in toulene at room temperature to afford the urea compounds **5a-d** in 30-98 % yield. Removal

of the methylenedioxy and methoxy groups from **5a-d** using BBr_3 in CH_2Cl_2 at 0 °C resulted in the formation of compounds **1a-d** in 65-100 % yield.

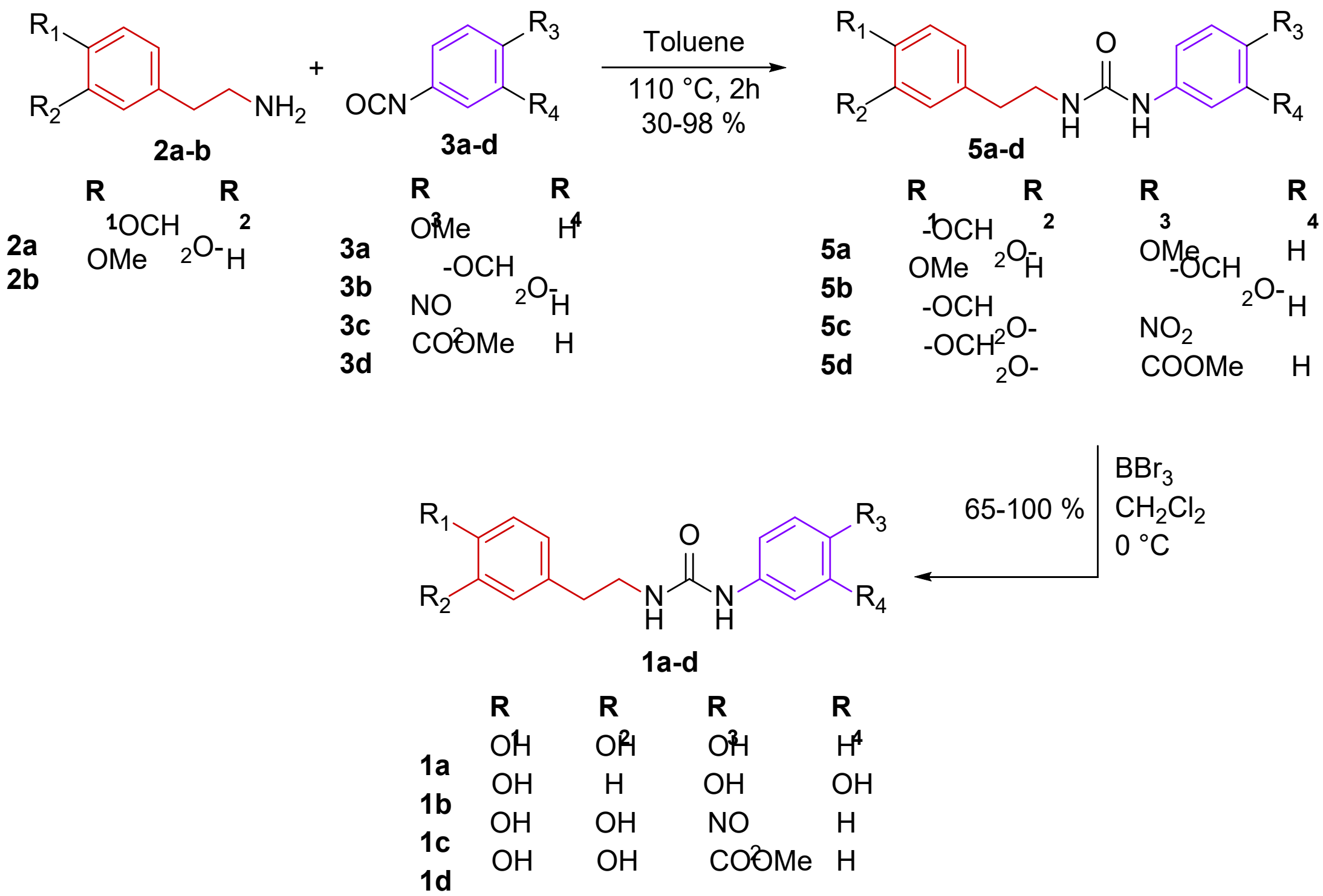

Scheme S2. Synthesis of the analogs **1a-d**

Syntheses of the amino analogs **1e** and **1f**, and the carboxylic acid analog **1g** are outlined in **Schemes S3-5.** Treatment of 4-aminophenethyl amine (**6**) with 3,4-methylenedioxy phenyl isocyanate (**3a**) in toluene at 110 °C afforded the intermediate urea compound **7** in 91 % yield. Removal of the methylenedioxy group from **7** using BBr_3 in CH_2Cl_2 at 0 °C resulted in the formation of **1e** in 100 % yield. Reduction of the nitro group in compound **5c** using H_2 in the presence of Pd/C at room temperature gave the amino compound **8** in 84 % yield. Removal of the methylenedioxy group from **8** using BBr_3 in CH_2Cl_2 at 0 °C resulted in the formation of **1f** in 78 % yield. Hydrolysis of the ester group in compound **5d** using 6N NaOH in MeOH at room temperature afforded the carboxylic acid compound **9** in 84 % yield. Removal of the methylenedioxy group from **9** using BBr_3 in CH_2Cl_2 at 0 °C resulted in the formation of **1g** in 82 % yield.

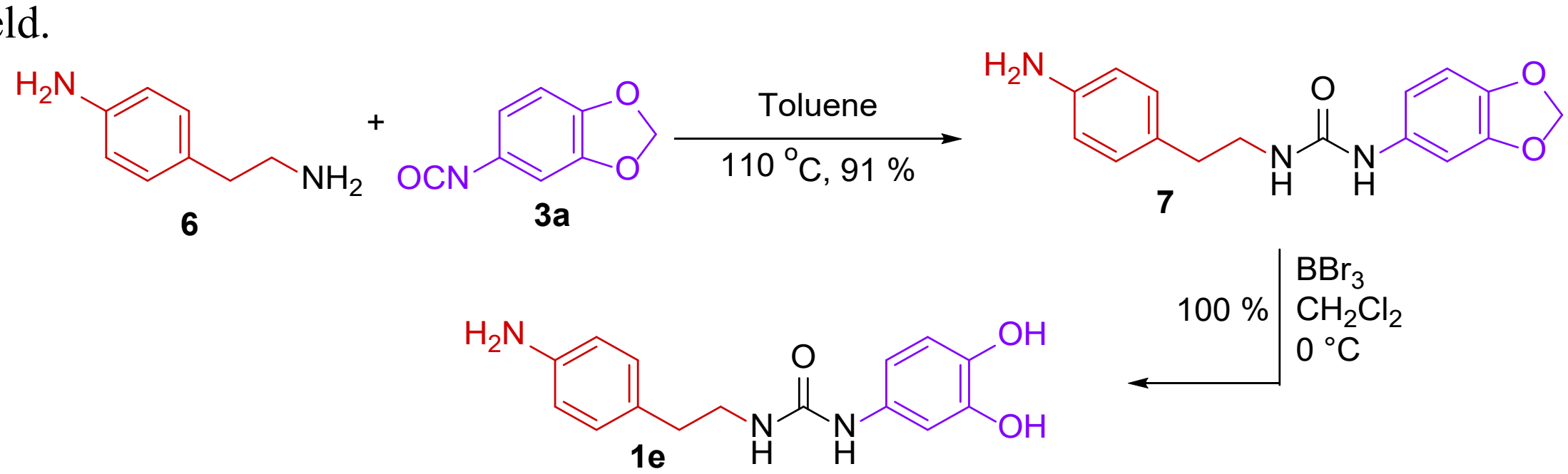

Scheme S3. Synthesis of **1e**

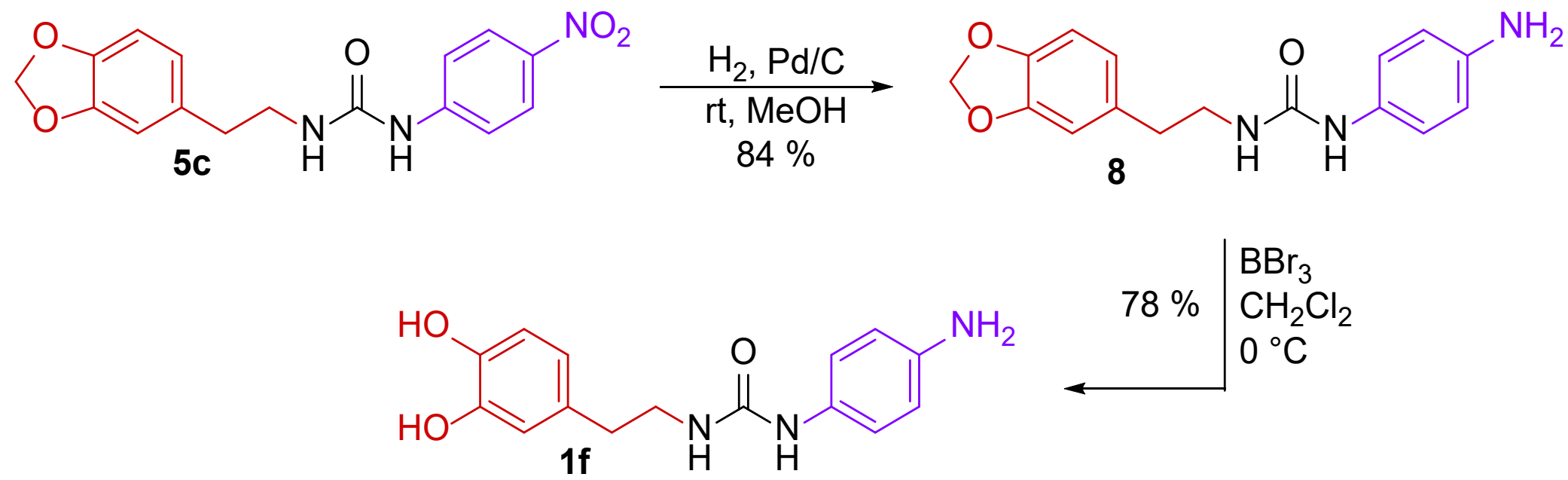

Scheme S4. Synthesis of **1f**

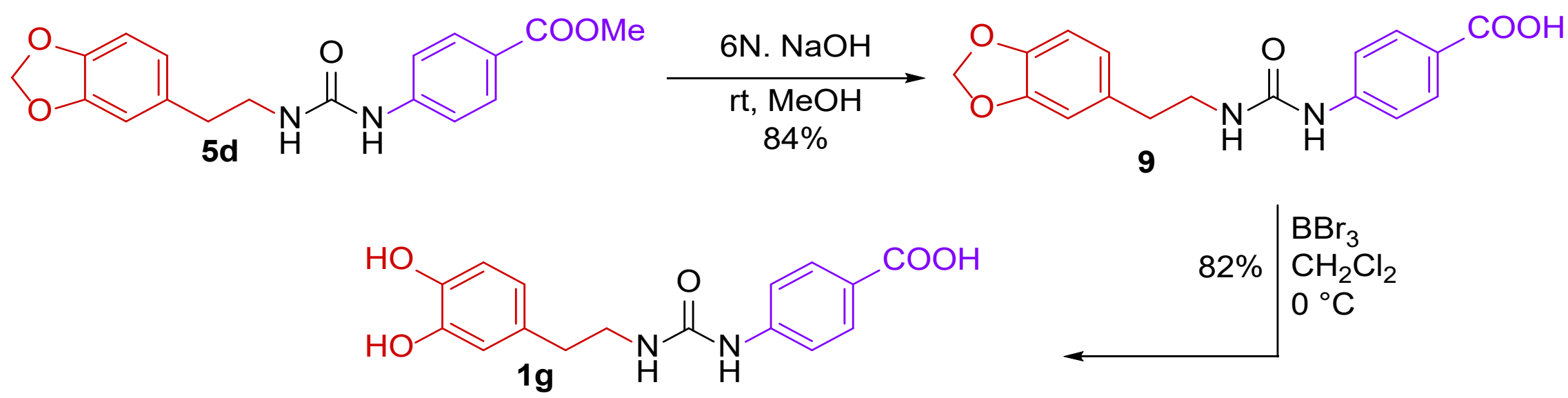

Scheme S5. Synthesis of **1g**

Synthesis of the amide analog **1h** is outlined in **Scheme S6.** Coupling of 3,4-methylenedioxyphenethylamine (**2b**) with 3,4-methlenedioxy phenyl acetic acid (**10**) in the presence of EDC and DMAP in CH_2Cl_2 at 35 ºC gave the amide derivative **11** in 82 % yield. Removal of the methylenedioxy group in **11** using BBr_3 in CH_2Cl_2 at 0 °C resulted in the formation of the amide analog **1h** in 55 % yield. Synthesis of analog **1i** with two extra carbons in the linker is shown in **Scheme S7.** Treatment of 3,4-methylenedioxy phenethyl isocyanate (**12**) with 3,4-methylenedioxyphenethylamine (**2b**) in toluene at 110 °C afforded the urea intermediate **13** in 84 % yield. Compound **13** was treated with BBr_3 in CH_2Cl_2 at 0 °C to remove the methylenedioxy group to give **1i** in 78 % yield.

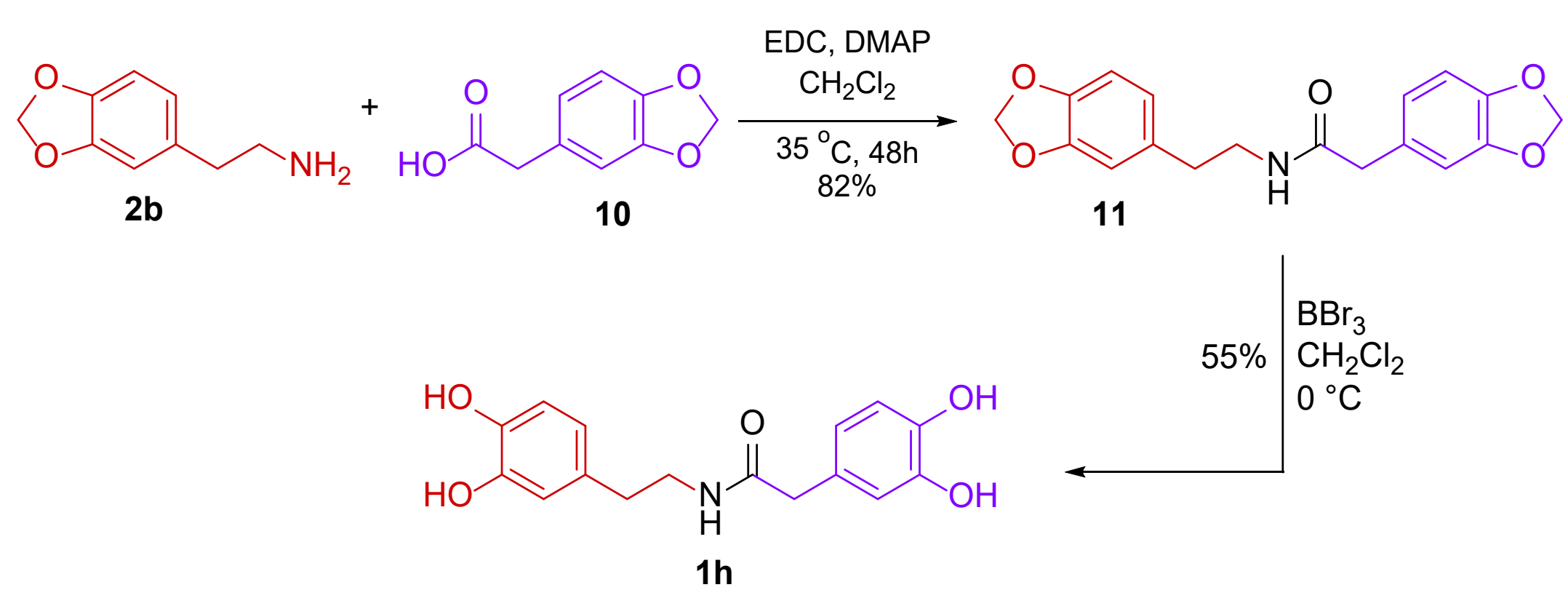

Scheme S6. Synthesis of the amide analog **1h**

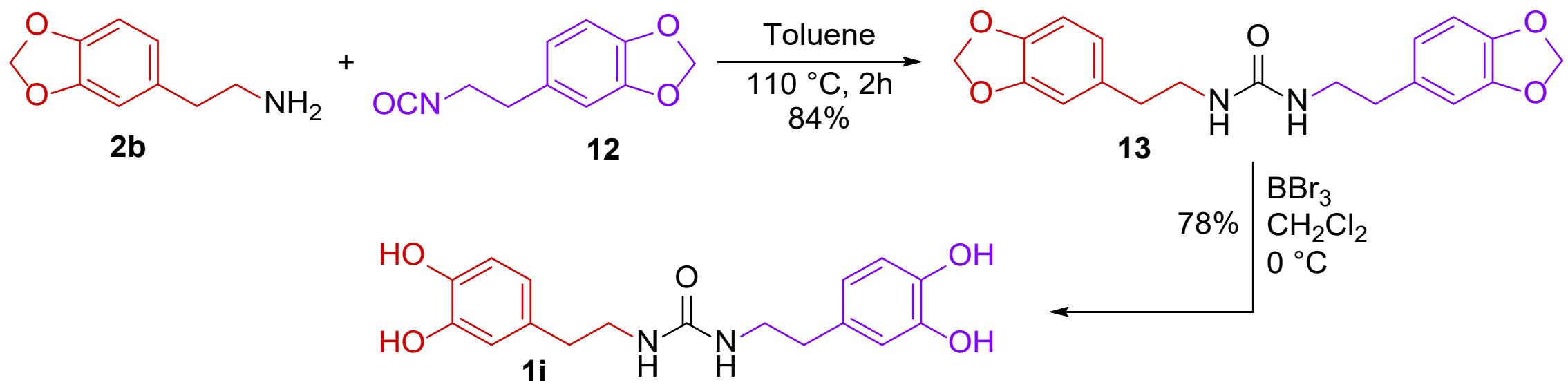

Scheme S7. Synthesis of the extended linker analog **1i**

Synthesis of the thiourea analog **1j** is outlined in **Scheme S8.** Treatment of 3,4-methylenedioxyphenethylamine (**2b**) and 3,4-methylenedioxyphenyl isothiocyanate (**14**) in toluene at room temperature afforded the thiourea intermediate compound **15** in 100 % yield. Removal of the methylenedioxy group from **15** using BBr_3 in CH_2Cl_2 at 0 °C resulted in the formation of **1j** in 39 % yield.

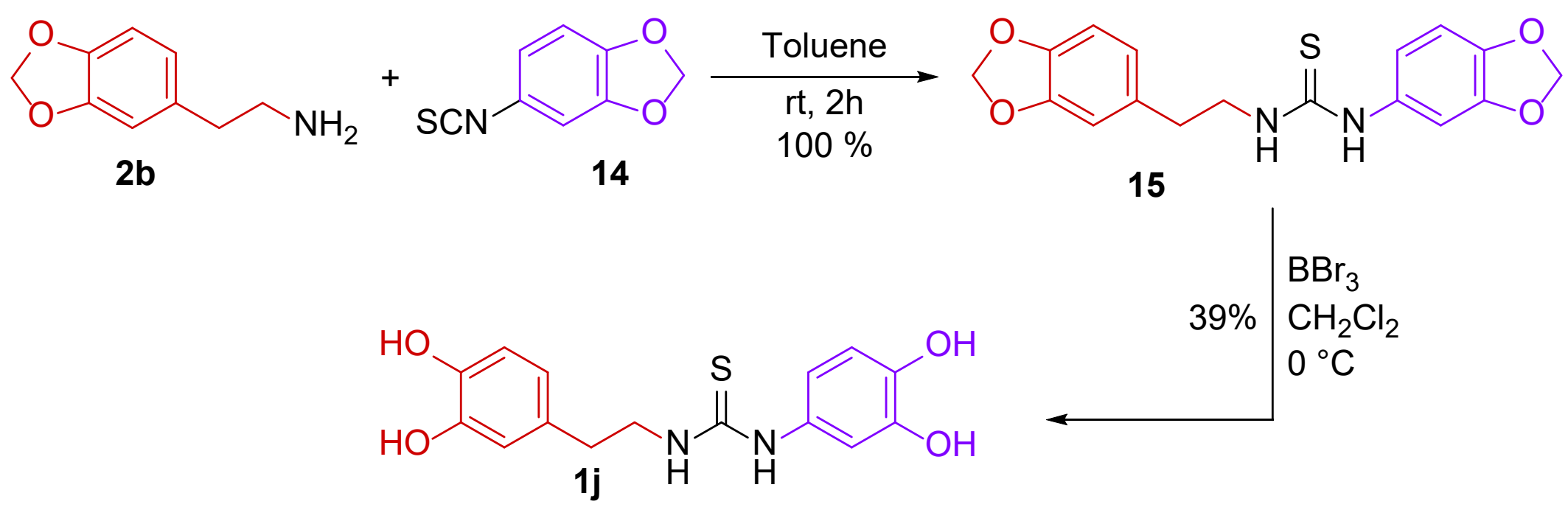

Scheme 8. Synthesis of the thiourea analog **1j**

Experimental procedures

General considerations

^{1}H-NMR and ^{13}C-NMR spectra were recorded on Bruker Avance Neo 400 MHz and Bruker Avance Neo 300 MHz spectrometers using TMS or appropriate solvent signals as internal standard. The chemical shift values are given in parts per million (ppm) relative to the internal standard used and the coupling constants (*J*) are given in Hertz (Hz). High resolution mass spectra (HRMS) were recorded using a Waters AutoSpec-Ultima™ NT magnetic sector mass spectrometer with Electron Impact (EI) Ionization source. The mass analyzer is an electric-magnetic-electric (E-M-E) double focusing sector. Anhydrous solvents used in reactions were purchased in Sure-Seal™ bottles from Sigma-Aldrich chemical company. Other chemical reagents were purchased from Sigma-Aldrich or Fisher chemical companies and used as received. Progress of the reactions were monitored by thin layer chromatography (TLC) using silica gel plates coated with fluorescent indicator (Silicycle, UV254, 25 mm plates). The TLC spots were observed under UV light with wavelengths 254 nm and 365 nm. The reaction products were purified by column chromatography using silica gel (32-63 mm) from Dynamic Adsorbent, Inc. Melting points were determined on a Mel-Temp II

melting point apparatus and are uncorrected. All compounds have ≥95 % purity determined by high-performance liquid chromatography (HPLC). All HPLC traces were obtained using a Shimadzu SPD-M20A system.

Synthetic procedures

***N*-(2*H*-1,3-Benzodioxol-5-yl)-*N'*-[2-(2*H*-1,3-benzodioxol-5-yl) ethyl] urea (4)**

3,4-methylenedioxyphenethylamine **2b** (62 mg, 0.3 mmol), 3,4-methylenedioxyphenyl isocyanate **3a** (50 mg, 0.30 mmol), and Et_3N (0.1 mL, 0.75 mmol) were dissolved in anhydrous toluene, and the reaction mixture was refluxed at 110 ºC for 2 h. TLC analysis using 10 % EtOAc in hexane as eluent revealed the completion of the reaction. The product precipitated upon cooling to room temperature. The resulting solid was filtered, washed with water (50 mL) and hexane (50 mL) to afford pure product **4** as white powder (98 mg, 99 %); Mp. 189-190 ºC; ^{1}H NMR (400 MHz, DMSO-*d*$_6$) δ 8.36 (s, 1H), 7.16 (s, 1H), 6.85-6.80 (m, 2H), 6.76 (d, 1H, J = 8.4 Hz), 6.70-6.62 (m, 2H), 5.98 (t, 1H, J = 5.2 Hz), 5.97 (s, 2H), 5.92 (s, 2H), 3.26, (dt, 2H, J_1 = 7.0 Hz, J_2 = 5.2 Hz), and 2.64 (t, 2H, J = 7.0 Hz); ^{13}C{^{1}H} NMR (100 MHz, DMSO-*d*$_6$) δ 155.7, 147.7, 147.6, 146.0, 141.8, 135.5, 133.8, 122.0, 110.5, 109.5, 108.6, 108.5, 101.12, 101.08, 100.8, 41.2, and 36.0; HRMS calcd for $C_{17}H_{16}N_2O_5$ [M + Na]$^+$: 351.0957, found 351.0960.

***N*-(3,4-Dihydroxyphenyl)-*N'*-[2-(3,4-dihydroxyphenyl) ethyl] urea (SN195)**

To a solution of **4** (50 mg, 0.15 mmol) in CH_2Cl_2 (10 mL), BBr_3 (1 mL, 1M in CH_2Cl_2, 1 mmol) was added and the reaction mixture was stirred at 0 °C for 2 h. TLC analysis using 5 % MeOH in $CHCl_3$ as eluent revealed the complete disappearance of the starting material. Upon completion of the reaction excess BBr_3 was quenched by slow drop-wise addition of MeOH until the evolution of gases ceased, and the solvents were removed under reduced pressure to obtain the crude product, which was purified by column chromatography over Si gel using 0-5 % MeOH in $CHCl_3$ as eluent to afford pure **SN195** as an off-white solid (38 mg, 82 %); Mp. 69-71 °C; ^{1}H NMR (400 MHz, MeOH-*d*$_4$) δ 6.85 (s, 1H), 6.65-6.85 (m, 3H), 6.50-6.55 (m, 2H), 3.35 (t, 2H, J = 6.8 Hz), and 2.64 (t, 2H, J = 6.8 Hz); ^{13}C{^{1}H} NMR (100 MHz, MeOH-*d*$_4$) δ 159.0, 146.4, 146.2, 144.7, 142.5, 132.7, 132.2, 121.1, 116.9, 116.4, 116.3, 113.4, 110.2, 42.7, and 36.7; HRMS calcd for $C_{15}H_{16}N_2O_5$ [M + H]$^+$: 305.1137, found 305.1130.

General procedure A for the urea synthesis

A solution of the amine **2a-b** (1.2 mmol) and isocyanate **3a-d** (1 mmol) in anhydrous toluene (25 mL) was refluxed at 110 ºC for 4 h. Progress of the reaction was monitored by TLC using 25 % EtOAc in hexane as eluent. Upon the completion of the reaction, the reaction mixture was cooled down to room temperature, the resulting solid was filtered, washed with water (50 mL) and hexanes (50 mL) to afford pure urea products **5a-d** as white powder in 30-98 % yield.

1-(2-(Benzo[d][1,3]dioxol-5-yl)ethyl)-3-(4-methoxyphenyl)urea (5a): (114 mg, 30 %); Mp. 178-179 ºC; ^{1}H NMR (400 MHz, DMSO-*d*$_6$) δ 8.26 (s, 1H), 7.26 (d, 2H, J = 9.2 Hz), 6.85-6.78 (m, 4H), 6.68 (dd, 1H, J_1 = 7.6 Hz, J_2 = 1.2 Hz), 5.97 (s, 2H), 5.95 (t, 1H, J = 6.0 Hz), 3.68 (s, 3H), 3.26, (dt, 2H, J_1 = 7.2 Hz, J_2 = 6.0 Hz), and 2.64 (t, 2H, J = 7.2 Hz); ^{13}C{^{1}H} NMR (100 MHz, DMSO-*d*$_6$) δ 155.3, 153.9, 147.2, 145.5, 133.7, 133.4, 121.6, 119.3, 113.9, 109.1, 108.2, 100.7, 55.1, 40.8, and 35.6; HRMS calcd for $C_{17}H_{18}N_2O_4$ [M + Na]$^+$: 337.1164, found 337.1156.

1-(Benzo[d][1,3]dioxol-5-yl)-3-(4-methoxyphenethyl)urea (5b): (358 mg, 93 %); Mp. 187-189 ºC; ^{1}H NMR (400 MHz, DMSO-d_6) δ 8.35 (s, 1H), 7.17-7.13 (m, 3H), 6.87 (d, 2H, J = 8.4 Hz), 6.76 (d, 1H, J = 8.4 Hz), 6.64 (d, 1H, J = 8.4 Hz), 5.98 (t, 1H, J = 5.4 Hz), 5.92, (s, 2H), 3.72 (s, 3H), 3.26, (dt, 2H, J_1 = 7.0 Hz, J_2 = 5.4 Hz), and 2.66 (t, 2H, J = 7.0 Hz); ^{13}C{^{1}H} NMR (100 MHz, DMSO-d_6) δ 157.7, 155.2, 147.1, 141.3, 135.1, 131.4, 129.6, 113.8, 110.1, 108.0, 100.6, 100.4, 55.0, 40.9, and 35.0; HRMS calcd for $C_{17}H_{18}N_2O_4$ [M + Na]$^+$: 337.1164, found 337.1161.

1-(2-(Benzo[d][1,3]dioxol-5-yl)ethyl)-3-(4-nitrophenyl)urea (5c): (797 mg, 98 %); Mp. 190-191 ºC; ^{1}H NMR (400 MHz, DMSO-d_6) δ 9.29 (s, 1H), 8.13 (d, 2H, J = 9.2 Hz), 7.61 (d, 2H, J = 9.2 Hz), 6.86-6.82 (m, 2H), 6.69 (d, 1H, J = 8.0 Hz), 6.39 (t, 1H, J = 5.4 Hz), 5.97 (s, 2H), 3.26, (dt, 2H, J_1 = 6.6 Hz, J_2 = 5.4 Hz), and 2.68 (t, 2H, J = 6.6 Hz); ^{13}C{^{1}H} NMR (100 MHz, DMSO-d_6) δ 154.3, 147.23, 147.17, 145.6, 140.4, 133.1, 125.1, 121.6, 116.8, 109.0, 108.2, 100.7, 40.8, and 35.2; HRMS calcd for $C_{16}H_{15}N_3O_5$ [M + Na]$^+$: 352.0909, found 352.0902.

Methyl 4-(3-(2-(benzo[d][1,3]dioxol-5-yl)ethyl)ureido)benzoate (5d): (354 mg, 84 %); Mp. 191-193 ºC; ^{1}H NMR (400 MHz, DMSO-d_6) δ 8.94 (s, 1H), 7.83 (d, 2H, J = 8.6 Hz), 7.51 (d, 2H, J = 8.6 Hz), 6.86-6.82 (m, 2H), 6.69 (d, 1H J = 8.0 Hz), 6.26 (t, 1H, J = 5.6 Hz), 5.97 (s, 2H), 3.80 (s, 3H), 3.31 (dt, 2H, J_1 = 6.6 Hz, J_2 = 5.6 Hz), and 2.67 (t, 2H, J = 6.6 Hz); ^{13}C{^{1}H} NMR (100 MHz, acetone-d_6) δ 167.0, 155.5, 148.6, 147.0, 146.2, 134.3, 131.3, 123.6, 122.6, 117.8, 109.9, 108.9, 101.8, 51.9, 42.2, and 36.7; HRMS calcd for $C_{18}H_{18}N_2O_5$ [M + Na]$^+$: 365.1113, found 365.1110.

General procedure B for deprotection

Compound **5a-d** (0.3 mmol) was dissolved in a solution of BBr_3 in CH_2Cl_2 (5 mL, 1M) at 0 °C. The reaction mixture was stirred at 0 °C for 2h and then allowed to attain room temperature. TLC analysis using 50 % EtOAc in hexanes as eluent showed complete disappearance of the starting material. Excess BBr_3 in the reaction mixture was quenched carefully by slow drop-wise addition of MeOH until the evolution of gases ceased. The solvents were removed *in vacuo* using a rotary evaporator and the crude product obtained was purified by column chromatography over Si gel using 0-9 % MeOH in $CHCl_3$ as eluent to afford pure deprotected products **1a-d** as crystalline solids in 65-100 % yield.

1-(3,4-Dihydroxyphenethyl)-3-(4-hydroxyphenyl)urea (1a): (52 mg, 87 %); Mp. 171-172 ºC; ^{1}H NMR (400 MHz, acetone-d_6) δ 8.15 (s, 1H), 7.99 (bs, 1H), 7.83 (bs, 1H), 7.67 (s, 1H), 7.24 (d, 2H, J = 8.8 Hz), 6.76-6.66 (m, 4H), 6.53 (d, 1H, J = 8.8 Hz), 5.57 (t, 1H, J = 5.8 Hz), 3.36, (dt, 2H, J_1 = 7.0 Hz, J_2 = 5.8 Hz), and 2.63 (t, 2H, J = 7.0 Hz); ^{13}C{^{1}H} NMR (100 MHz, acetone-d_6) δ 156.6, 153.3, 145.9, 144.3, 133.5, 132.1, 121.3, 120.8, 116.6, 116.0, 115.9, 42.2, and 36.6; HRMS calcd for $C_{15}H_{16}N_2O_4$ [M + Na]$^+$: 311.1008, found 311.1010.

1-(3,4-Dihydroxyphenyl)-3-(4-hydroxyphenethyl)urea (1b): (92 mg, 100 %); Mp. 69-71 ºC; ^{1}H NMR (300 MHz, MeOH-d_4) δ 7.03 (d, 2H J = 8.5 Hz), 6.86 (d, 1H, J = 2.5 Hz), 6.75-6.64 (m, 3H), 6.54 (dd, 2H, J_1 = 2.5 Hz, J_2 = 8.5 Hz), 3.34, (t, 2H, J = 7.1 Hz), and 2.68 (t, 2H, J = 7.1 Hz); ^{13}C{^{1}H} NMR (75 MHz, MeOH-d_4) δ 158.9, 156.8, 146.4, 142.5, 132.6, 131.3, 130.8, 116.3, 116.2, 113.5, 110.3, 42.7, and 36.4; HRMS calcd for $C_{15}H_{16}N_2O_4$ [M + H]$^+$: 289.1188, found 289.1178.

1-(3,4-Dihydroxyphenethyl)-3-(4-nitrophenyl)urea (1c): (41 mg, 65 %); Mp. 84 °C (decomposed); ^{1}H NMR (400 MHz, acetone-*d*6) δ 8.68 (bs, 1H), 8.13 (d, 2H, *J* = 8.4 Hz), 7.79-7.74 (m, 2H), 7.71 (d, 2H, *J* = 8.4 Hz), 6.78-6.71 (m, 2H), 6.56 (d, 1H, *J* = 7.6 Hz), 5.98, (bs, 1H), 3.42, (t, 2H, *J* = 6.8 Hz), and 2.68 (t, 2H, *J* = 6.8 Hz); ^{13}C{^{1}H} NMR (100 MHz, acetone-*d*6) δ 155.3, 148.0, 145.9, 144.3, 142.2, 131.8, 125.7, 120.8, 117.9, 116.6, 116.1, 42.2, and 36.1; HRMS calcd for $C_{15}H_{15}N_3O_5$ [M + Na]$^+$: 340.0909, found 340.0901.

Methyl 4-[3-(3,4-dihydroxyphenethyl)ureido]benzoate (1d): (96 mg, 100 %); Mp. 185 °C (decomposed); ^{1}H NMR (400 MHz, acetone-*d*6) δ 7.87 (d, 2H, *J* = 8.4 Hz), 7.58 (d, 2H, *J* = 8.4 Hz), 6.80-6.71 (m, 2H), 6.53 (d, 1H, *J* = 7.6 Hz), 3.82 (s, 3H), 3.43, (t, 2H, *J* = 6.8 Hz), and 2.68 (t, 2H, *J* = 6.8 Hz); ^{13}C{^{1}H} NMR (100 MHz, acetone-*d*6) δ 167.2, 155.6, 146.2, 145.9, 144.3, 132.0, 131.3, 123.6, 120.9, 117.8, 116.7, 116.2, 51.9, 42.2, and 36.3; HRMS calcd for $C_{17}H_{18}N_2O_5$ [M + H]$^+$: 331.1294, found 331.1290.

1-(4-Aminophenethyl)-3-(benzo[d][1,3]dioxol-5-yl)urea (7)

Prepared using general procedure A starting from amine **6** (0.20 mL, 1.48 mmol) and isocyanate **3a** (200 mg, 1.23 mmol) in anhydrous toluene (40 mL) to obtain **7** (334 mg, 91 %); Mp. 161-163 ºC; ^{1}H NMR (400 MHz, DMSO-*d*6) δ 8.36 (s, 1H), 7.16 (d, 1H, *J* = 1.6 Hz), 6.86 (d, 2H, *J* = 8.2 Hz), 6.75 (d, 1H, *J* = 8.4 Hz), 6.63 (dd, 1H, J_1 = 8.4 Hz, J_2 = 1.6 Hz), 6.49 (d, 2H, *J* = 8.2 Hz), 5.94 (t, 1H, *J* = 4.8 Hz), 5.92 (s, 2H), 4.88 (s, 2H), 3.19, (dt, 2H, J_1 = 7.2 Hz, J_2 = 4.8 Hz), and 2.53 (t, 2H, *J* = 7.2 Hz); ^{13}C{^{1}H} NMR (100 MHz, DMSO-*d*6) δ 155.2, 147.1, 146.8, 141.3, 135.1, 129.0, 126.3, 114.0, 110.0, 108.0, 100.6, 100.3, 41.1, and 35.1; HRMS calcd for $C_{16}H_{17}N_3O_3$ [M + H]$^+$: 300.1348, found 300.1351.

1-(4-Aminophenethyl)-3-(3,4-dihydroxyphenyl)urea (1e)

To a solution of **7** (100 mg, 0.33 mmol) in CH_2Cl_2 (10 mL), a solution of BBr_3 in CH_2Cl_2 (1 mL, 1M) was added at 0 °C. The reaction mixture was stirred for 2 h and then allowed to attain room temperature. TLC analysis using 10 % MeOH in $CHCl_3$ as eluent showed disappearance of the starting material. Upon completion of the reaction, excess BBr_3 in the reaction mixture was quenched by slow drop-wise addition of MeOH until the evolution of gases ceased. Solvents were removed *in vacuo* and the crude product obtained was purified by column chromatography over Si gel using 0-10 % MeOH in $CHCl_3$ as eluent to afford pure compound **1e** as a black solid (96 mg, 100 %); Mp. 237 °C (decomposed); ^{1}H NMR (400 MHz, MeOH-*d*4) δ 7.37 (d, 2H, *J* = 7.8 Hz), 7.29 (d, 2H, *J* = 7.8 Hz), 6.81 (s, 1H), 6.62 (d, 1H, *J* = 8.2 Hz), 6.53 (d, 1H, *J*= 8.2 Hz), 3.66 (t, 2H *J* = 6.8 Hz 3.29 (bs, 2H),, and 2.81 (t, 2H, *J* = 6.8 Hz); ^{13}C{^{1}H} NMR (100 MHz, MeOH-*d*4) δ 158.8, 146.3, 142.3, 142.2, 132.8, 131.7, 129.9, 124.1, 116.3, 113.2, 110.0, 42.1, and 36.8; HRMS calcd for $C_{15}H_{17}N_3O_3$ [M + H]$^+$: 288.1348, found 288.1353.

1-(4-Aminophenyl)-3-[2-(2H-1,3-benzodioxol-5-yl)ethyl]urea (8)

To a solution of **5c** (189 mg, 0.58 mmol) in EtOAc (25 mL), 10 % Pd/C (62 mg, 0.058 mmol) was added and stirred at 25 °C under H_2 atmosphere from a balloon for 18 h. Progress of the reaction was monitored by TLC using EtOAc as eluent. Upon completion of the reaction, it was filtered over celite 585 then the filter cake was washed with EtOAc (50 mL). Concentration of the combined filtrate *in vacuo* gave a pale peach colored powder **8** (145 mg, 84 %); Mp. 179-181 °C; ^{1}H NMR (400 MHz, MeOH-*d*4) δ 7.02 (d, 2H, *J* = 8.4 Hz), 6.76-6.71 (m, 2H), 6.70-6.65 (m, 3H), 5.90 (s, 2H), 3.35, (t, 2H, *J* = 7.0 Hz), and 2.71 (t, 2H, *J* = 7.0 Hz); ^{13}C{^{1}H} NMR (100

MHz, MeOH-d_4) δ 159.2, 149.2, 147.5, 144.7, 134.5, 133.3, 123.8, 122.8, 117.1, 110.1, 109.1, 102.1, 42.6, and 37.1; HRMS calcd for $C_{16}H_{17}N_3O_3$ $[M + H]^+$: 300.1348, found 300.1344.

1-(4-Aminophenyl)-3-(3,4-dihydroxyphenethyl)urea (1f)

To a solution of **8** (100 mg, 0.33 mmol) in CH_2Cl_2 (10 mL), a solution of BBr_3 in CH_2Cl_2 (1 mL, 1M) was added at 0 °C. The reaction mixture was stirred for 2 h and then allowed to attain room temperature. TLC analysis using 10 % MeOH in $CHCl_3$ as eluent showed the disappearance of the starting material. Upon completion of the reaction, excess BBr_3 in the reaction mixture was quenched by slow drop-wise addition of MeOH until the evolution of gases ceased. Solvents were removed *in vacuo* and the crude product obtained was purified by column chromatography over Si gel using 0-10 % MeOH in $CHCl_3$ as eluent to afford pure compound **1f** as a pale green solid (75 mg, 78 %); Mp. 152 °C (decomposed); ^{1}H NMR (400 MHz, MeOH-d_4) δ 7.53 (d, 2H, J = 9.8 Hz), 7.27 (d, 2H, J = 9.8 Hz), 6.75-6.68 (m, 2H), 6.56 (dd, 1H, J_1 = 2.0 Hz, J_2 = 8.0 Hz), 3.38, (t, 2H, J = 6.8 Hz), and 2.68 (t, 2H, J = 7.0 Hz); $^{13}C\{^1H\}$ NMR (100 MHz, MeOH-d_4) δ 157.8, 146.3, 144.8, 142.2, 132.1, 125.2, 124.4, 121.1, 120.7, 116.9, 116.5, 42.5, and 36.6; HRMS calcd for $C_{15}H_{17}N_3O_3$ $[M + H]^+$: 288.1348, found 288.1346.

4-(3-(2-(Benzo[d][1,3]dioxol-5-yl)ethyl)ureido)benzoic acid (9)

To a solution of **5d** (106 mg, 0.31 mmol) in MeOH (15 mL), 6N NaOH (15 mL) was added, and the reaction mixture was stirred at 25 °C for 16 h. Progress of the reaction was monitored by TLC analysis using EtOAc as eluent. Upon completion of the reaction, the reaction mixture was acidified with 1N HCl and the solid product formed was filtered, washed with water (50 mL), and hexane (50 mL) to afford compound **9** as white flaky solid (85 mg, 84 %); Mp. 255 °C (decomposed); ^{1}H NMR (400 MHz, acetone-d_6) δ 8.46 (s, 1H), 7.89 (d, 2H, J = 8.8 Hz), 7.59 (d, 2H, J = 8.8 Hz), 6.80-6.69 (m, 3H), 6.00 (t, 1H, J = 5.0 Hz), 5.95 (s, 2H), 3.42 (dt, 2H, J_1 = 6.8 Hz, J_2 = 5.0 Hz), and 2.75 (t, 2H, J = 6.8 Hz); $^{13}C\{^1H\}$ NMR (100 MHz, acetone-d_6) δ 167.5, 155.7, 148.6, 146.9, 146.1, 134.3, 131.5, 123.9, 122.6, 117.7, 109.9, 108.9, 101.7, 42.2, and 36.7; HRMS calcd for $C_{17}H_{16}N_2O_5$ $[M + Na]^+$: 351.0957, found 351.0956.

4-(3-(3,4-Dihydroxyphenethyl)ureido)benzoic acid (1g)

Compound **9** (50 mg, 0.15 mmol) was added to a solution of BBr_3 in CH_2Cl_2 (1 mL, 1 M) at 0 °C. The reaction mixture was stirred at 0 °C for 2 h and then allowed to attain room temperature. TLC analysis using 5 % MeOH in $CHCl_3$ as eluent showed complete disappearance of the starting material. Excess BBr_3 in the reaction mixture was quenched by drop-wise addition of MeOH and solvents were removed *in vacuo* to obtain crude product, which was purified by column chromatography over Si gel using 0-5 % MeOH in $CHCl_3$ as eluent to afford pure compound **1g** as an off-white solid (38 mg, 82 %); Mp. 247 ºC (decomposed); ^{1}H NMR (300 MHz, acetone-d_6) δ 8.33 (bs, 1H), 7.91 (d, 2H, J = 8.6 Hz), 7.59 (d, 2H, J = 8.6 Hz), 6.78-6.68 (m, 2H), 6.56 (d, 1H, J = 8.0 Hz), 5.84 (t, 1H, J = 5.8 Hz), 3.41 (dt, 2H, J_1 = 6.8 Hz, J_2 = 5.8 Hz), and 2.67 (t, 2H, J = 6.8 Hz); $^{13}C\{^1H\}$ NMR (75 MHz, acetone-d_6) δ 167.5, 155.6, 146.1, 145.8, 144.3, 132.0, 123.9, 125.7, 120.9, 117.7, 116.6, 116.1, 42.1, and 36.3: HRMS calcd for $C_{16}H_{16}N_2O_5$ $[M +H]^+$: 317.1137, found 317.1142.

2-(Benzo[d][1,3]dioxol-5-yl)-N-(2-(benzo[d][1,3]dioxol-5-yl)ethyl)acetamide (11)

To a solution of 1,3-benzodioxole-5-acetic acid **10** (1 g, 5.55 mmol) in CH_2Cl_2 (20 mL), EDC (1.6 g, 8.33 mmol) and DMAP (6.8 mg, 0.055 mmol) were added, and the reaction mixture was stirred at 25 °C for 15 min. Then 3,4-methylenedioxyphenethylamine **2b** (0.92 g, 5.55 mmol)

was added and the reaction mixture was stirred at 35 °C for 48 h. Upon completion of the reaction as indicated by TLC analysis in 30 % MeOH in $CHCl_3$, the reaction mixture was diluted with CH_2Cl_2 (30 mL), washed with 1N HCl (2 × 30 mL), brine (2 × 30 mL), and dried over Na_2SO_4. Drying agent was filtered off and the filtrate was concentrated *in vacuo* to obtain the crude product, which was purified by column chromatography over Si gel using 10 % MeOH in $CHCl_3$ to afford the pure product **11** as a white powder (1.5 g, 82 %); Mp. 120-121 ºC; ^{1}H NMR (400 MHz, acetone-*d*$_6$) δ 7.05 (s, 1H), 6.79 (s, 1H), 6.76-6.69 (m, 3H), 6.68 (s, 1H), 6.60 (d, 1H, *J* = 8.0 Hz), 5.96, (s, 2H), 5.94 (s, 2H), 3.39-3.31 (m, 4H), and 2.66 (t, 2H, *J* = 7.2 Hz); ^{13}C{^{1}H} NMR (100 MHz, acetone-*d*$_6$) δ 171.0, 148.5 (2C), 147.2, 146.9, 134.2, 130.9, 123.0, 122.5, 110.4, 109.9, 108.8, 108.7, 101.8, 101.7, 43.5, 41.6, and 36.0; HRMS calcd for $C_{18}H_{17}NO_5$ $[M + Na]^+$: 350.1004, found 350.0999.

N-(3,4-Dihydroxyphenethyl)-2-(3,4-dihydroxyphenyl)acetamide (1h)

Prepared using the general deprotection procedure B starting with **11** (229 mg, 0.7 mmol) and 1M BBr_3 in CH_2Cl_2 (1 mL) to afford pure **1h** as a pale-yellow solid (116 mg, 55 %); Mp. 42-44 ºC; ^{1}H NMR (400 MHz, acetone-*d*$_6$) δ 8.37 (bs, 1H), 6.89 (s, 1H), 6.79 (d, 1H, *J* = 8.0 Hz), 6.76-6.72 (m, 2H), 6.62 (d, 1H, *J* = 8.0 Hz), 6.49 (d, 1H, *J* = 8.0 Hz), 3.59 (s, 2H), 3.48 (t, 2H, *J* = 7.2 Hz), and 2.69 (t, 2H, *J* = 7.2 Hz); ^{13}C{^{1}H} NMR (100 MHz, acetone-*d*$_6$) δ 175.3, 145.9, 145.7, 145.3, 144.4, 131.0, 126.1, 121.8, 121.0, 117.5, 116.7, 116.5, 116.2, 43.1, 41.3, and 35.0; HRMS calcd for $C_{16}H_{17}NO_5$ $[M + H]^+$: 304.1185, found 304.1184.

1,3-Bis(2-(benzo[d][1,3]dioxol-5-yl)ethyl)urea (13)

Prepared using the general procedure A starting from 3,4-methylenedioxyphenethylamine **2b** (235 mg, 1.23 mmol) and isocyanate **12** (203 mg, 1.23 mmol) to afford compound **13** as a yellow solid (370 mg, 84 %); Mp. 204-206 ºC; ^{1}H NMR (400 MHz, DMSO-*d*$_6$) δ 6.81 (d, 2H, *J* = 8.0 Hz), 6.77 (s, 2H), 6.63 (d, 2H, *J* = 8.0 Hz), 5.96 (s, 4H), 5.83 (t, 2H, *J* = 5.6 Hz), 3.16, (dt, 4H, J_1 = 7.2 Hz, J_2 = 5.6 Hz), and 2.56 (t, 4H, *J* = 7.2 Hz); ^{13}C{^{1}H} NMR (100 MHz, DMSO-*d*$_6$) δ 157.8, 147.1, 145.4, 133.6, 121.5, 109.0, 108.1, 100.6, 41.0, and 35.8; HRMS calcd for $C_{19}H_{20}N_2O_5$ $[M + Na]^+$: 379.1270, found 379.1269.

1,3-Bis(3,4-dihydroxyphenethyl)urea (1i)

Prepared using the general deprotection procedure B starting with compound **13** (200 mg, 0.56 mmol) and 1M BBr_3 in CH_2Cl_2 (1 mL) to afford compound **1i** as yellow solid (146 mg, 78 %); Mp. 63-65 ºC; ^{1}H NMR (400 MHz, acetone-*d*$_6$) δ 6.76-6.71 (m, 4H), 6.55-6.45 (m, 4H), 3.35 (t, 4H, *J* = 7.2 Hz), and 2.62 (t, 4H, *J* = 7.2 Hz); ^{13}C{^{1}H} NMR (100 MHz, acetone-*d*$_6$) δ 159.7, 145.8, 144.3, 131.8, 120.9, 116.7, 116.1, 42.8, and 36.4; HRMS calcd for $C_{17}H_{20}N_2O_5$ $[M + H]^+$: 333.1450, found 333.1449.

1-(Benzo[d][1,3]dioxol-5-yl)-3-(2-(benzo[d][1,3]dioxol-5-yl)ethyl)thiourea (15)

A solution of 3,4-methylenedioxyphenethylamine **2b** (1.1 g, 6.15 mmol) and 3,4-methylenedioxyphenylisothiocyanate **14** (1.02 g, 6.15 mmol) in CH_2Cl_2 (30 mL) was stirred at 25 °C for 4 h. TLC analysis using 100 % EtOAc as eluent revealed that the reaction was complete. The precipitated solid was filtered and washed with water (50 mL) and hexanes (50 mL) to afford pure product **15** as a brown solid (2.11 g, 100 %); Mp. 125-127 ºC; ^{1}H NMR (400 MHz, $CDCl_3$) δ 8.13 (bs, 1H), 6.76-6.66 (m, 2H), 6.61 (s, 1H), 6.57-6.52 (m, 3H), 6.00 (s, 2H), 5.92 (s, 2H), 5.87 (bs, 1H), 3.84-3.75 (m, 2H), and 2.80 (t, 2H, *J* = 6.8 Hz); ^{13}C{^{1}H} NMR (100 MHz, $CDCl_3$) δ

180.8, 148.6, 147.8, 147.0, 146.3, 132.1, 129.3, 121.7, 119.7, 109.0, 108.8, 108.3, 107.3, 101.9, 101.0, 46.2, and 34.5; HRMS calcd for $C_{17}H_{16}N_2O_4S$ $[M + H]^+$: 345.0909, found 345.0905.

1-(3,4-Dihydroxyphenethyl)-3-(3,4-dihydroxyphenyl)thiourea (1j)

Prepared using the general deprotection procedure B starting with **15** (200 mg, 0.58 mmol) and 1M BBr_3 in CH_2Cl_2 (2 mL) to afford **1j** as a yellow solid (72 mg, 39 %); Mp. 105-107 ºC; ^1H NMR (400 MHz, acetone-d_6) δ 8.50 (bs, 1H), 8.24 (bs, 1H), 8.08 (bs, 1H), 7.85 (bs, 1H), 7.75 (bs, 1H), 6.84 (bs, 1H), 6.79-6.71 (m, 4H), 6.52 (d, 2H, J = 8.0 Hz), 3.70-3.77, (m, 2H), and 2.75 (t, 2H, J = 7.2 Hz); $^{13}C\{^1H\}$ NMR (100 MHz, acetone-d_6) δ 182.2, 146.4, 145.8, 144.6, 144.4, 131.9, 130.4, 120.9, 118.1, 116.6, 116.4, 116.1, 114.1, 47.3, and 35.2; HRMS calcd for $C_{15}H_{16}N_2O_4S$ $[M + Na]^+$: 343.0728, found 343.0725.

8. Rodent model studies with compounds SN195, 1b, and 1j

The anticariogenic effects of the most active analogs **1b** and **1j** along with the original lead **SN195** were evaluated using a well-established gnotobiotic rat caries model. This model was generated by co-cultivating cultures of *C. albicans* SC5314 and *S. mutans* UA159 to induce caries with previously reported protocols [3, 4]. This study was divided into six experimental groups of five rats each, except for the uninfected group, which included four rats.

(i) Infected (3 females + 2 males).
(ii) SN195 treated (4 females + 1 male),
(iii) 1b treated (3 females + 2 males),
(iv) 1j treated (3 females + 2 males),
(v) NaF treated (3 females + 2 males) and
(vi) Uninfected (3 females + 1 male).

Fisher 344 rats (n = 29) were fed with Diet 305. All groups were provided sterile 5% sucrose water ad libitum, with the exception of the fluoride-treated group, which received 250 ppm fluoride in the water. All rats in the experimental groups and control groups except the uninfected group were colonized with *S. mutans* UA159 twice daily for four days then *C. albicans* SC5314 twice daily for two days. After 11 days, treatment groups began receiving administration of compounds (100 μM) or fluoride (250 ppm) *ad libitum* for a 4-week period. Rats in treatment groups were given 100 μM compounds via toothbrushing twice daily for 20 seconds. The rats were 64-66 days old by the end of the trial. At the end of the study, the animals were euthanized, their mandibles excised for analysis of carious lesions. The excised rat mandibles were subjected to microbiological analysis to assess CFUs of *S. mutans* and *C. albicans* aiming to determine their respective colonization. Data was analyzed as the mean with its respective standard error of the mean. Statistical analysis was performed using one-way ANOVA and the Brown-Forsythe test was used to check for the presence of outliers. Significance was given by ns= $p>0.05$, *=$p<0.05$, **= $p<0.01$, ****= $p<0.0001$.

9. References

(1) Liu, C.; Worthington, R. J.; Melander, C.; Wu, H. A New Small Molecule Specifically Inhibits the Cariogenic Bacterium *Streptococcus mutans* in Multispecies Biofilms. *Antimicrob. Agents Chemother.* **2011**, *55*, 2679-2687.

(2) Zhang, Q.; Nguyen, T.; McMichael, M.; Velu, S. E.; Zou, J.; Zhou, X.; Wu, H. New Small-Molecule Inhibitors of Dihydrofolate Reductase Inhibit *Streptococcus mutans*. *Int. J. Antimicrob. Agents* **2015**, *46*, 174-182.

(3) Zhang, Q.; Qin, S.; Xu, X.; Zhao, J.; Zhang, H.; Liu, Z.; Chen, W. Inhibitory Effect of Lactobacillus plantarum CCFM8724 towards Streptococcus mutans- and Candida albicans-Induced Caries in Rats. *Oxid. Med. Cell. Longev.* **2020**, *2020*, 4345804.

(4) Falsetta, M. L.; Klein, M. I.; Colonne, P. M.; Scott-Anne, K.; Gregoire, S.; Pai, C. H.; Gonzalez-Begne, M.; Watson, G.; Krysan, D. J.; Bowen, W. H.; et al. Symbiotic relationship between Streptococcus mutans and Candida albicans synergizes virulence of plaque biofilms *in vivo*. *Infect. Immun.* **2014**, *82*, 1968-1981.

10. <u>^{1}H NMR and ^{13}C NMR Spectra of all compounds</u>

_N_-(2_H_-1,3-Benzodioxol-5-yl)-_N'_-[2-(2_H_-1,3-benzodioxol-5-yl) ethyl] urea (4)

^{1}H-NMR (400 MHz, DMSO-d_6)

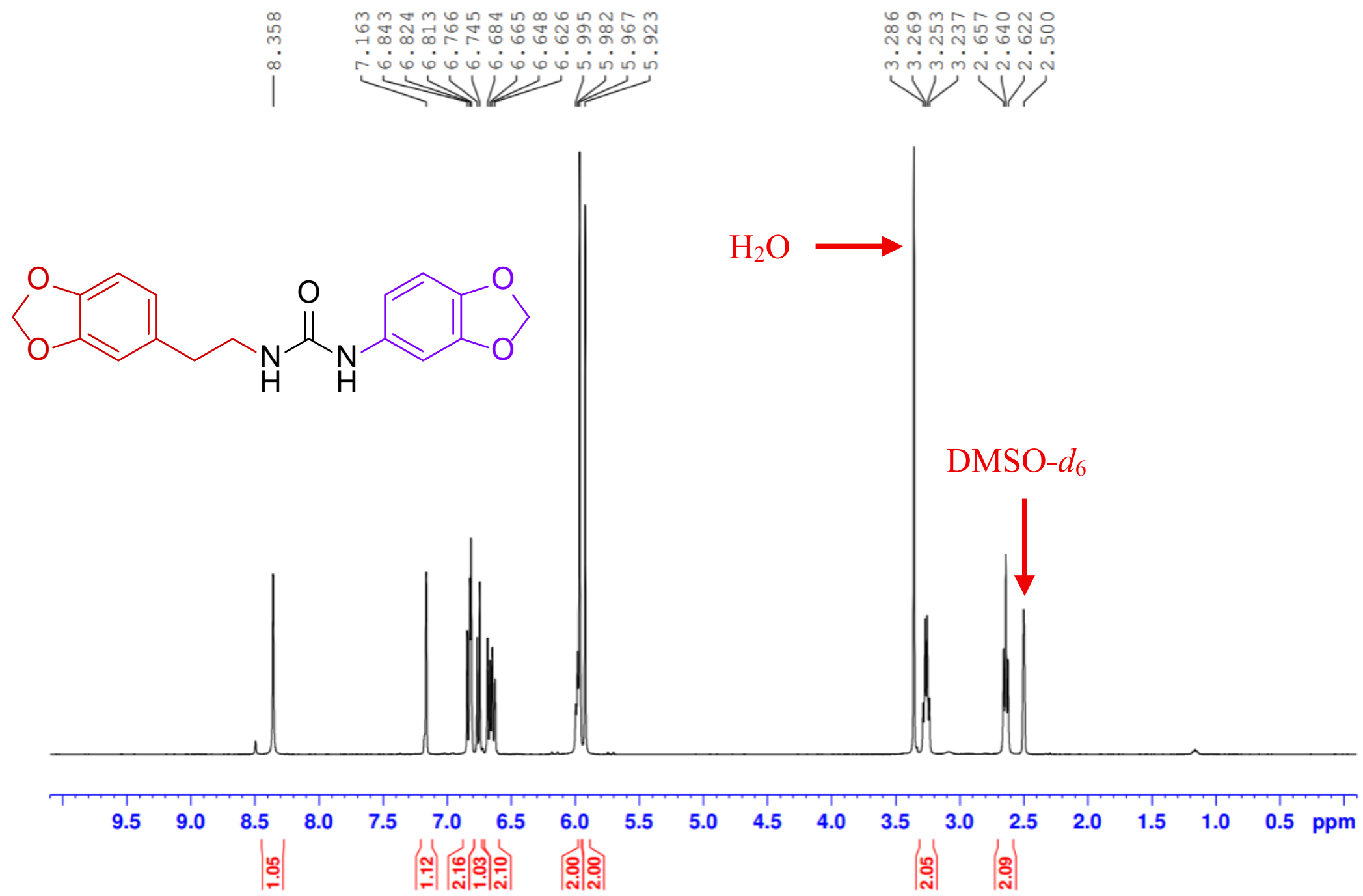

_N_-(2_H_-1,3-Benzodioxol-5-yl)-_N'_-[2-(2_H_-1,3-benzodioxol-5-yl) ethyl] urea (4)

^{13}C-NMR (100 MHz, DMSO-d_6)

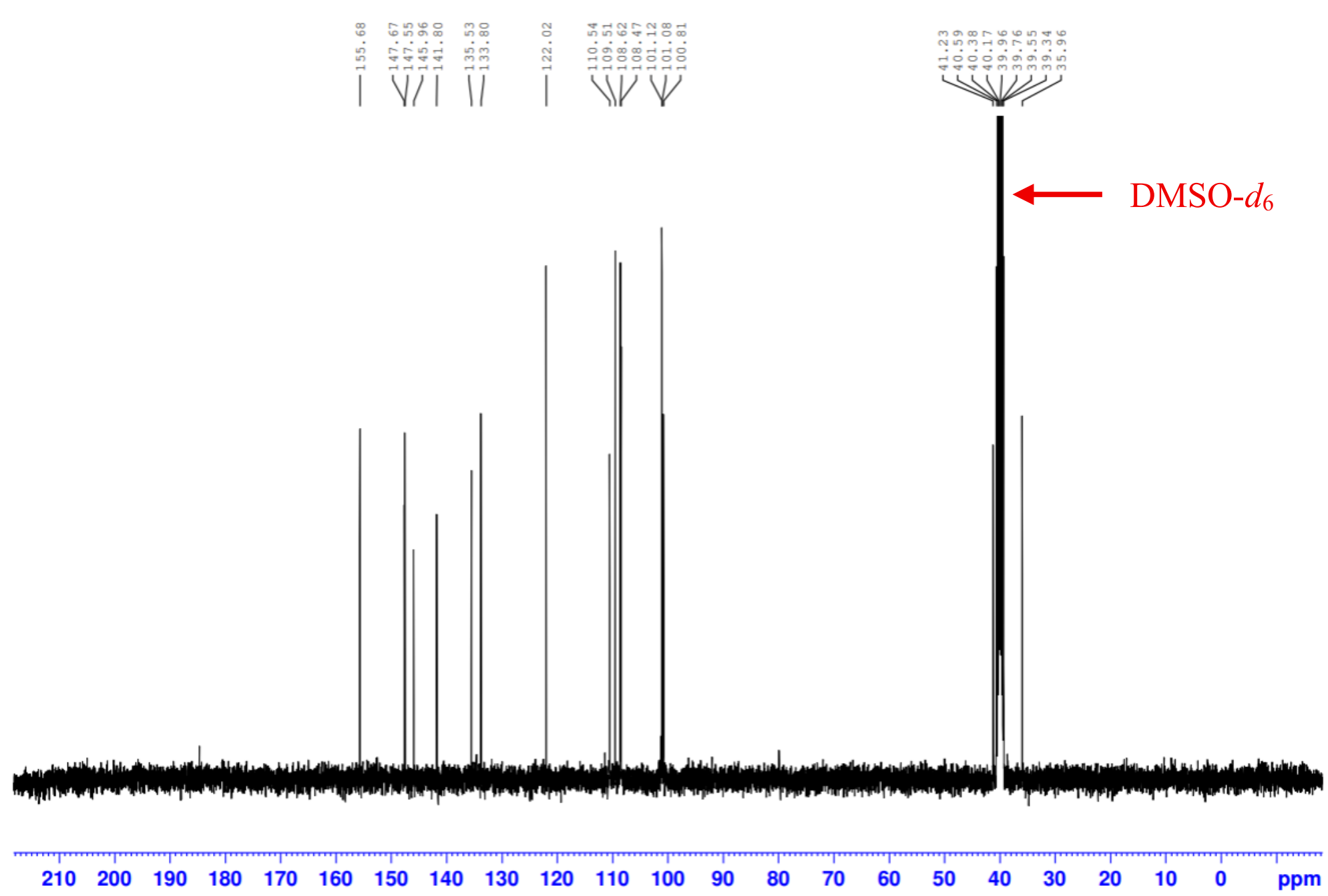

N-(3,4-Dihydroxyphenyl)-N'-[2-(3,4-dihydroxyphenyl) ethyl] urea (SN195)
^{1}H-NMR (400 MHz, MeOH-d_4)

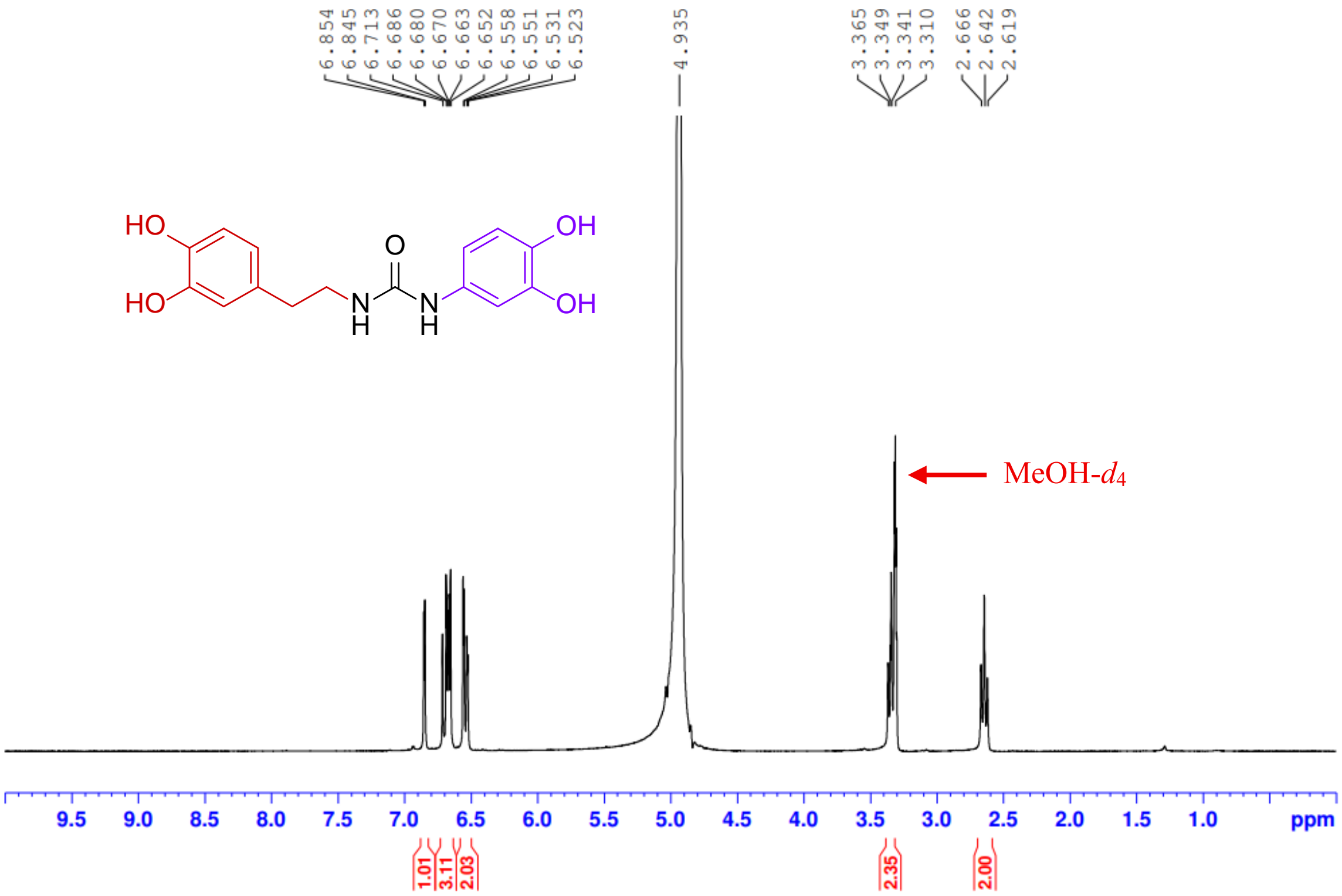

N-(3,4-Dihydroxyphenyl)-N'-[2-(3,4-dihydroxyphenyl) ethyl] urea (SN195)
^{13}C-NMR (100 MHz, MeOH-d_4)

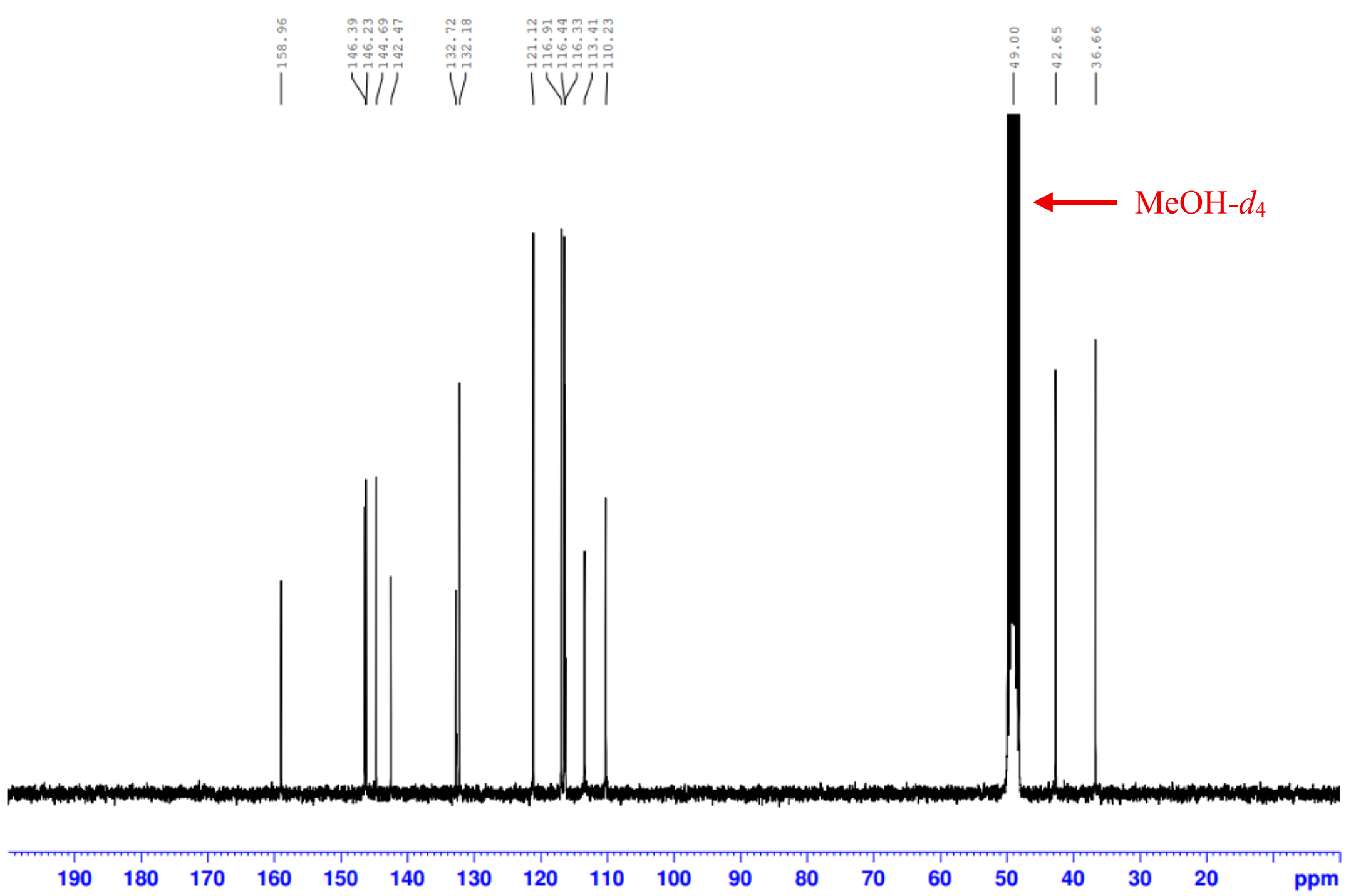

1-(2-(Benzo[d][1,3]dioxol-5-yl)ethyl)-3-(4-methoxyphenyl)urea (5a)

^{1}H-NMR (400 MHz, DMSO-d_6)

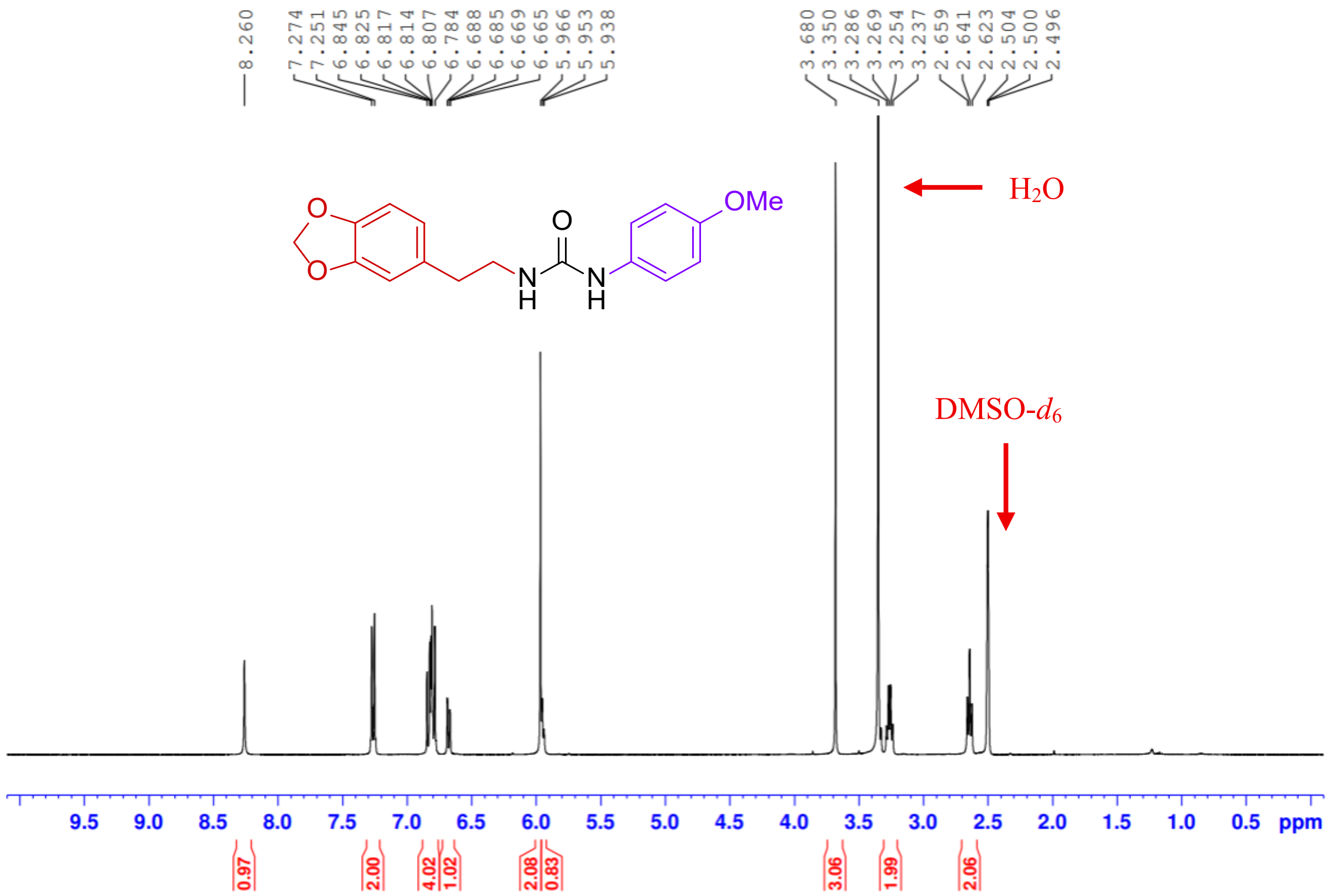

1-(2-(Benzo[d][1,3]dioxol-5-yl)ethyl)-3-(4-methoxyphenyl)urea (5a)

^{13}C-NMR (100 MHz, DMSO-d_6)

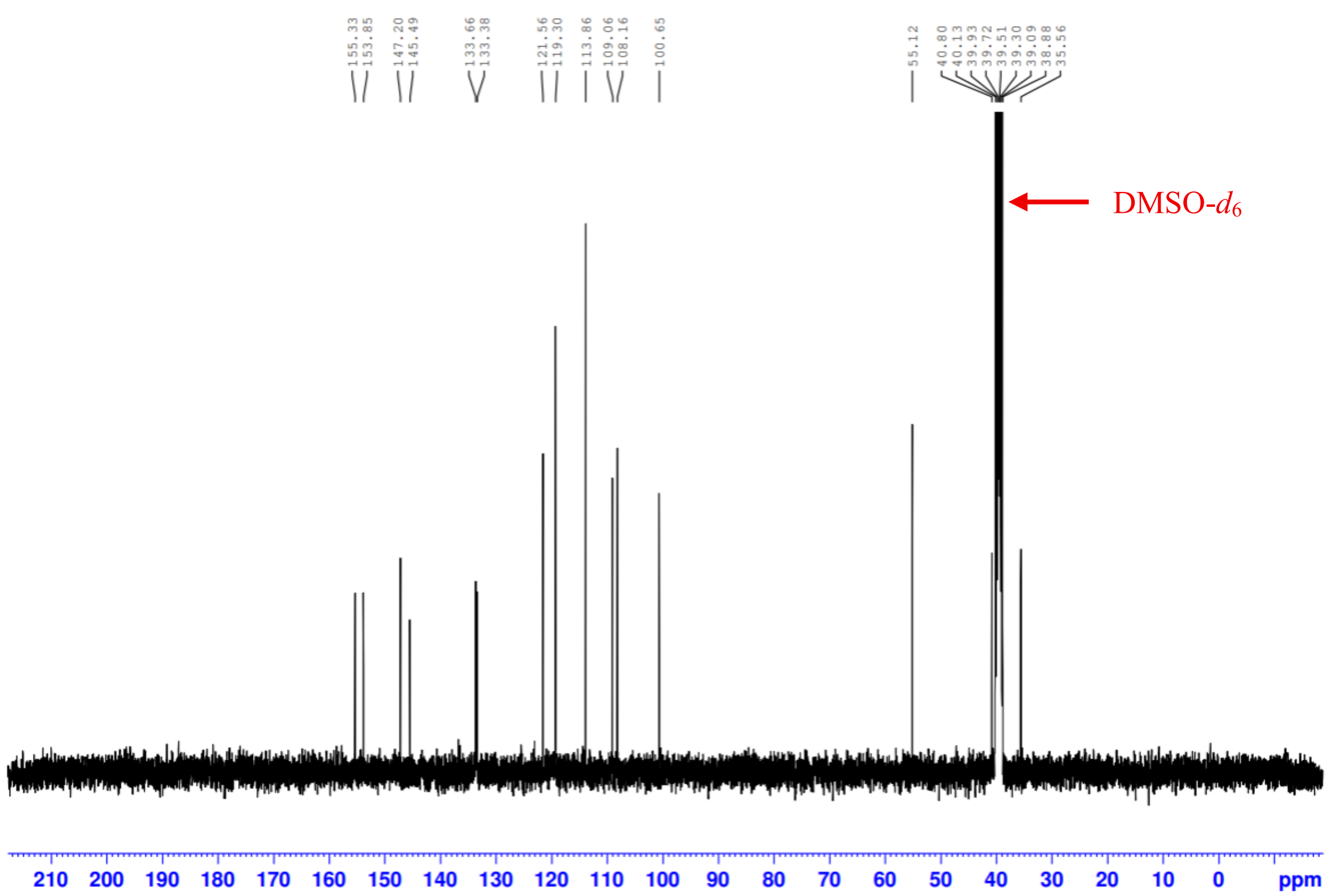

1-(Benzo[d][1,3]dioxol-5-yl)-3-(4-methoxyphenethyl)urea (5b)

^{1}H-NMR (400 MHz, DMSO-d_6)

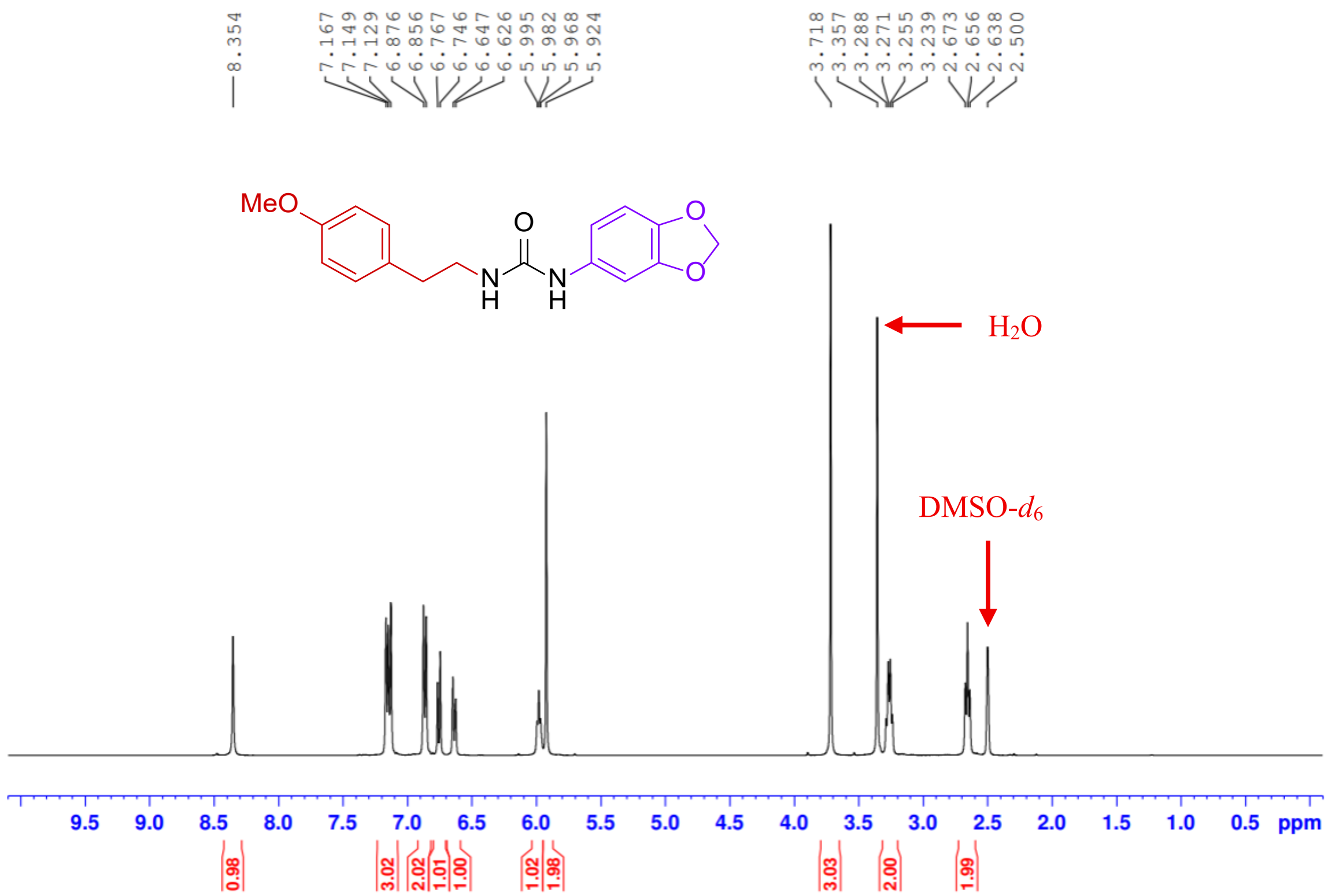

1-(Benzo[d][1,3]dioxol-5-yl)-3-(4-methoxyphenethyl)urea (5b)

^{13}C-NMR (100 MHz, DMSO-d_6)

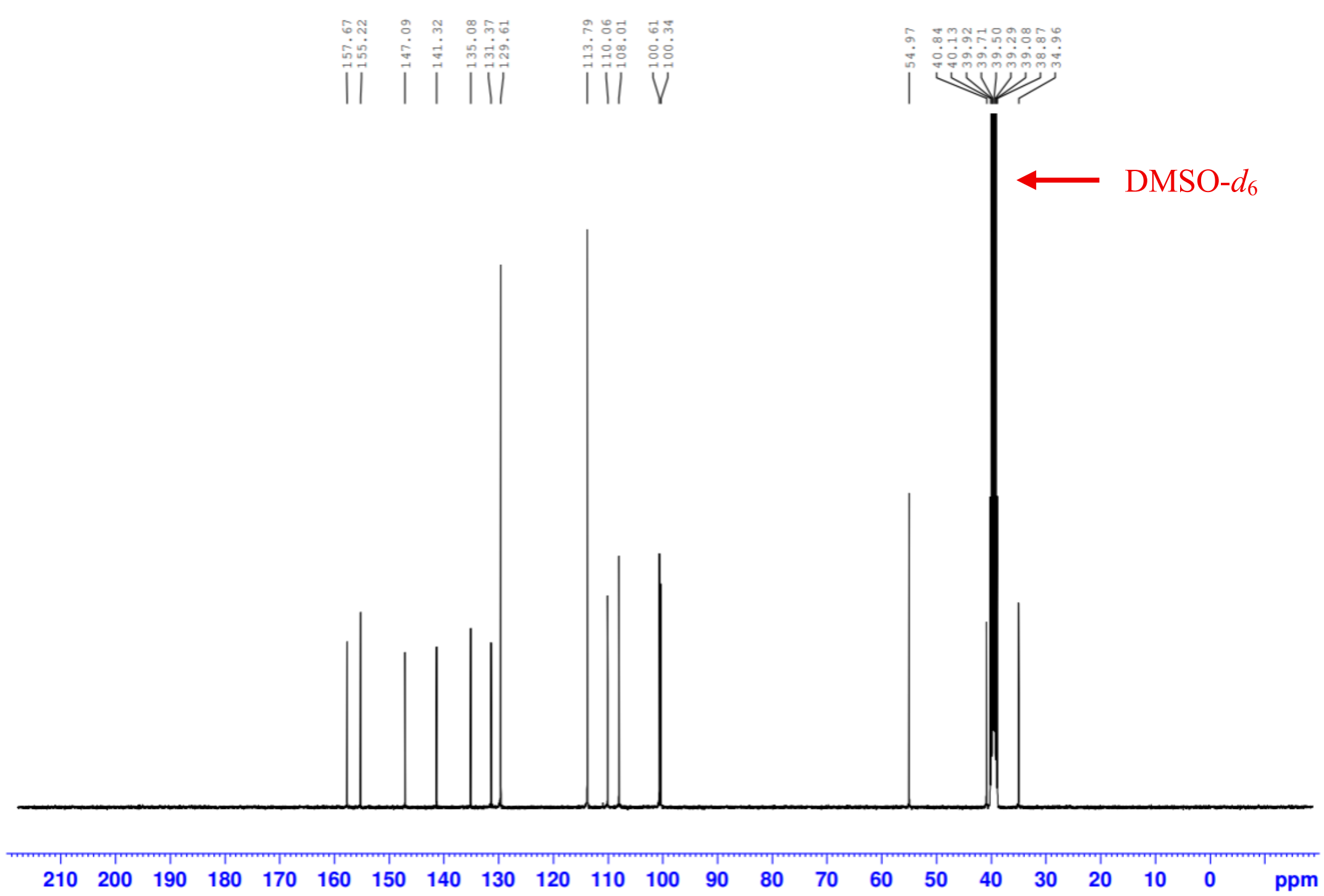

1-(2-(Benzo[d][1,3]dioxol-5-yl)ethyl)-3-(4-nitrophenyl)urea (5c)
^{1}H-NMR (400 MHz, DMSO-d_6)

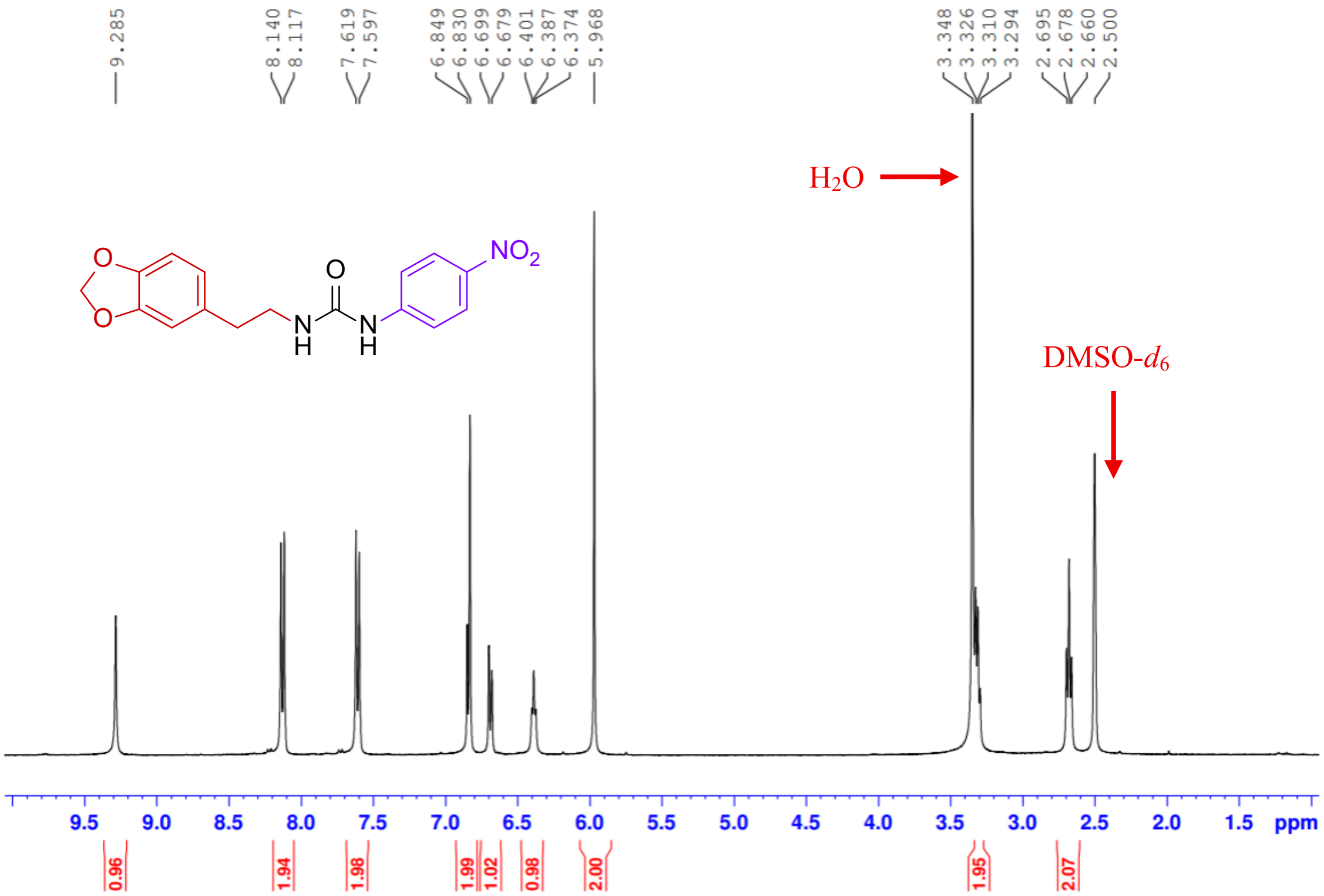

1-(2-(Benzo[d][1,3]dioxol-5-yl)ethyl)-3-(4-nitrophenyl)urea (5c)
^{13}C-NMR (100 MHz, DMSO-d_6)

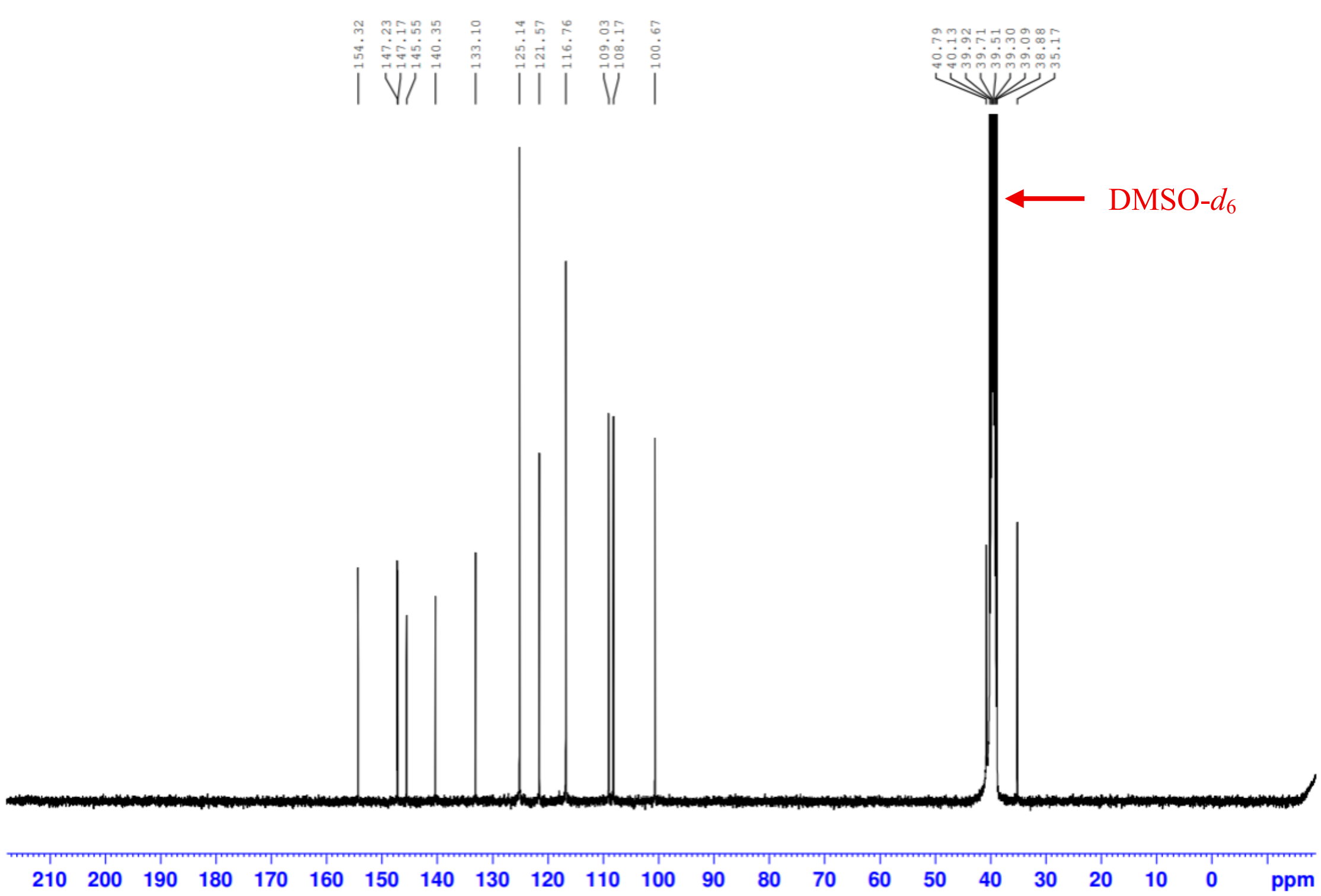

Methyl 4-(3-(2-(benzo[d][1,3]dioxol-5-yl)ethyl)ureido)benzoate (5d)

^{1}H-NMR (400 MHz, DMSO-d_6)

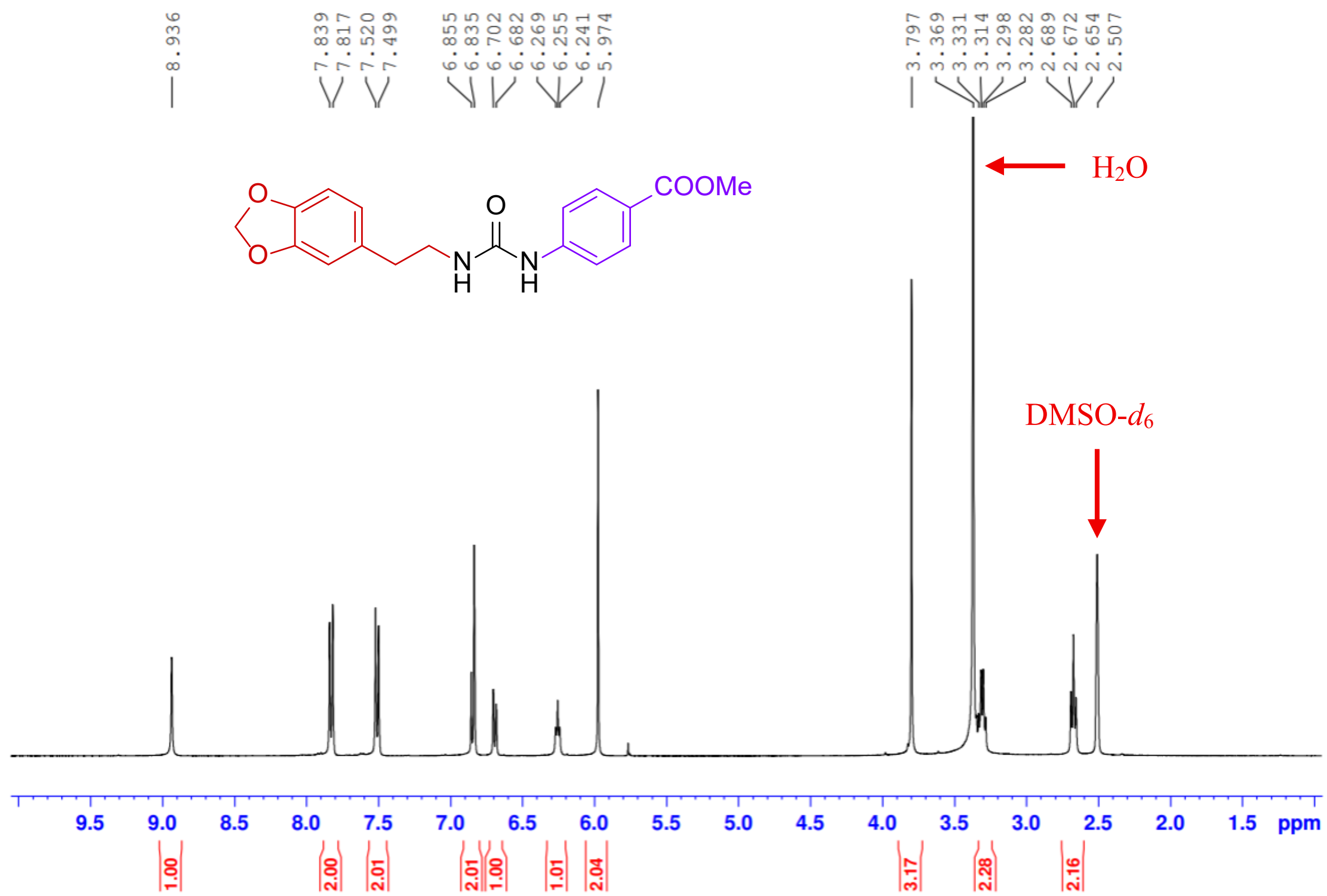

Methyl 4-(3-(2-(benzo[d][1,3]dioxol-5-yl)ethyl)ureido)benzoate (5d)

^{13}C-NMR (100 MHz, acetone-d_6)

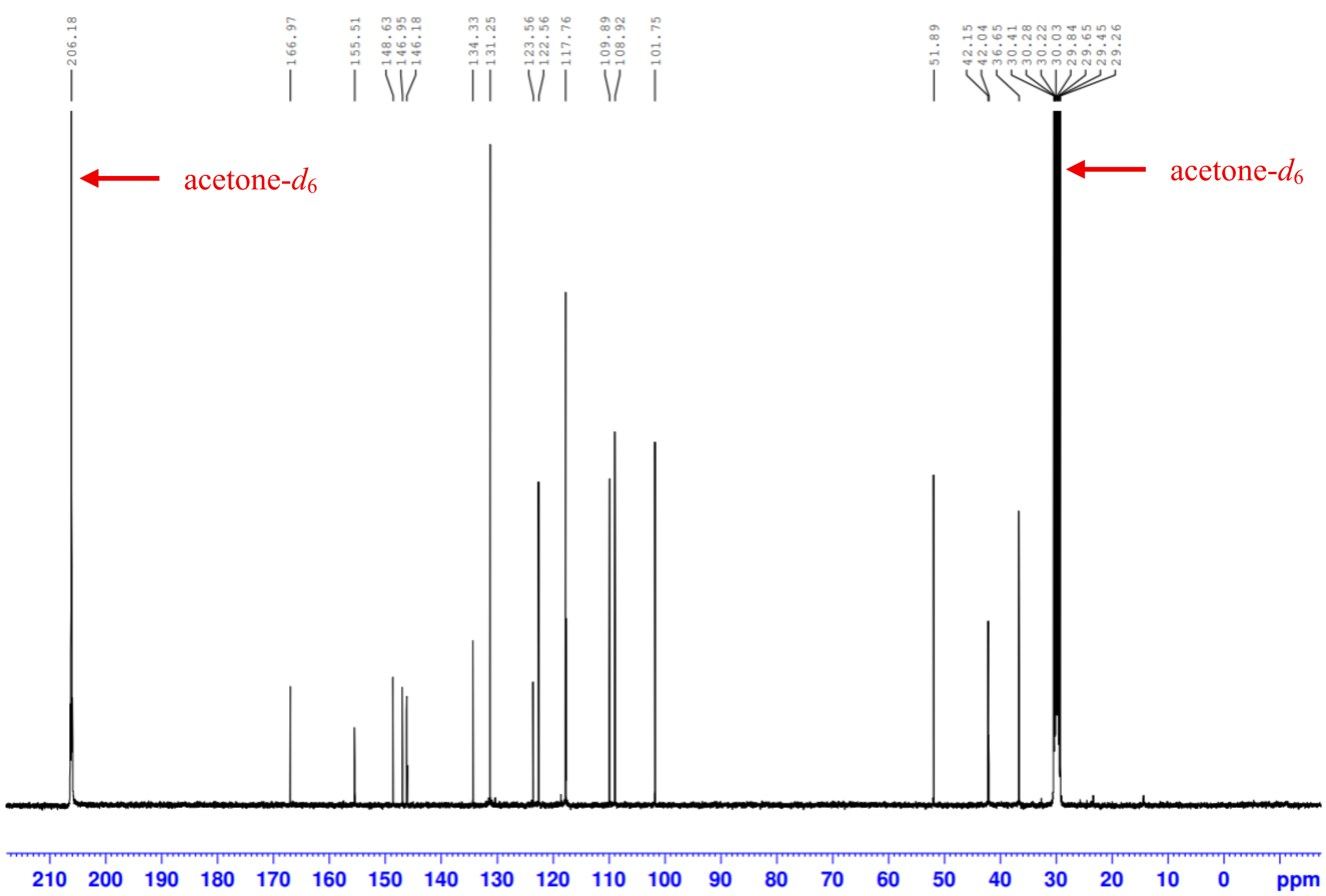

1-(3,4-Dihydroxyphenethyl)-3-(4-hydroxyphenyl)urea (1a)
^{1}H-NMR (400 MHz, acetone-*d*$_6$)

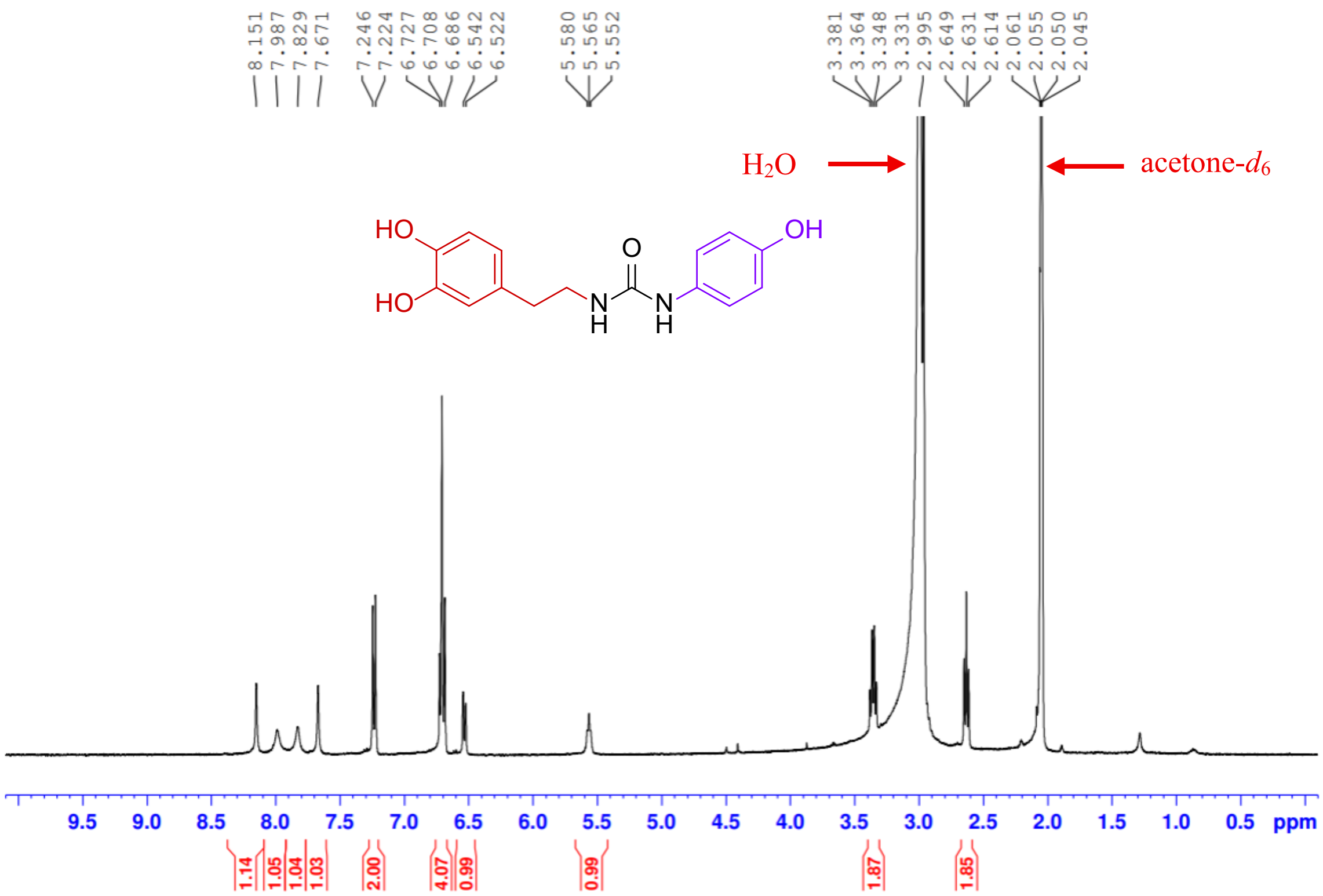

1-(3,4-Dihydroxyphenethyl)-3-(4-hydroxyphenyl)urea (1a)
^{13}C-NMR (100 MHz, acetone-*d*$_6$)

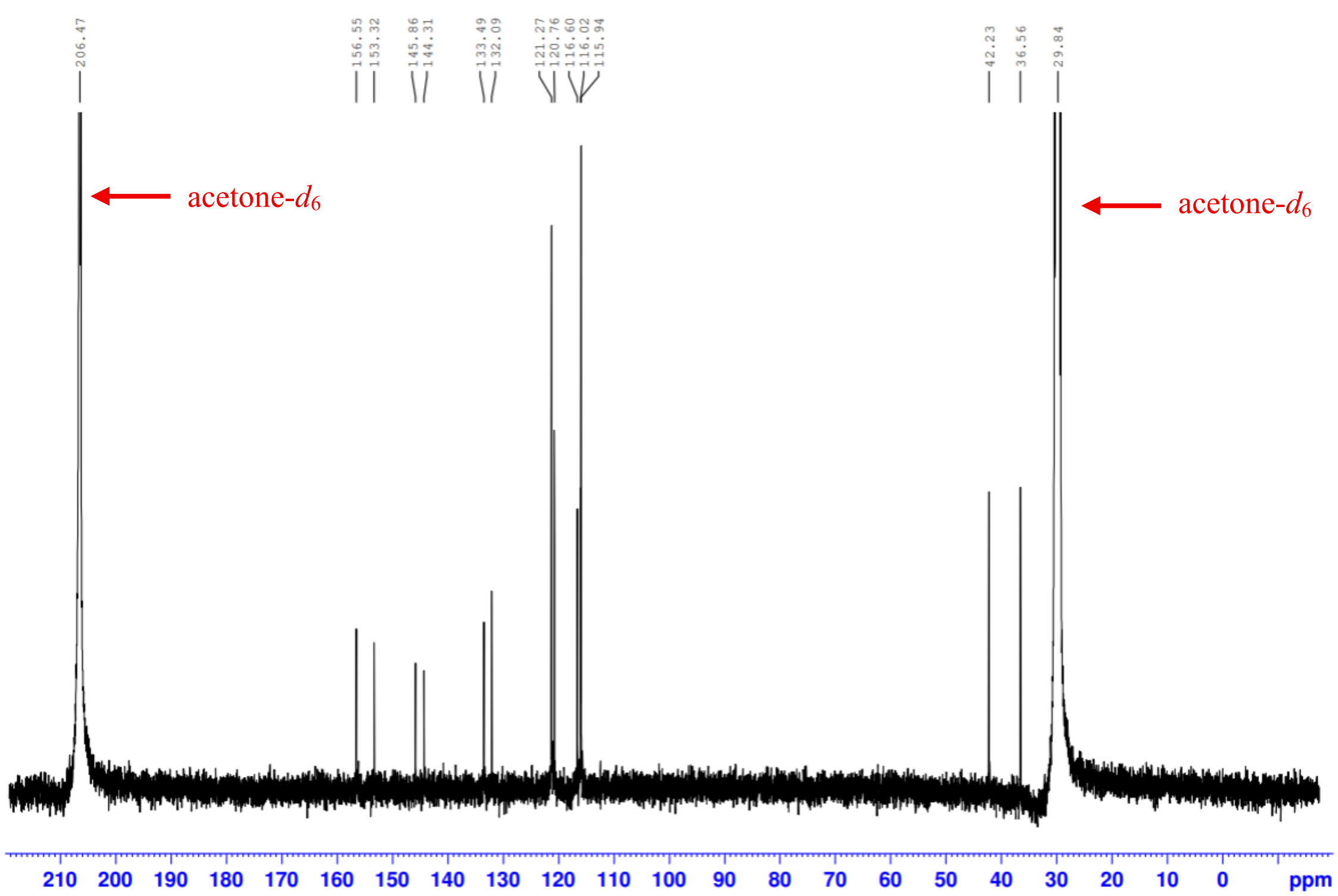

1-(3,4-Dihydroxyphenyl)-3-(4-hydroxyphenethyl)urea (1b)

^{1}H-NMR (300 MHz, MeOH-d_4)

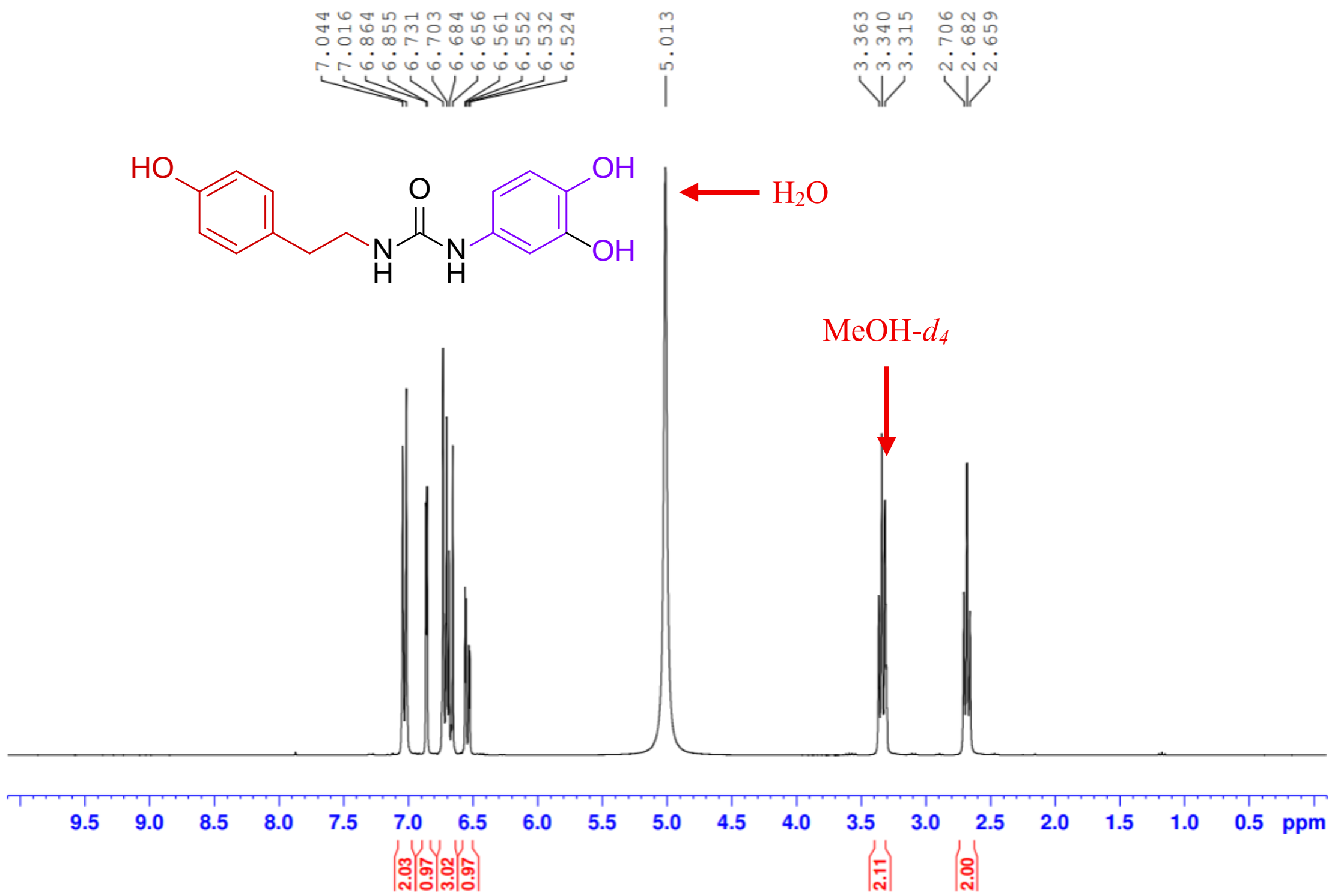

1-(3,4-Dihydroxyphenyl)-3-(4-hydroxyphenethyl)urea (1b)

^{13}C-NMR (75 MHz, MeOH-d_4)

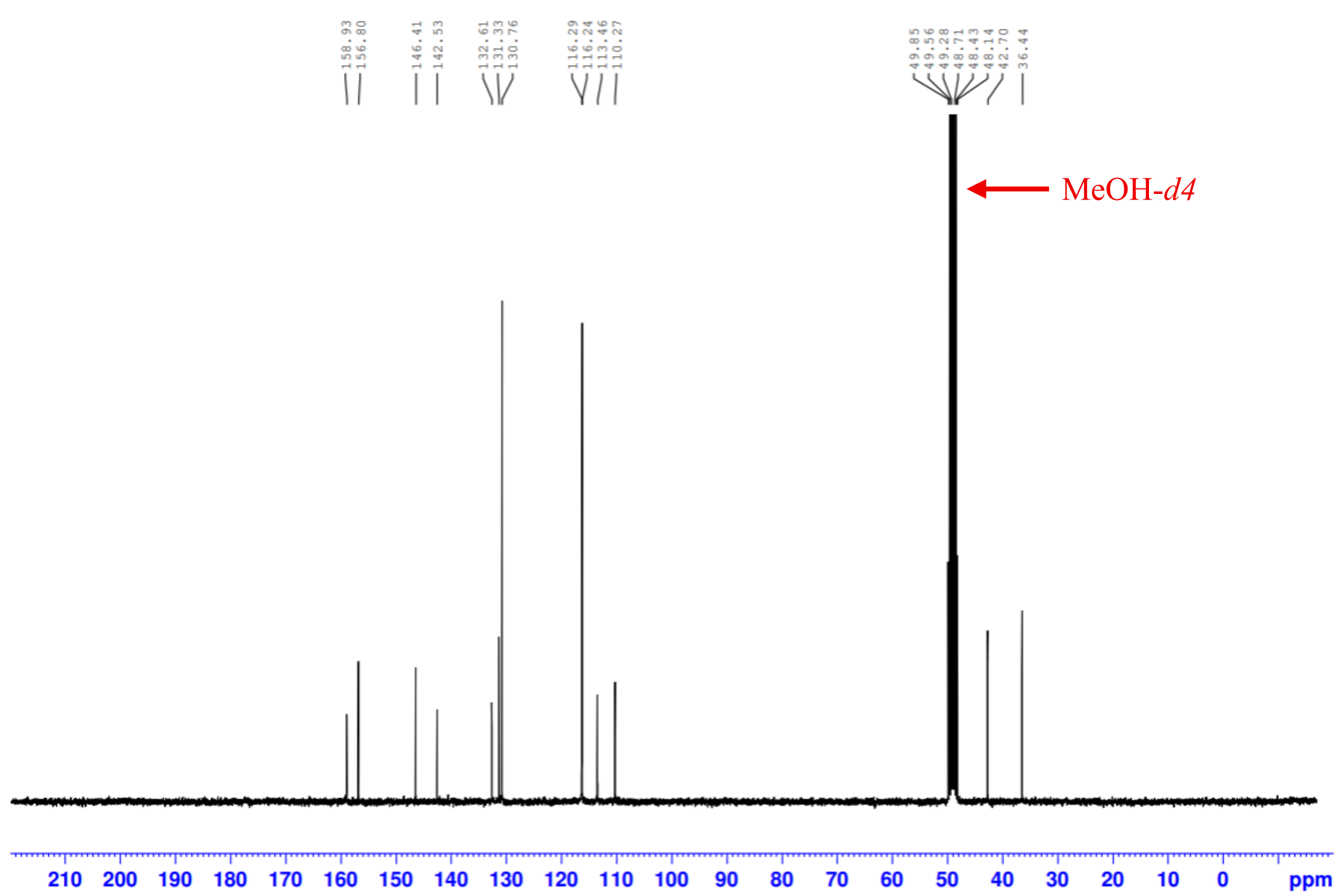

1-(3,4-Dihydroxyphenethyl)-3-(4-nitrophenyl)urea (1c)

^{1}H-NMR (400 MHz, acetone-d_6)

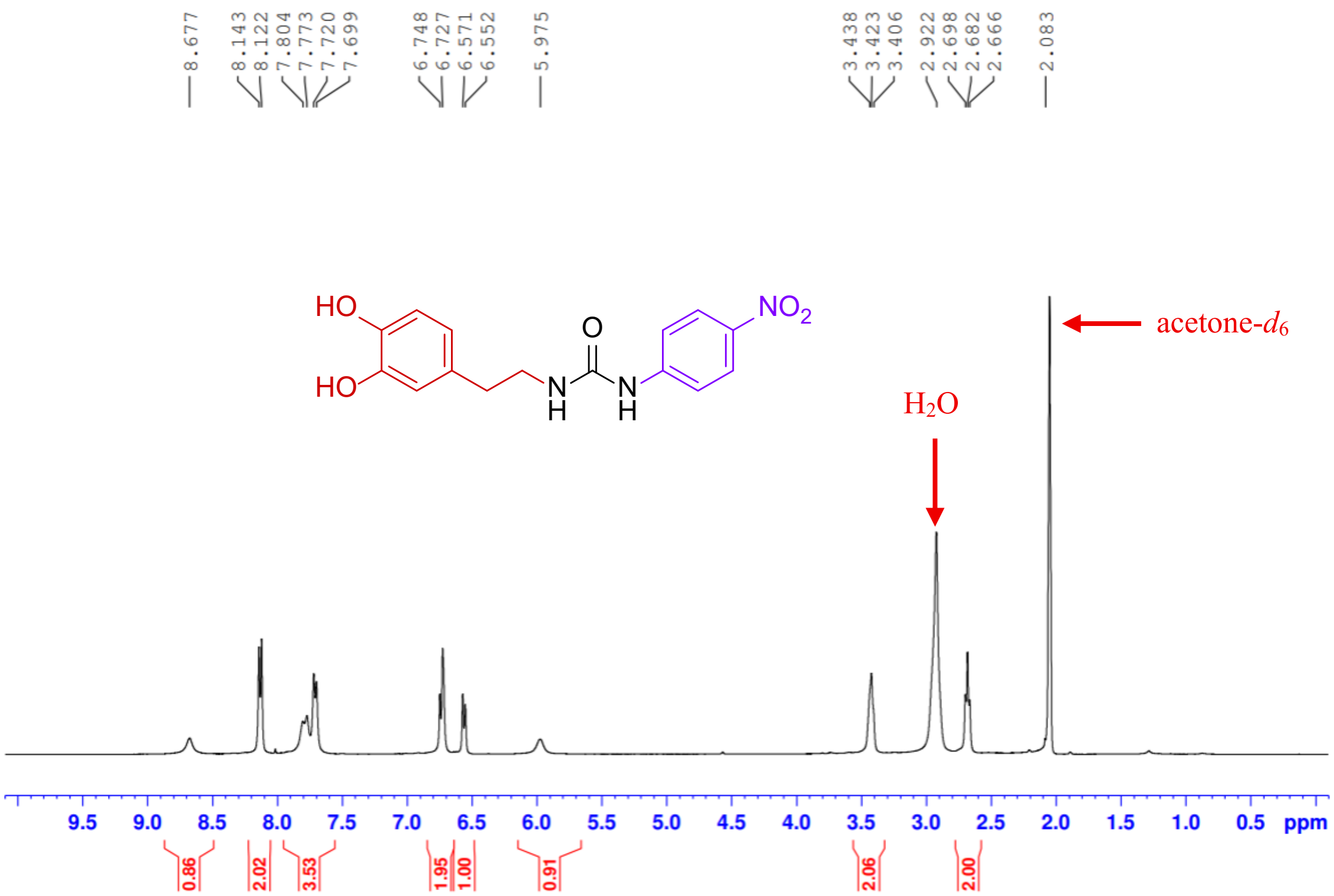

1-(3,4-Dihydroxyphenethyl)-3-(4-nitrophenyl)urea (1c)

^{13}C NMR (100 MHz, acetone-d_6)

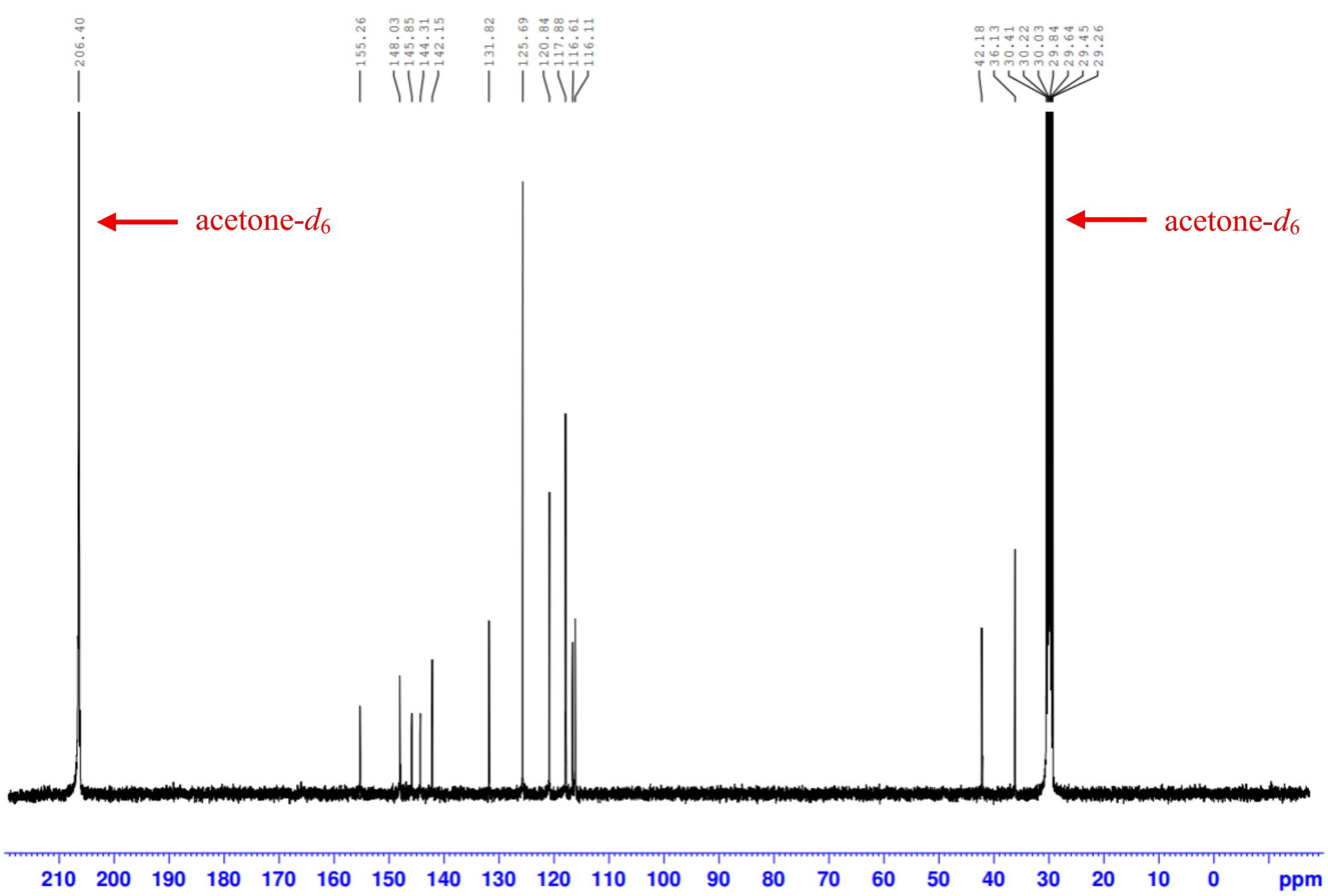

Methyl 4-[3-(3,4-dihydroxyphenethyl)ureido]benzoate (1d)
^{1}H-NMR (400 MHz, acetone-d_6)

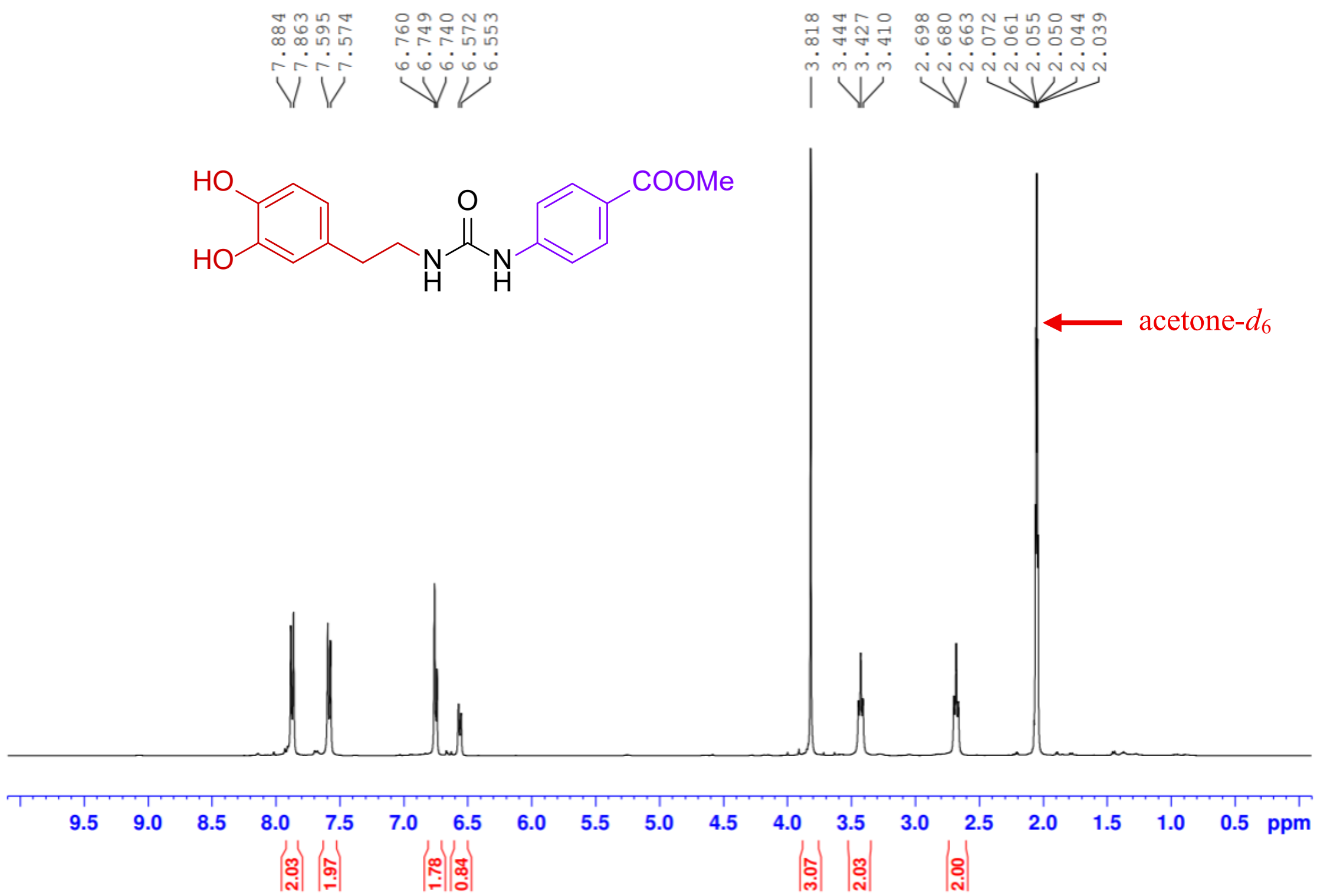

Methyl 4-[3-(3,4-dihydroxyphenethyl)ureido]benzoate (1d)
^{13}C NMR (100 MHz, acetone-d_6)

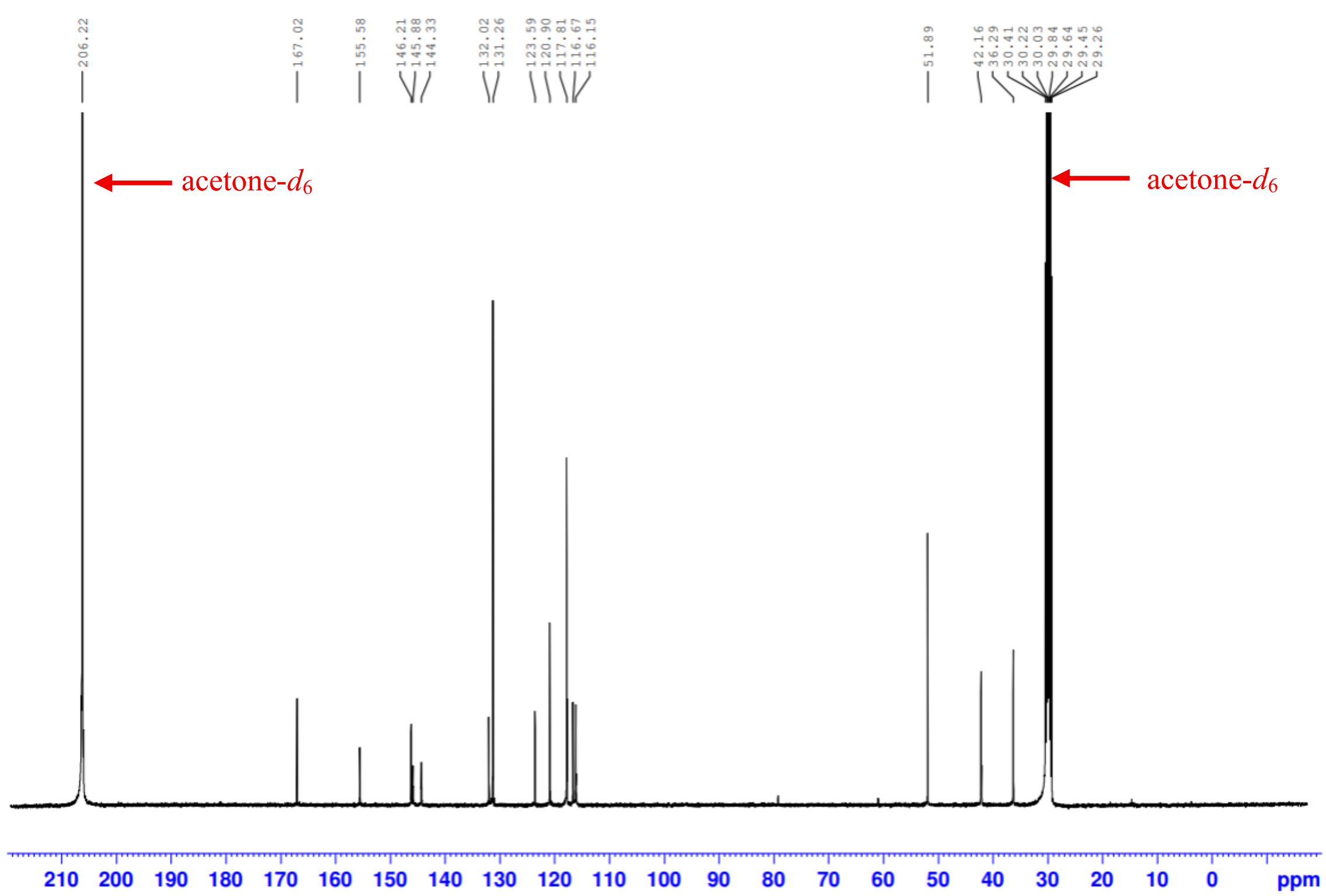

1-(4-Aminophenethyl)-3-(benzo[d][1,3]dioxol-5-yl)urea (7)

^{1}H-NMR (400 MHz, DMSO-d_6)

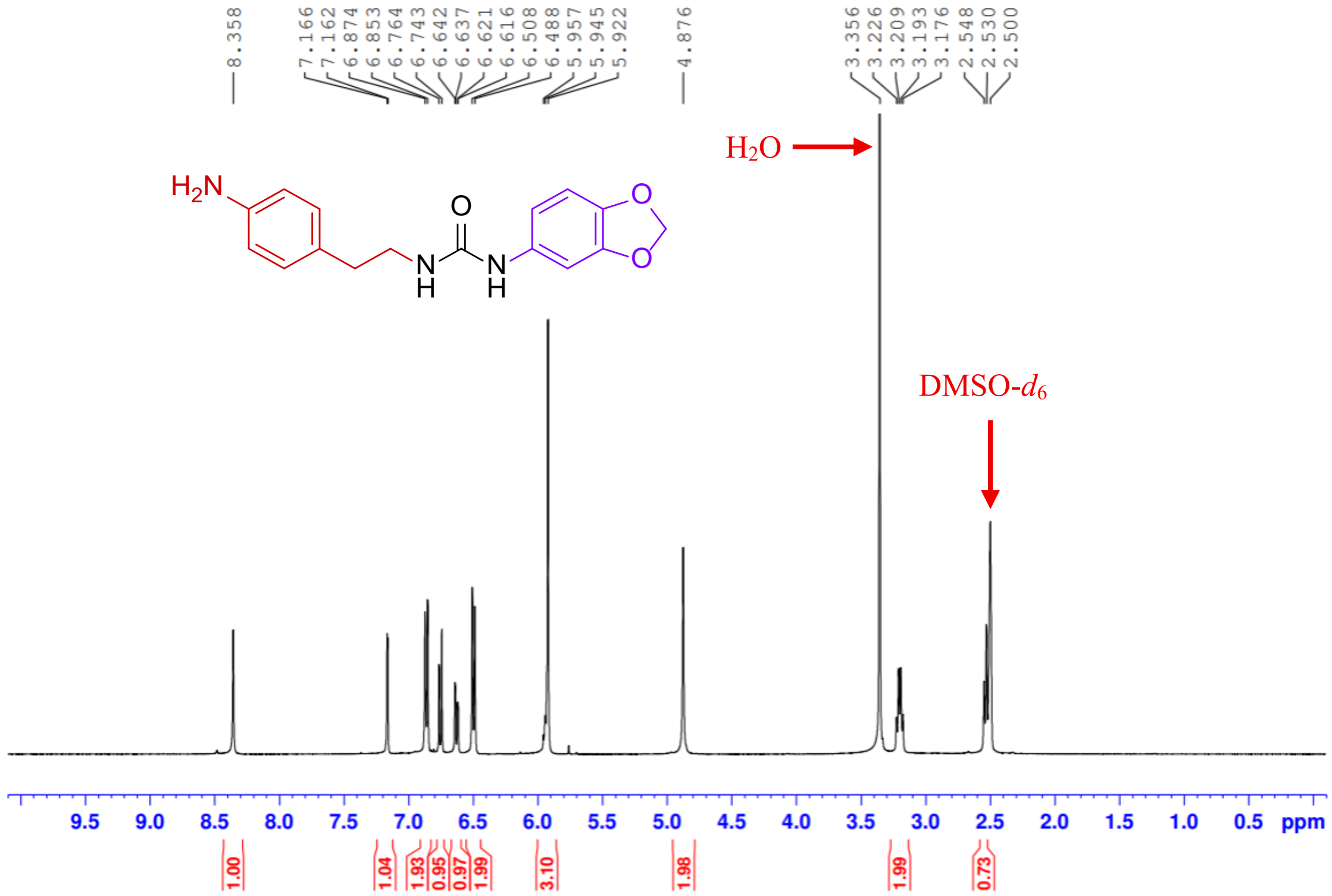

1-(4-Aminophenethyl)-3-(benzo[d][1,3]dioxol-5-yl)urea (7)

^{13}C-NMR (100 MHz, DMSO-d_6)

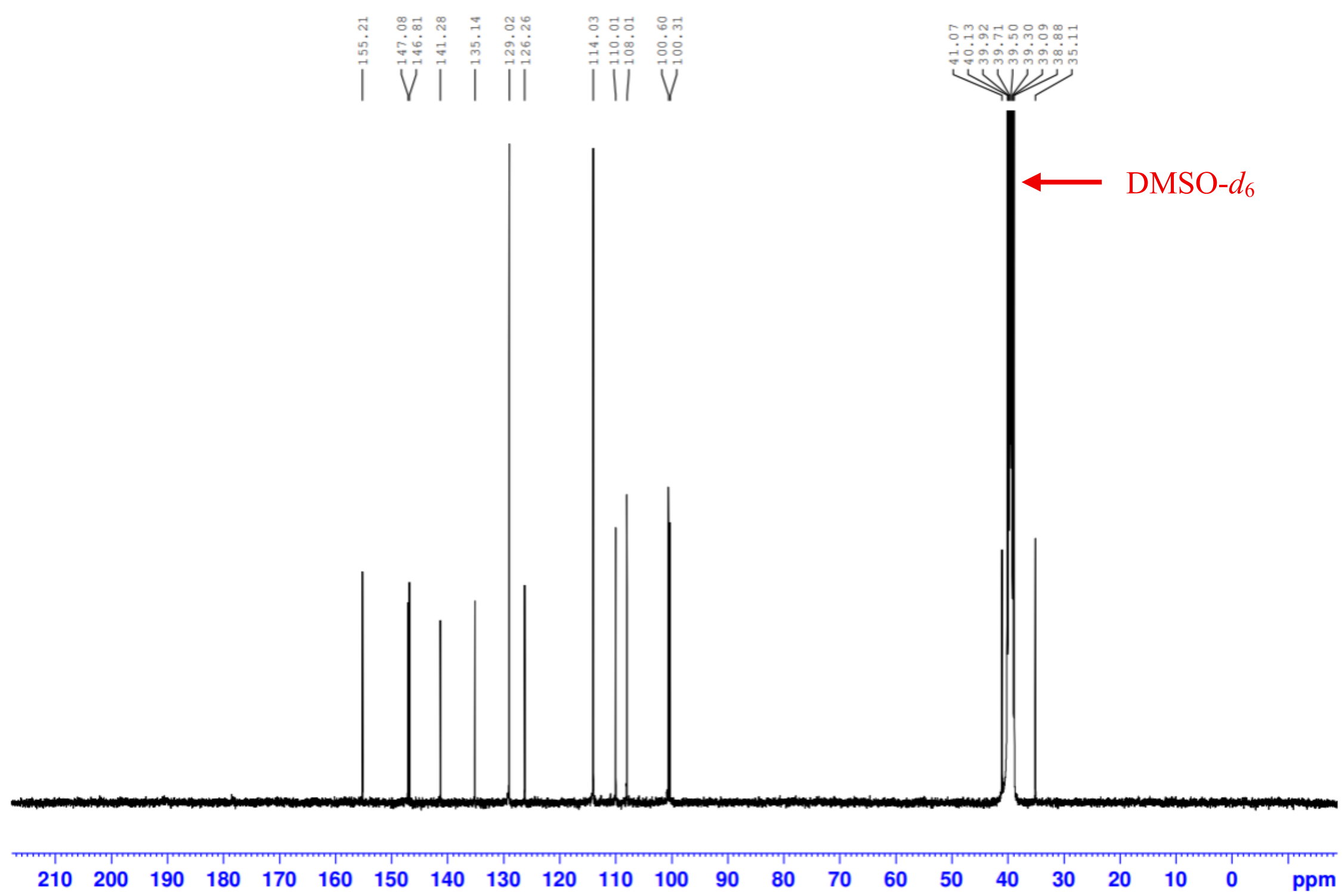

1-(4-Aminophenethyl)-3-(3,4-dihydroxyphenyl)urea (1e)

^{1}H-NMR (400 MHz, MeOH-d_4)

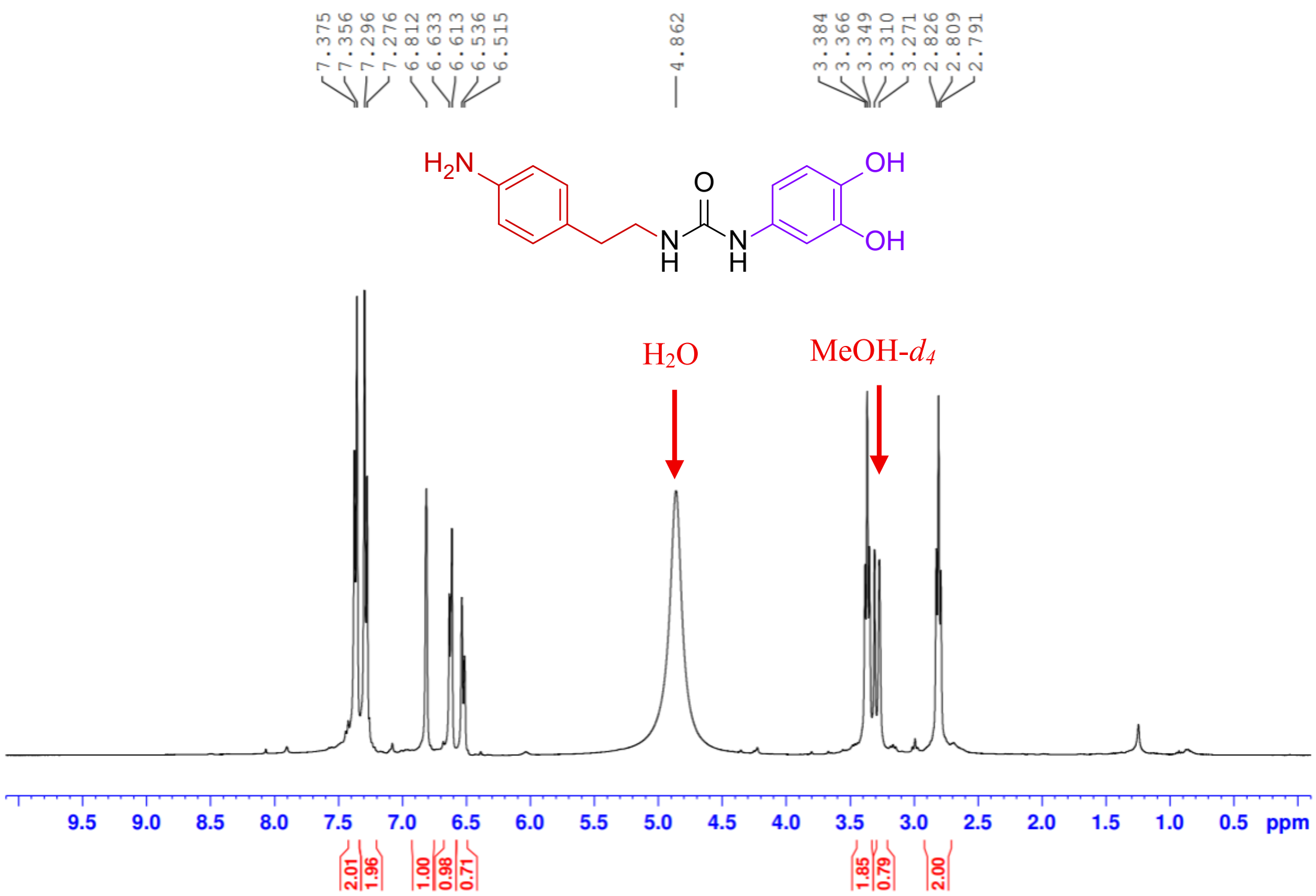

1-(4-Aminophenethyl)-3-(3,4-dihydroxyphenyl)urea (1e)

^{13}C-NMR (100 MHz, MeOH-d_4)

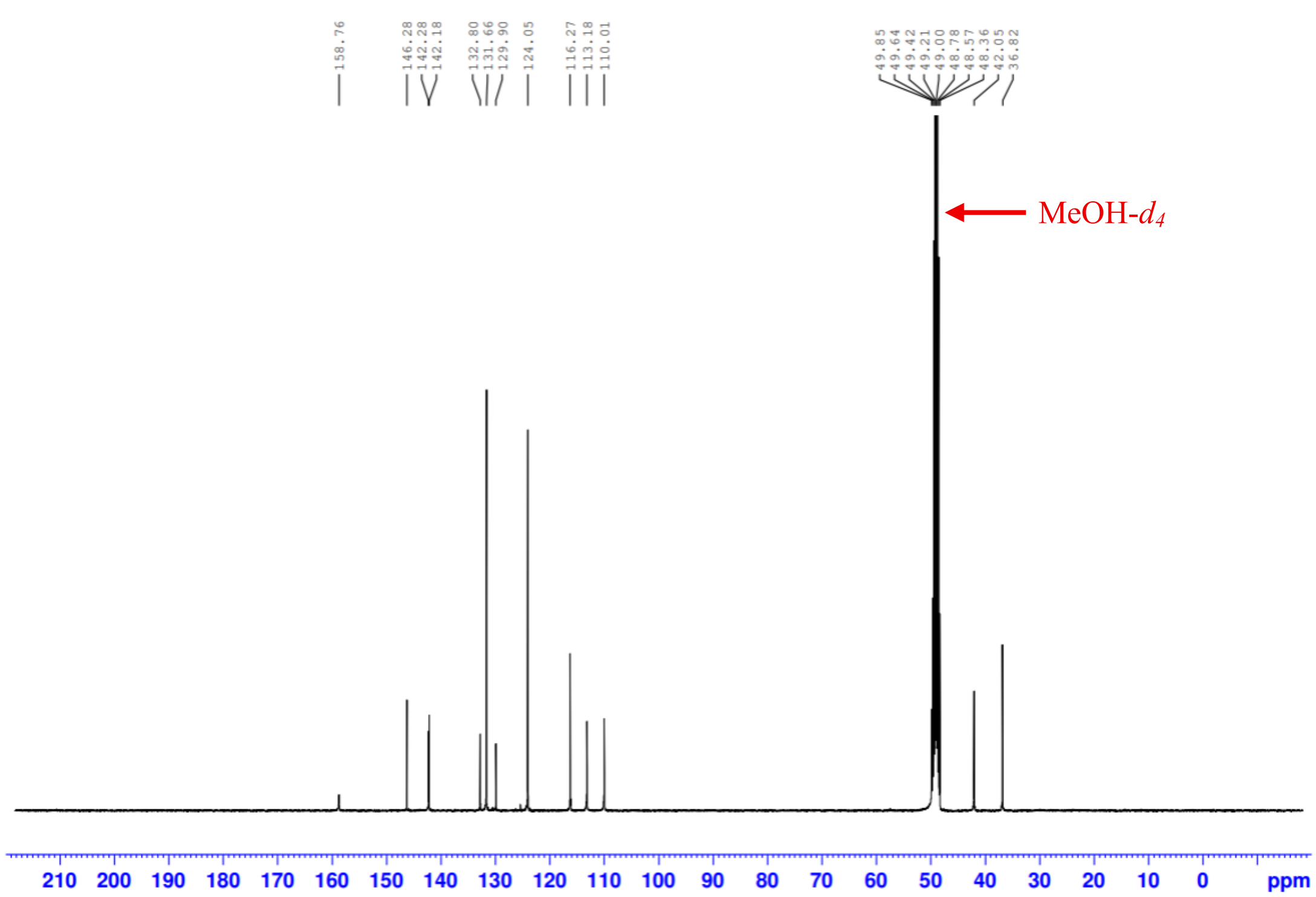

1-(4-Aminophenyl)-3-[2-(2H-1,3-benzodioxol-5-yl)ethyl]urea (8)
^{1}H-NMR (400 MHz, MeOH-d_4)

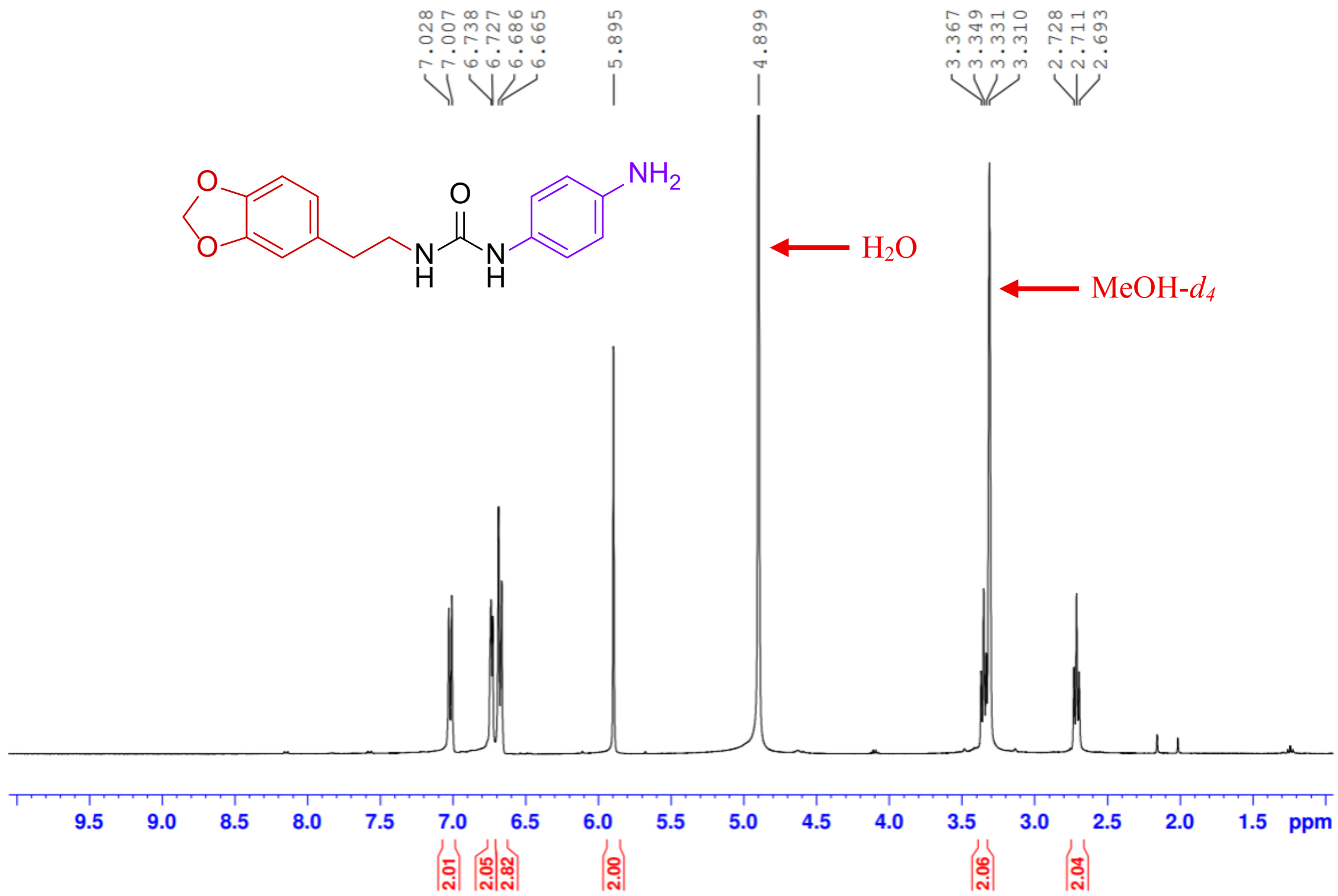

1-(4-Aminophenyl)-3-[2-(2H-1,3-benzodioxol-5-yl)ethyl]urea (8)
^{13}C-NMR (100 MHz, MeOH-d_4)

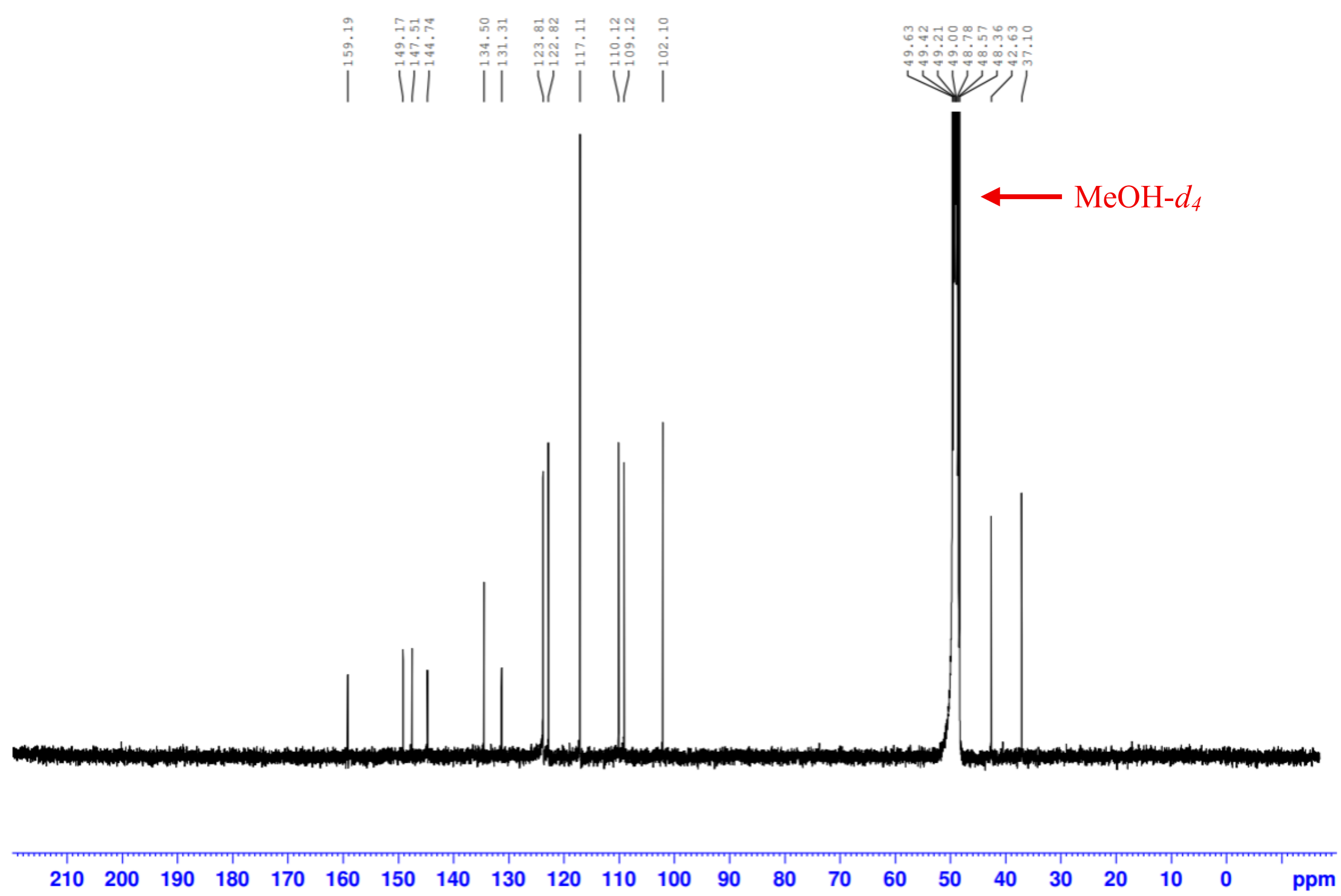

1-(4-Aminophenyl)-3-(3,4-dihydroxyphenethyl)urea (1f)
^{1}H-NMR (400 MHz, MeOH-d_4)

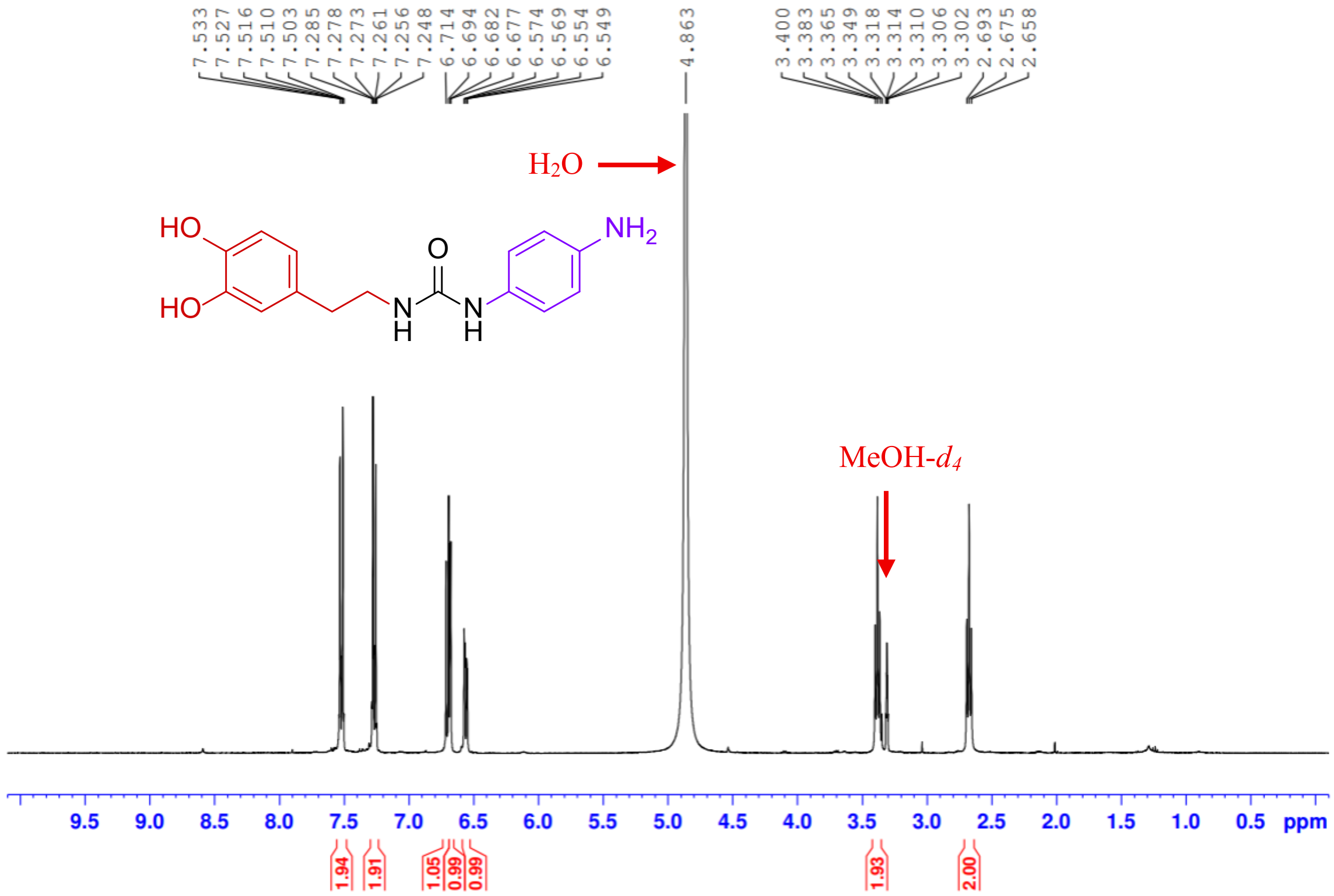

1-(4-Aminophenyl)-3-(3,4-dihydroxyphenethyl)urea (1f)
^{13}C-NMR (100 MHz, MeOH-d_4)

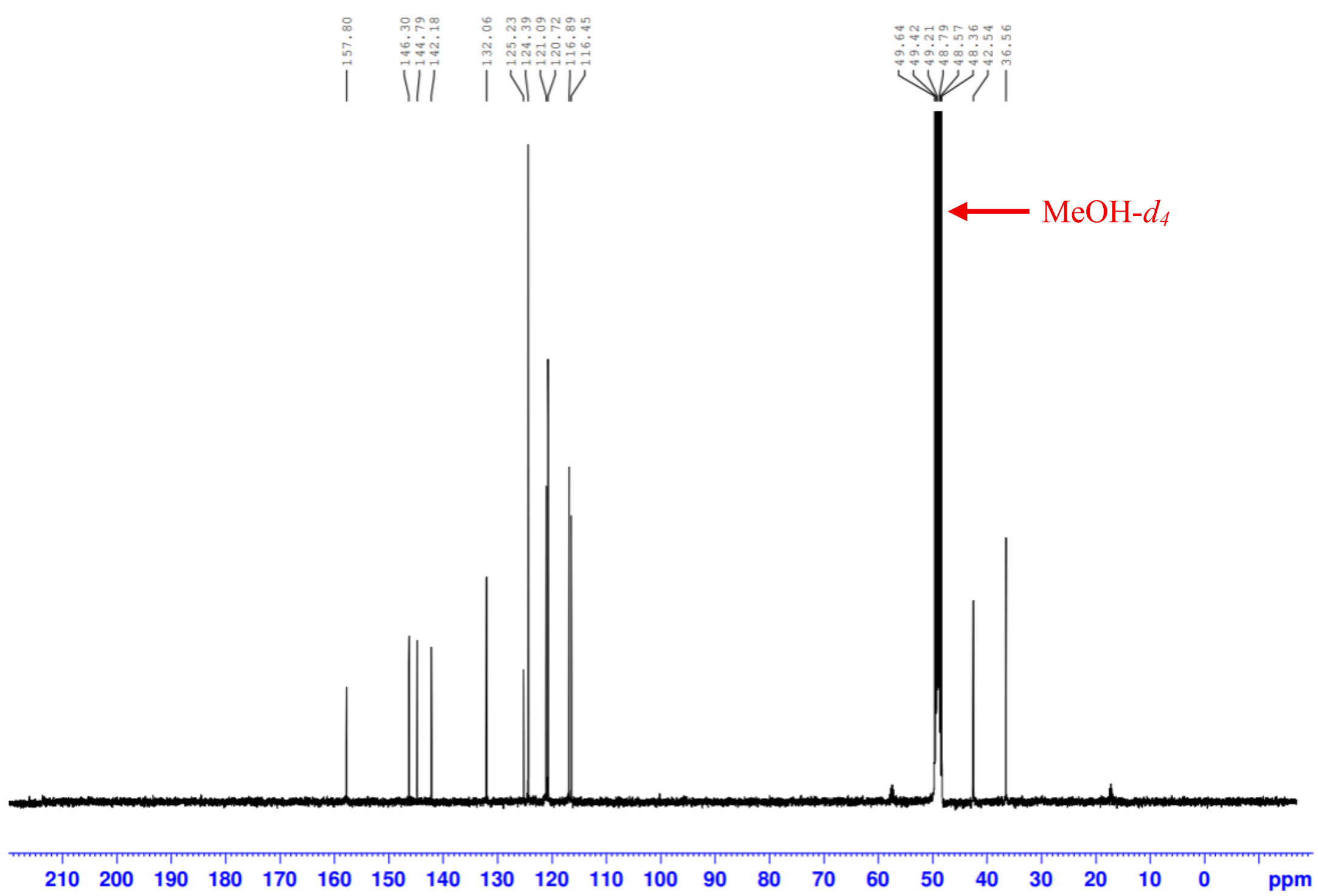

4-(3-(2-(Benzo[d][1,3]dioxol-5-yl)ethyl)ureido)benzoic acid (9)

^{1}H-NMR (400 MHz, acetone-d_6)

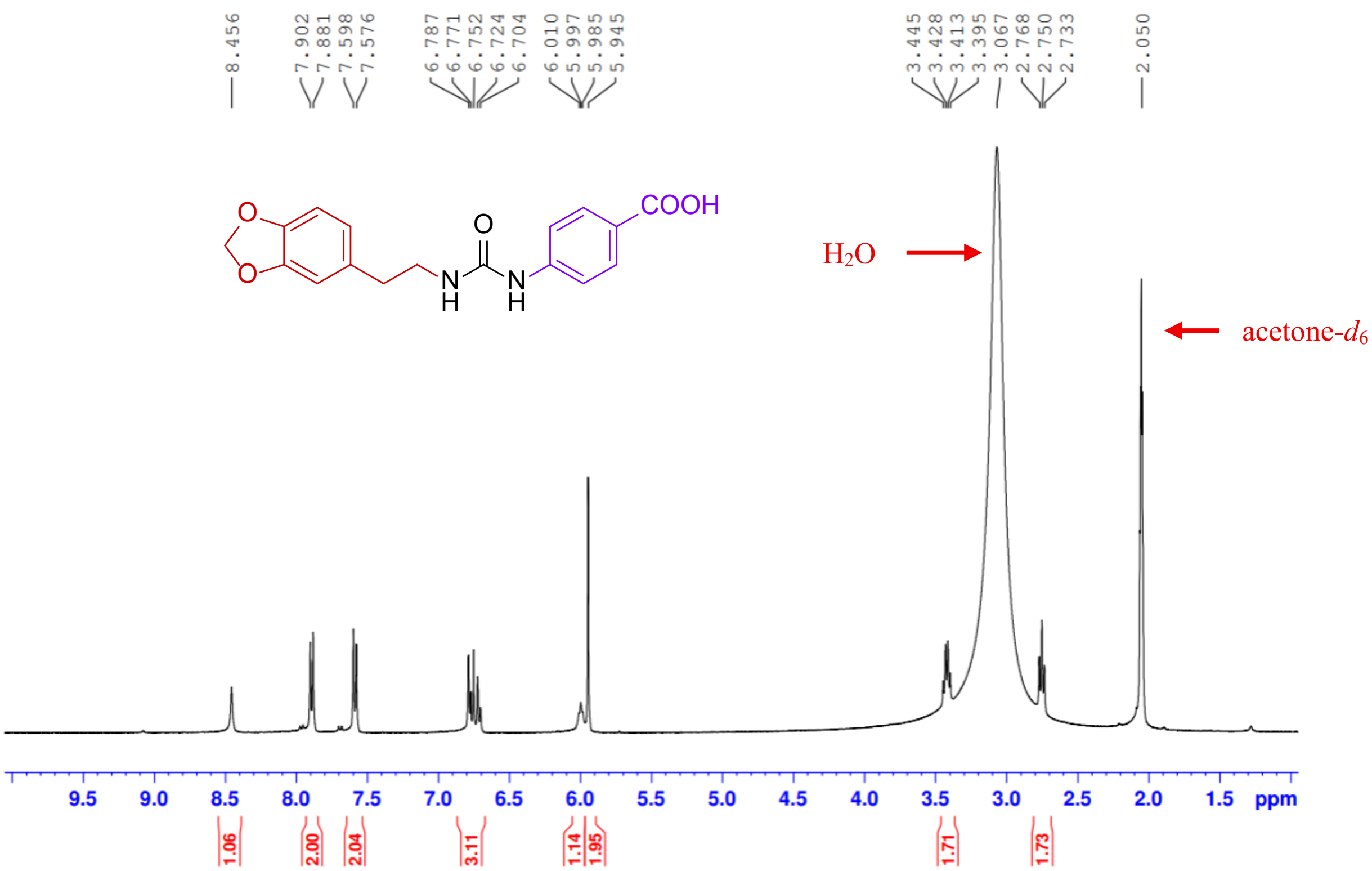

4-(3-(2-(Benzo[d][1,3]dioxol-5-yl)ethyl)ureido)benzoic acid (9)

^{13}C-NMR (100 MHz, acetone-d_6)

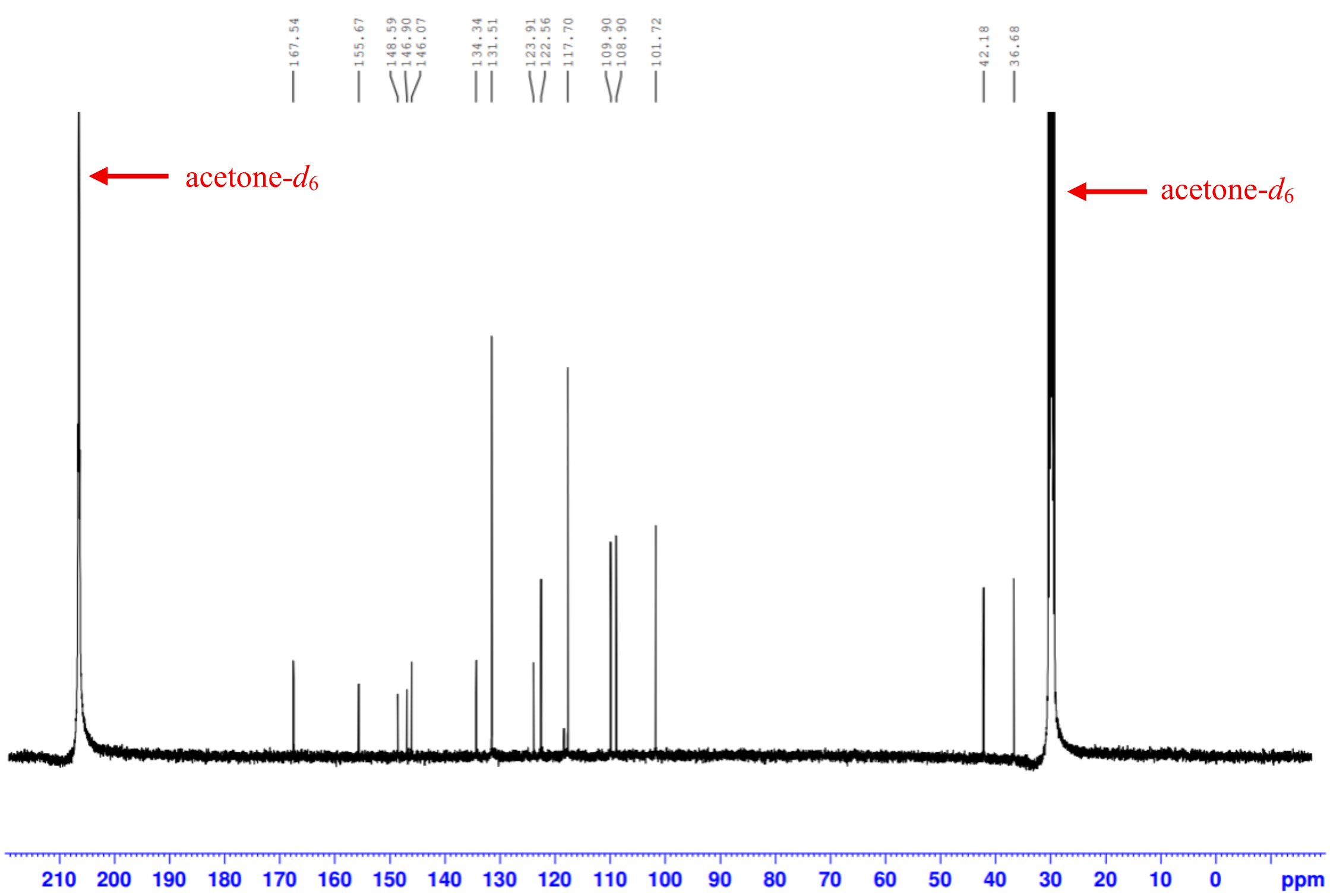

4-(3-(3,4-Dihydroxyphenethyl)ureido)benzoic acid (1g)
^{1}H-NMR (300 MHz, acetone-d_6)

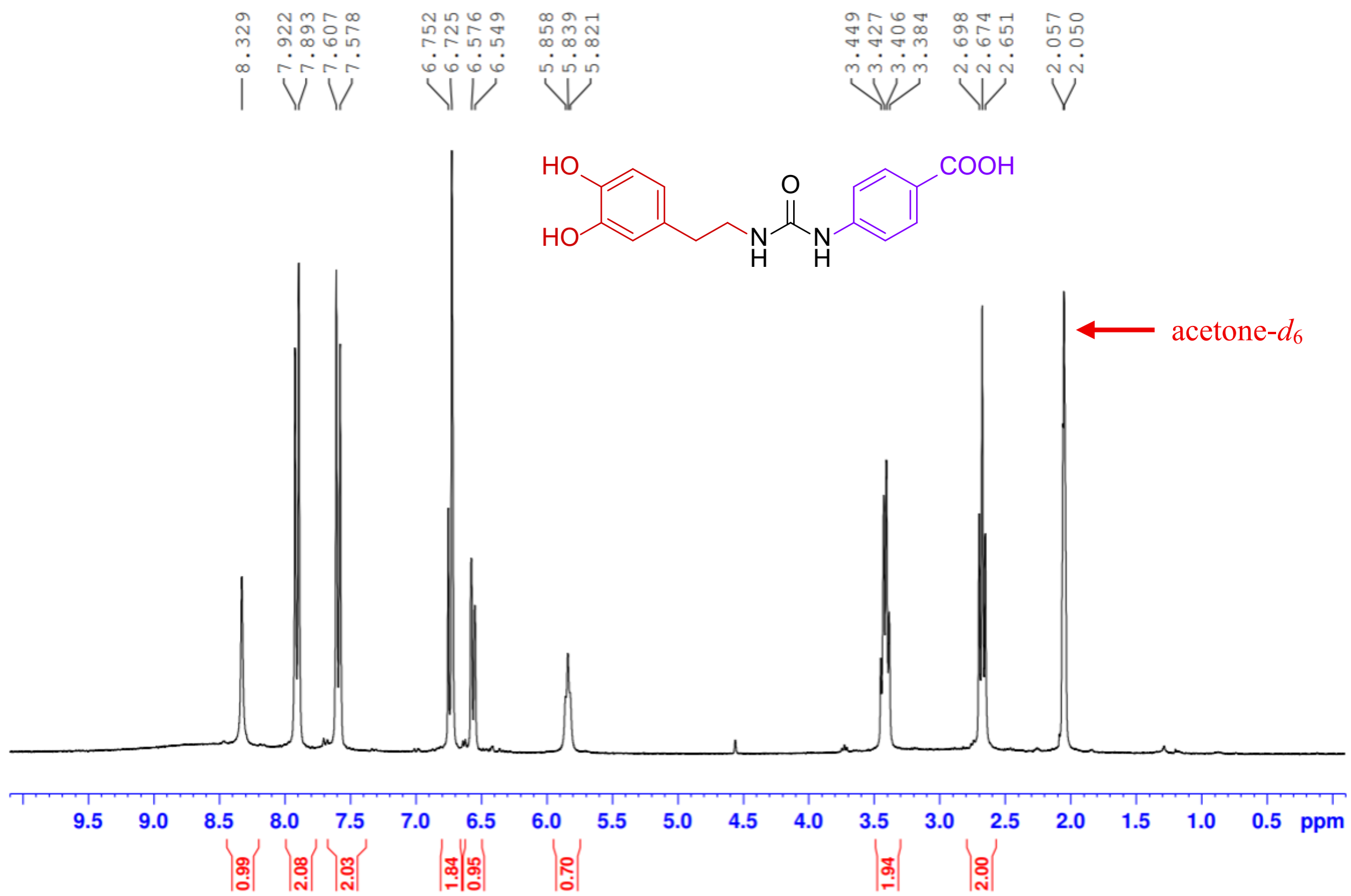

4-[3-(3,4-Dihydroxyphenethyl)ureido]benzoic acid (1g)
^{13}C-NMR (75 MHz, acetone-d_6)

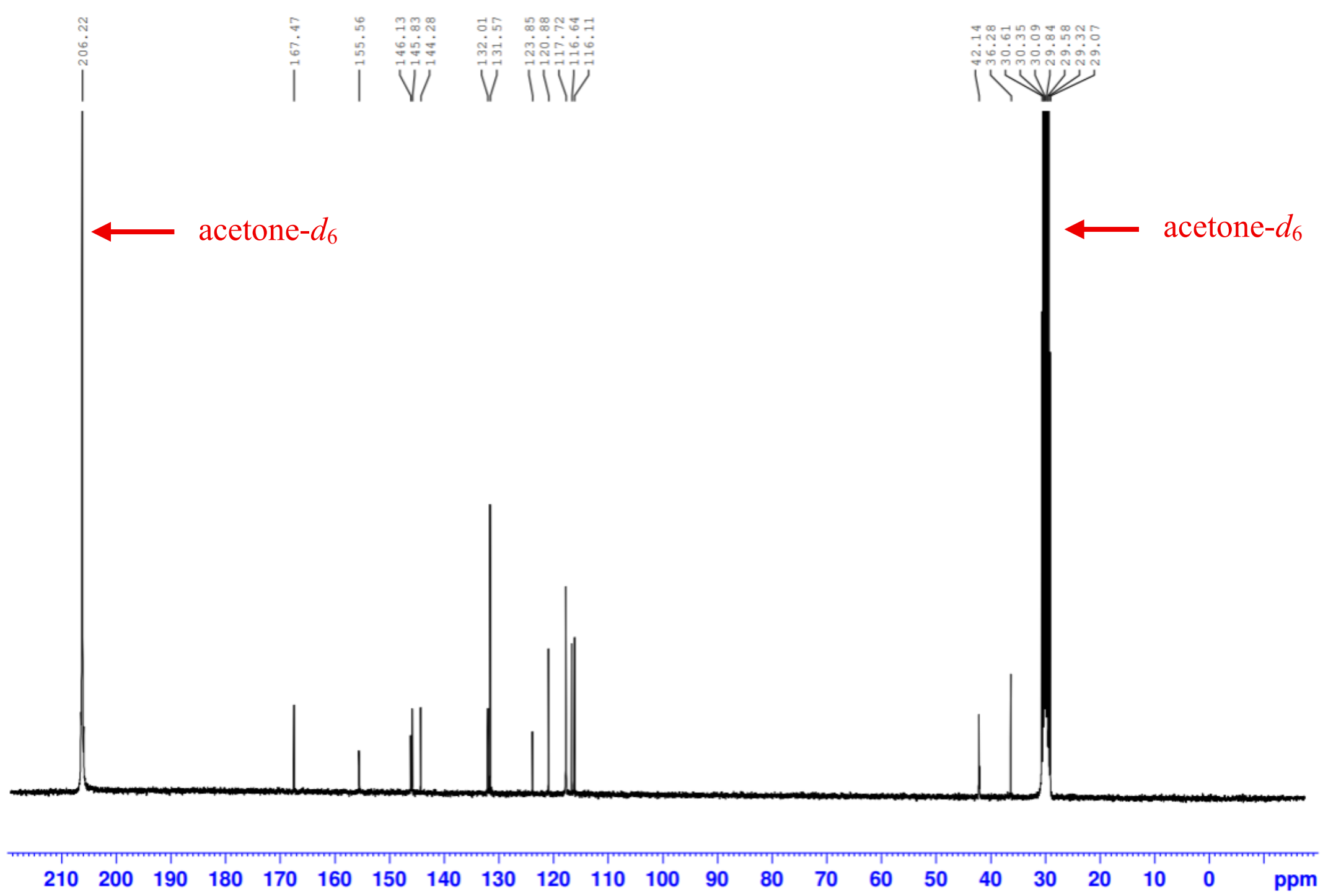

2-(Benzo[d][1,3]dioxol-5-yl)-N-(2-(benzo[d][1,3]dioxol-5-yl)ethyl)acetamide (11)

^{1}H-NMR (400 MHz, acetone-d_6)

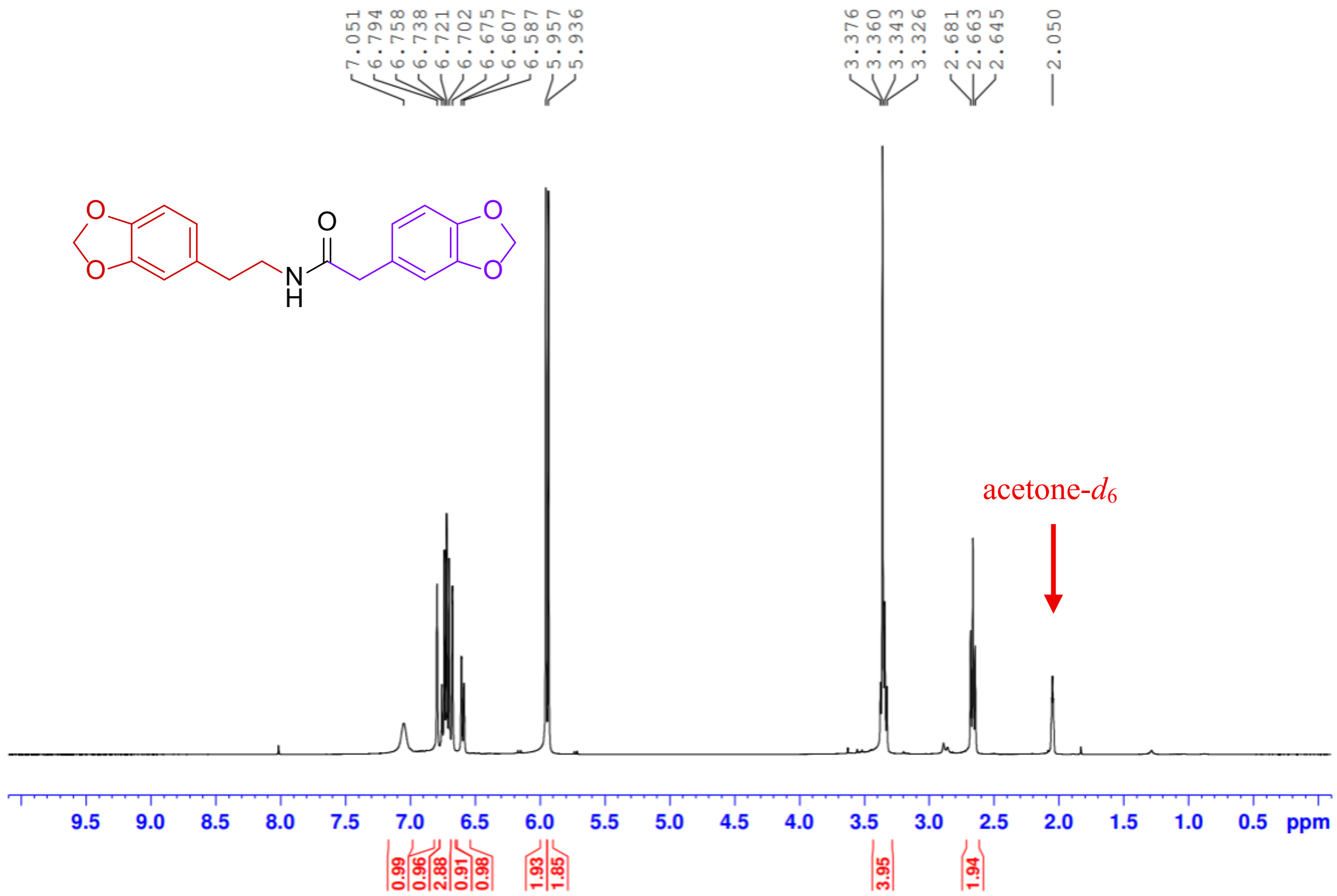

2-(Benzo[d][1,3]dioxol-5-yl)-N-(2-(benzo[d][1,3]dioxol-5-yl)ethyl)acetamide (11)

^{13}C NMR (100 MHz, acetone-d_6)

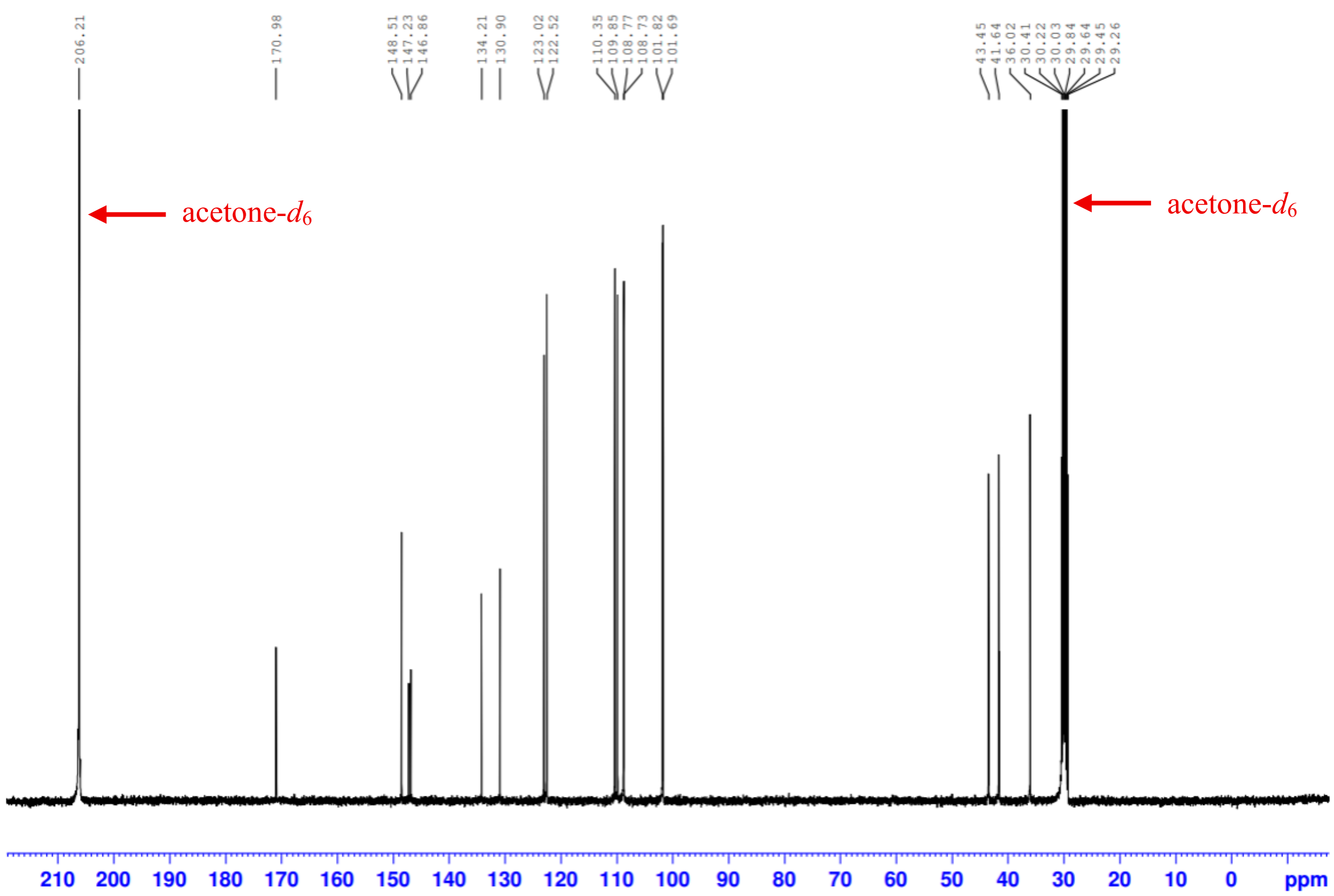

N-(3,4-Dihydroxyphenethyl)-2-(3,4-dihydroxyphenyl)acetamide (1h)

^{1}H-NMR (400 MHz, acetone-d_6)

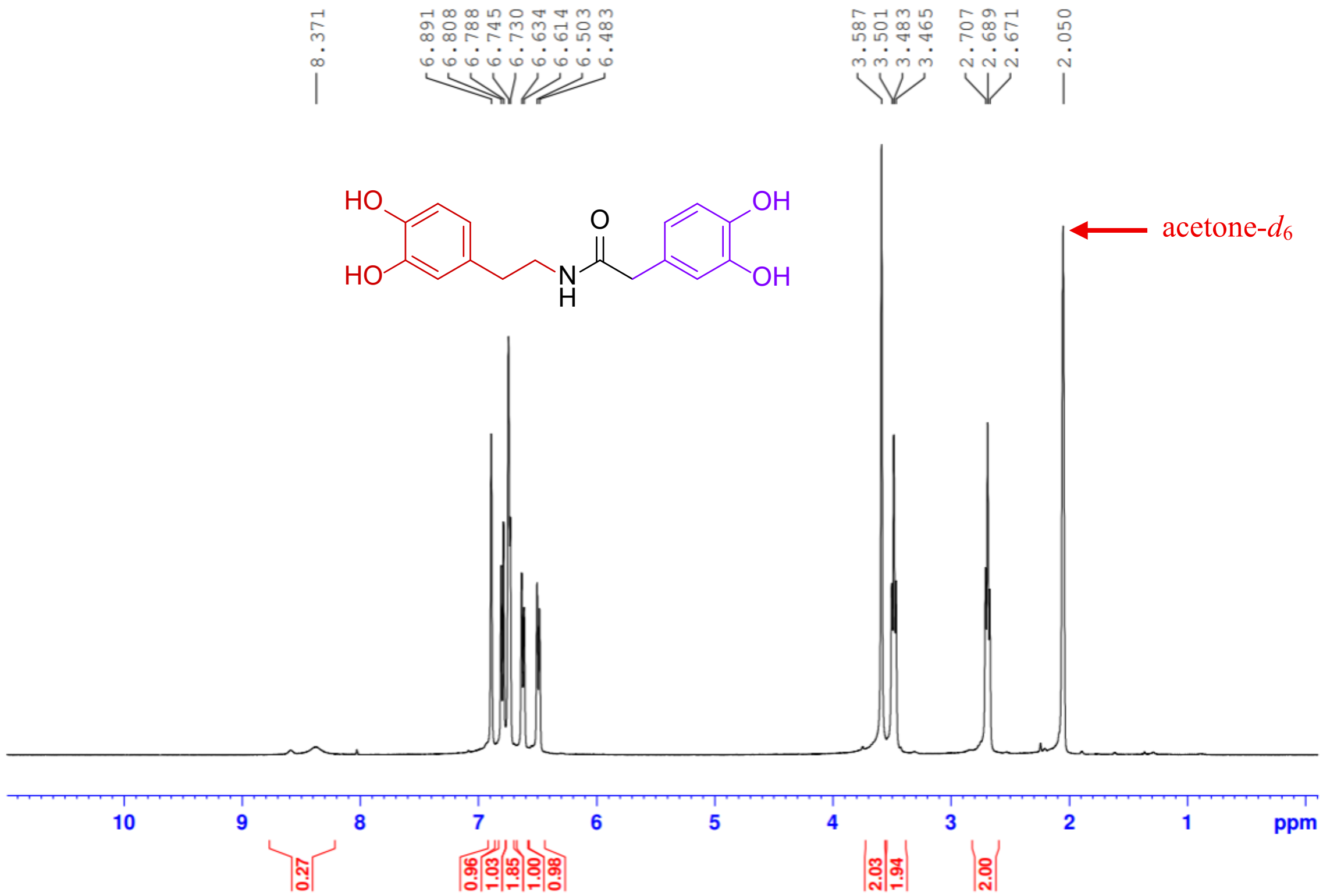

N-(3,4-Dihydroxyphenethyl)-2-(3,4-dihydroxyphenyl)acetamide (1h)

^{13}C-NMR (100 MHz, acetone-d_6)

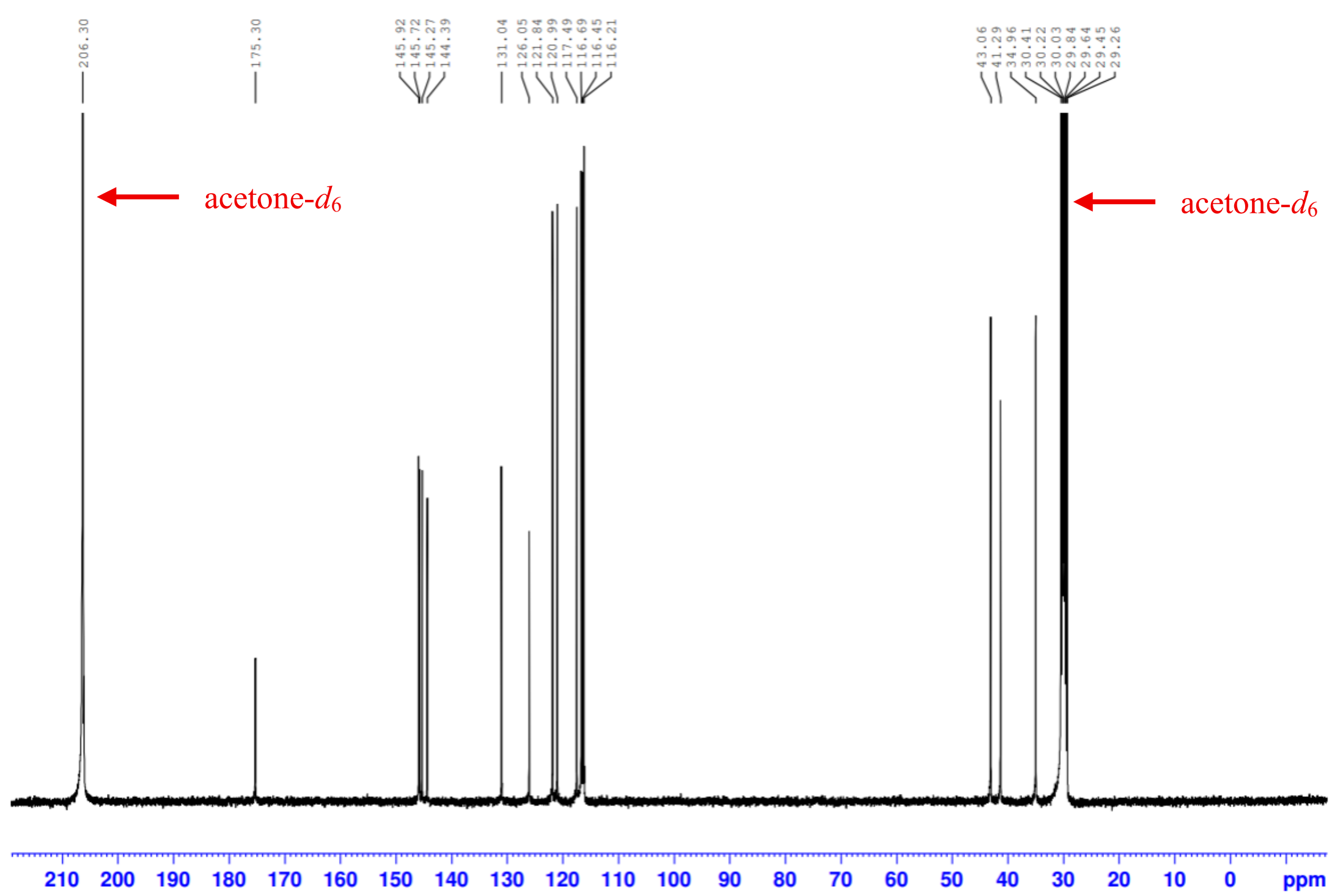

1,3-Bis(2-(benzo[d][1,3]dioxol-5-yl)ethyl)urea (13)

^{1}H-NMR (400 MHz, DMSO-d_6)

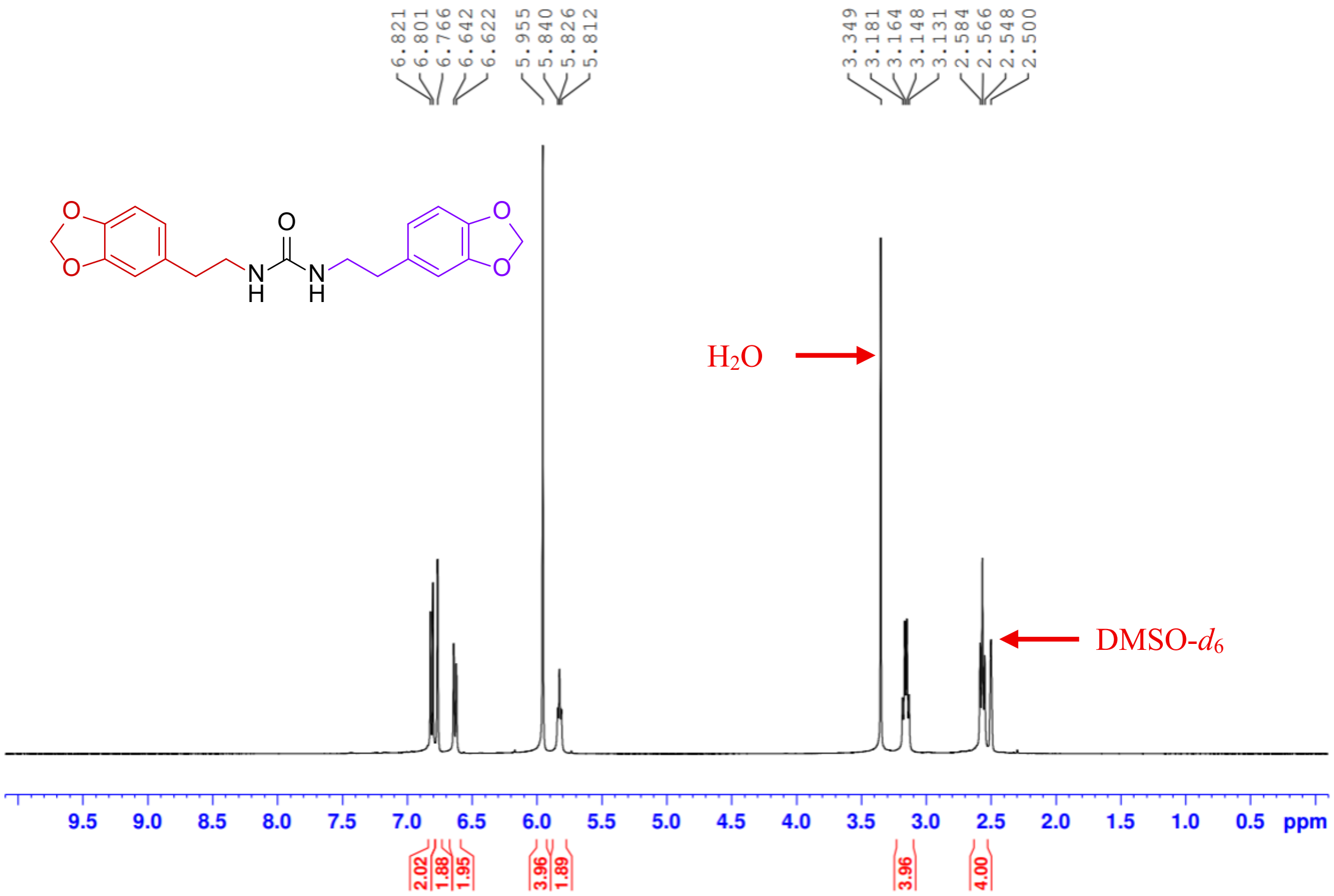

1,3-Bis(2-(benzo[d][1,3]dioxol-5-yl)ethyl)urea (13)

^{13}C-NMR (100 MHz, DMSO-d_6)

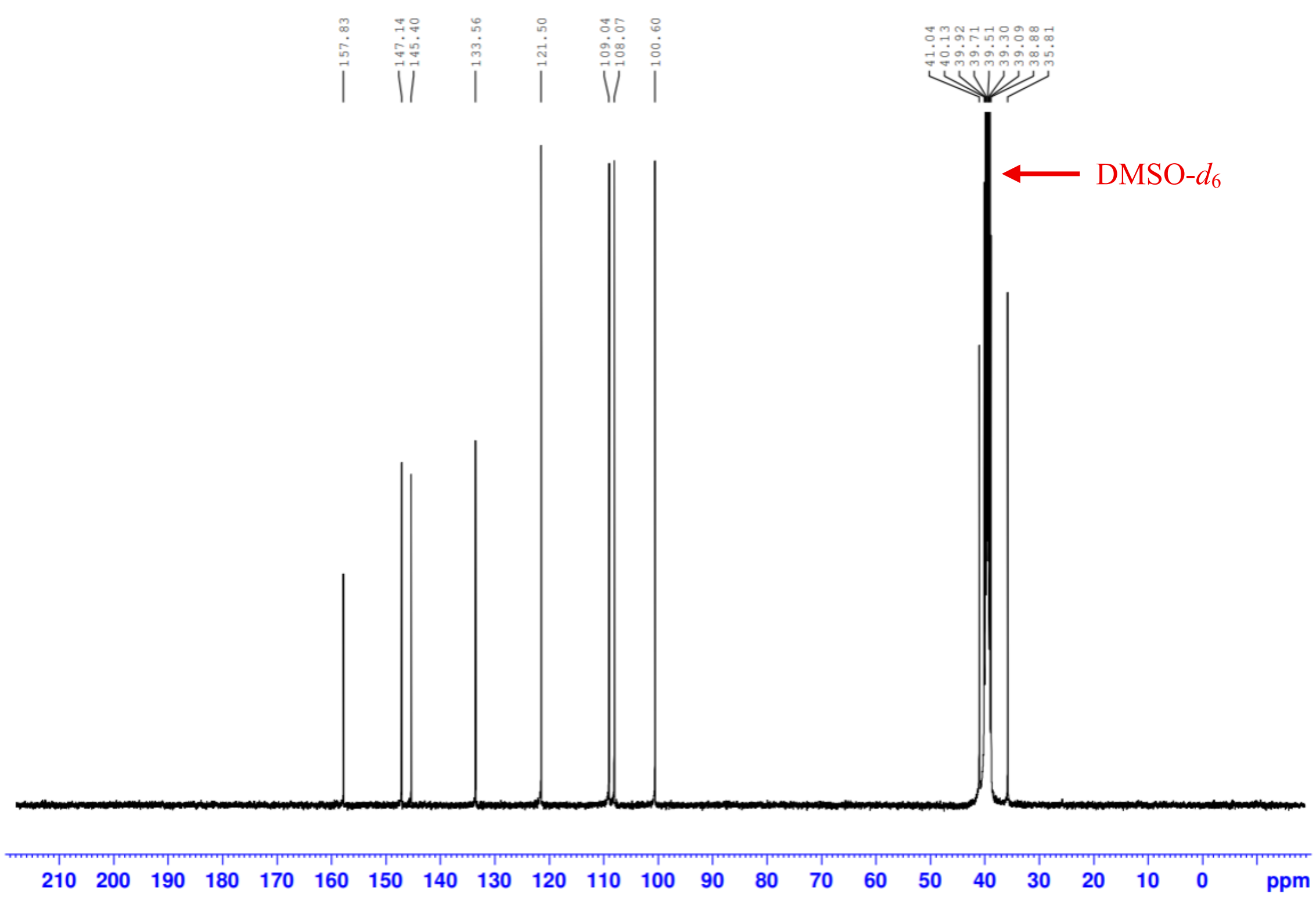

1,3-Bis(3,4-dihydroxyphenethyl)urea (1i)
^{1}H-NMR (400 MHz, acetone-d_6)

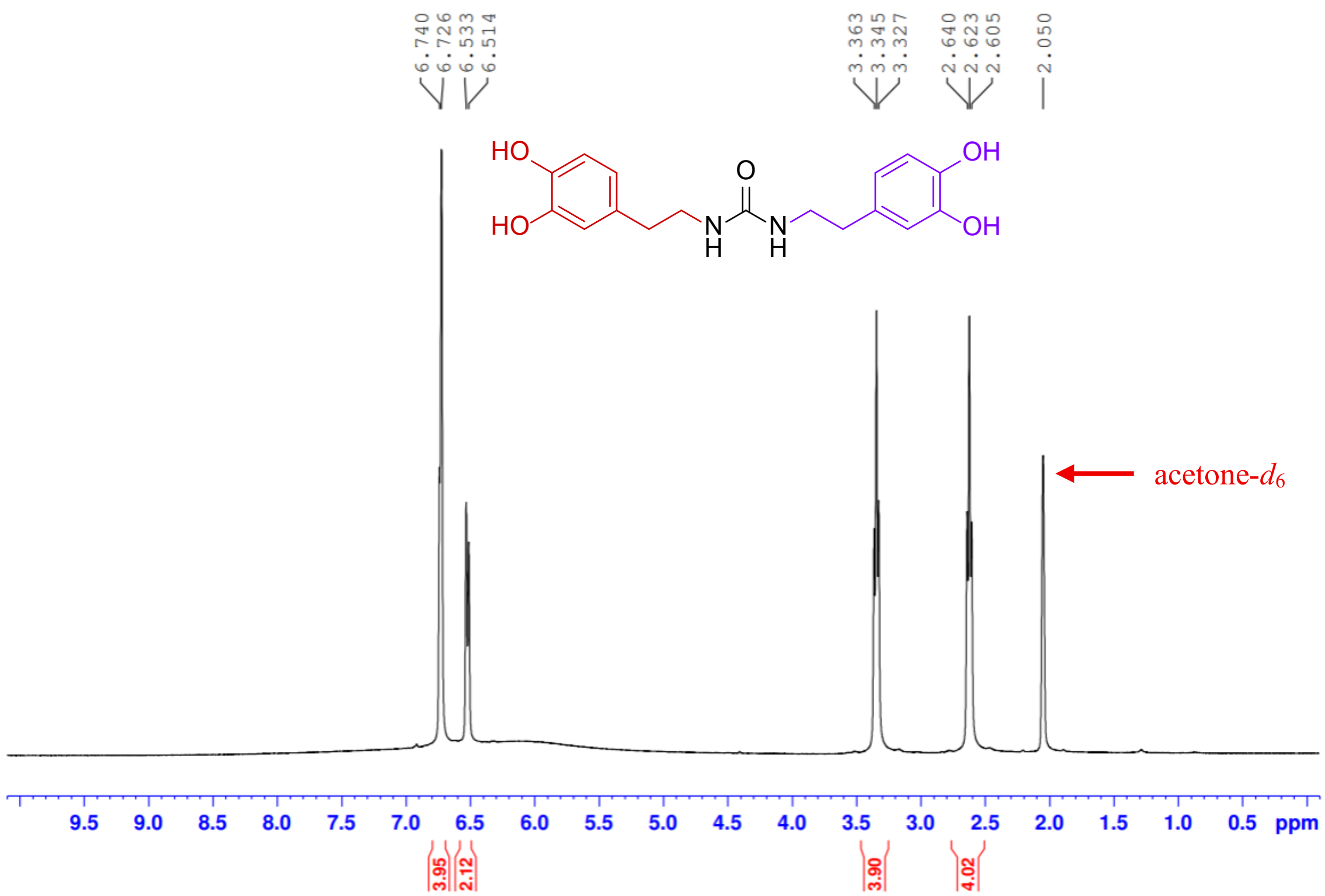

1,3-Bis(3,4-dihydroxyphenethyl)urea (1i)
^{13}C-NMR (100 MHz, acetone-d_6)

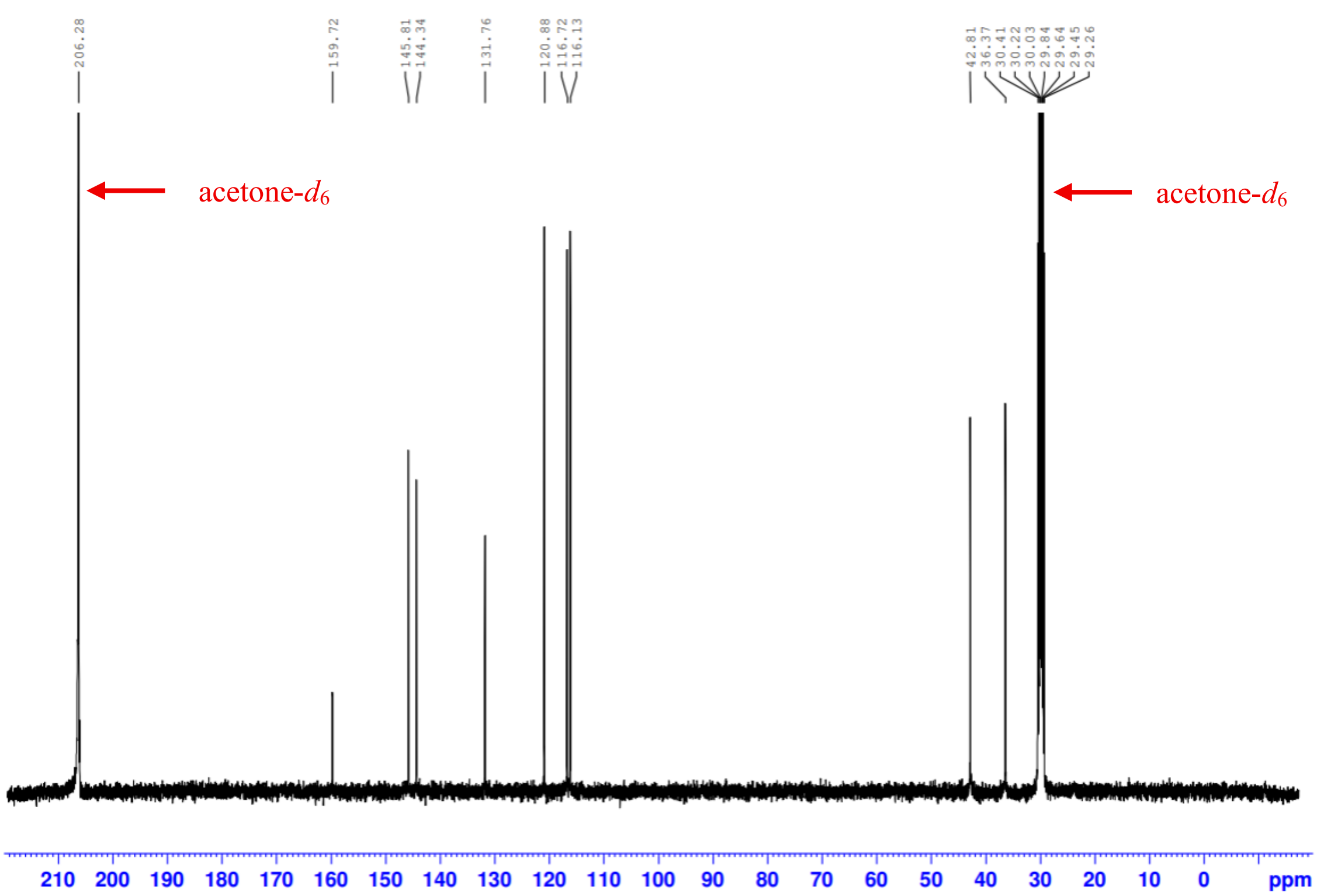

1-(Benzo[d][1,3]dioxol-5-yl)-3-(2-(benzo[d][1,3]dioxol-5-yl)ethyl)thiourea (15)

^{1}H-NMR (400 MHz, $CDCl_3$)

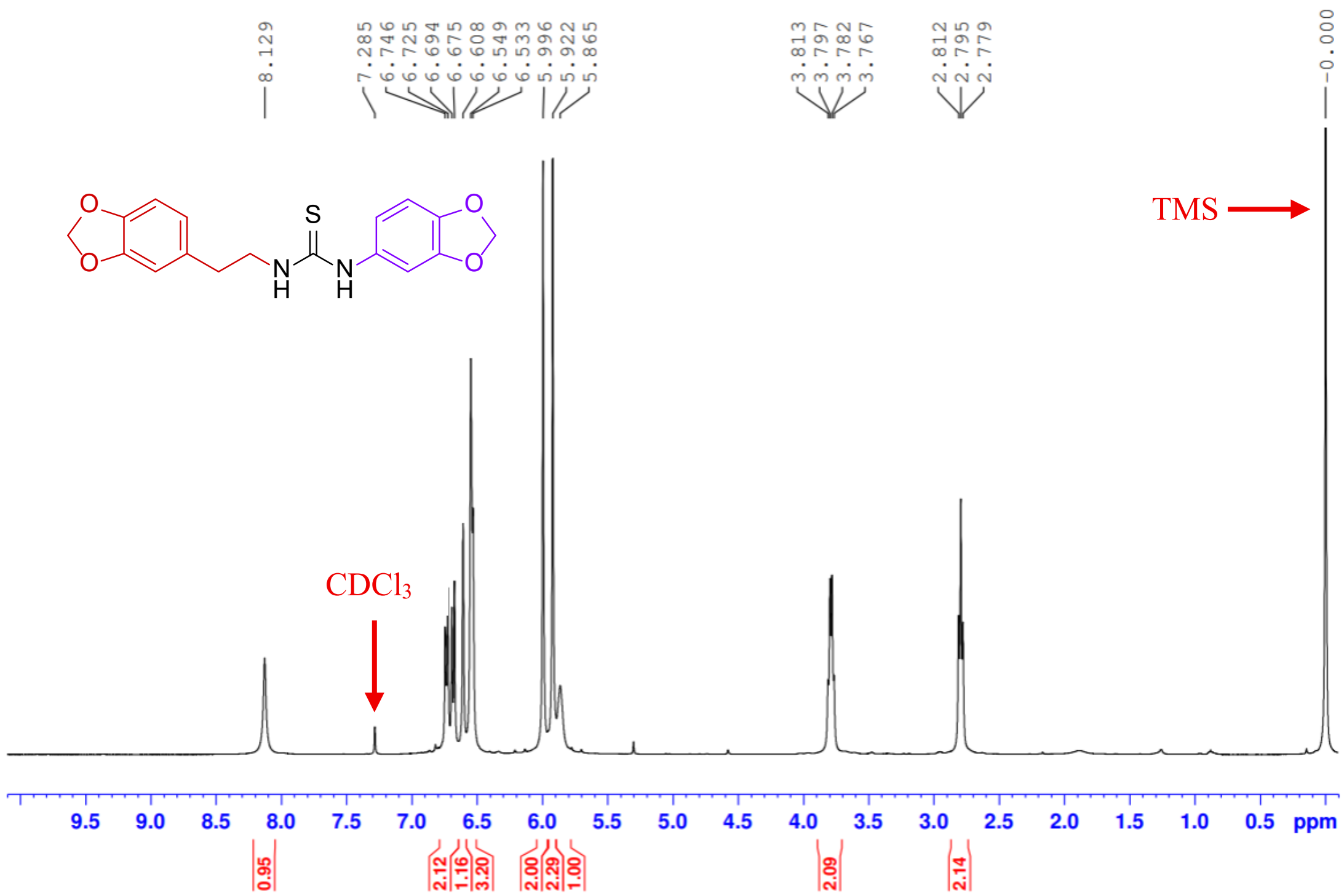

1-(Benzo[d][1,3]dioxol-5-yl)-3-(2-(benzo[d][1,3]dioxol-5-yl)ethyl)thiourea (15)

^{13}C-NMR (100 MHz, $CDCl_3$)

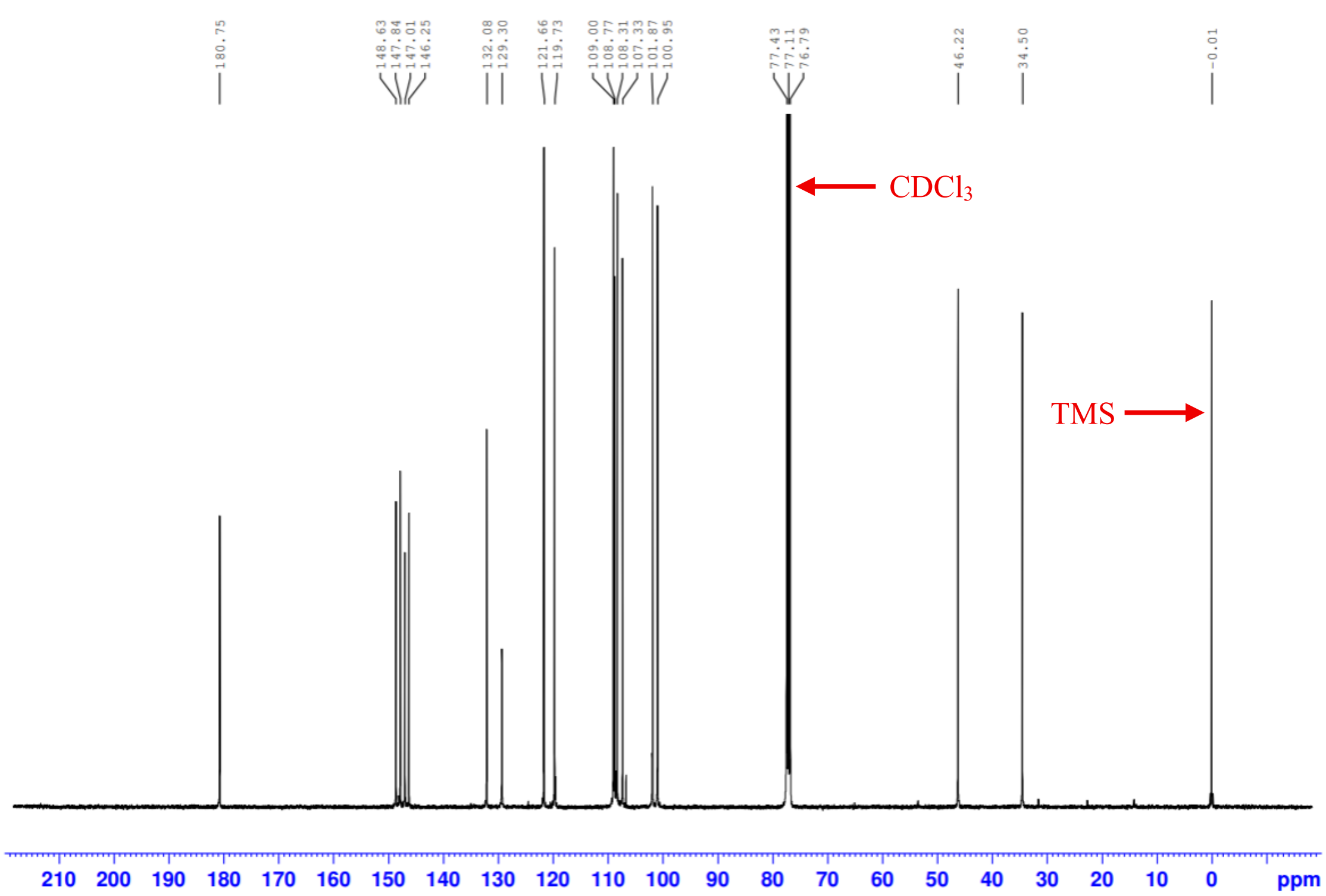

1-(3,4-Dihydroxyphenethyl)-3-(3,4-dihydroxyphenyl)thiourea (1j)
^{1}H-NMR (400 MHz, acetone-d_6)

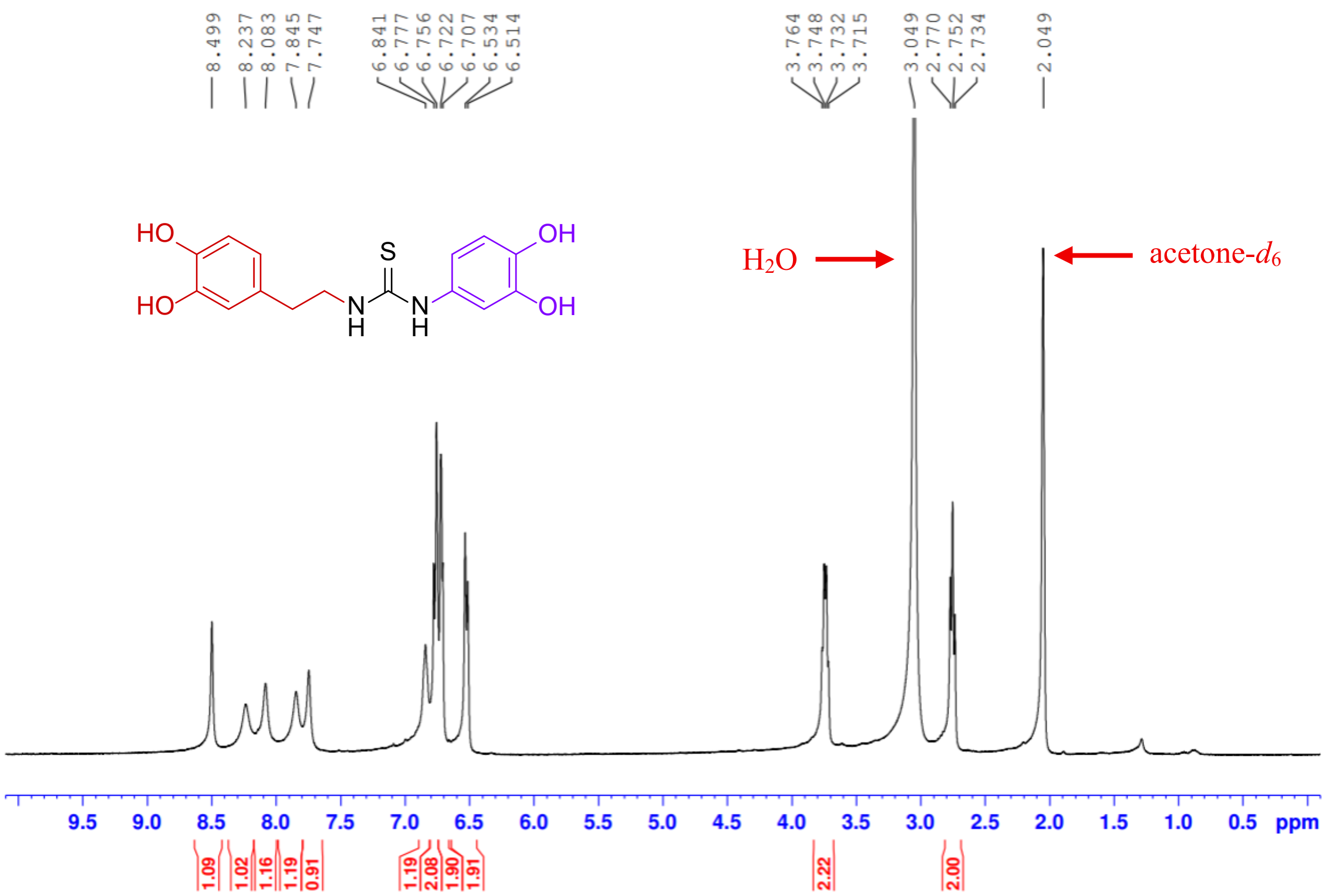

1-(3,4-Dihydroxyphenethyl)-3-(3,4-dihydroxyphenyl)thiourea (1j)
^{13}C-NMR (100 MHz, acetone-d_6)

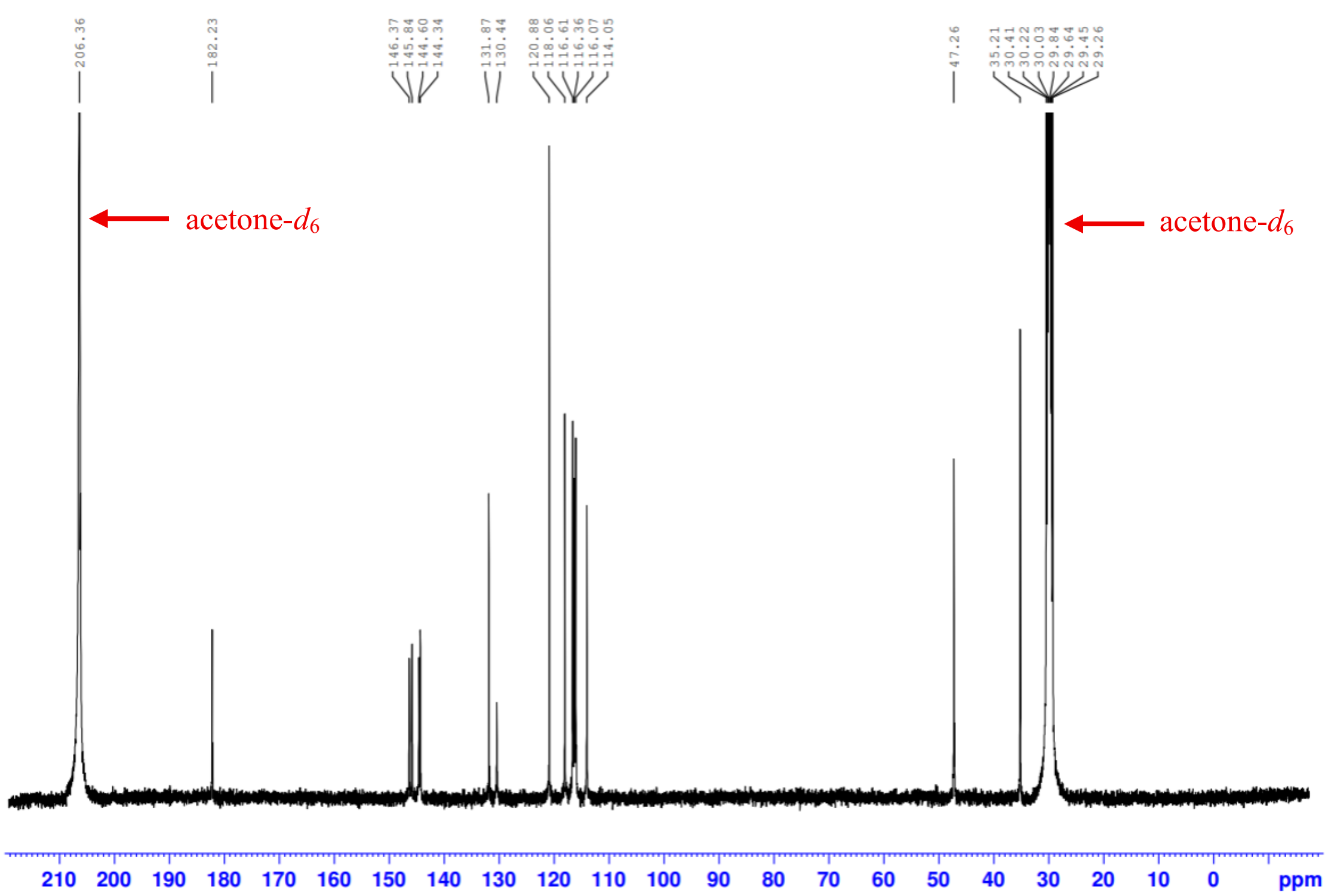

11. HPLC chromatograms of compounds

A Shimadzu high-performance liquid chromatography (HPLC) system was used to determine purity, comprised of the following units: SIL-20AC HT Prominence Auto Sampler, 2 x LC-20AD Prominence Liquid Chromatographs, SPD-M20A Prominence Diode Array Detector, CTO-20AC Prominence Column Oven, DGU-20A3 Prominence Degasser, and FCV-11AL Valve Unit. A reverse-phase Kinetex 5 µm C18 100 Å 150 x 4.6 mm column was used for all (HPLC) runs and chromatograms were recorded using UV detection at 254 nm. The chromatograms were exported and plotted using GraphPad Prism 10.6.1. Chromatograms of null injects and DMSO as the sample solvent were included for comparison. All methods were run at a flow rate of 1 mL/min and an oven temperature of 30 °C using water as mobile phase A and either acetonitrile or methanol as mobile phase B.

Below the methods used for HPLC are described:

Method A: This HPLC method was run with water as mobile phase A and acetonitrile as mobile phase B. The run began at 10 % mobile phase B and at t= 0 minutes mobile phase B was increased over 10 minutes to 95 %. At t= 15 minutes mobile phase B was decreased to 10 % over 1 minute. The method ran for 20 minutes total.

Method B: This HPLC method was run with water as mobile phase A and methanol as mobile phase B. The run began at 10 % mobile phase B and at t= 7 minutes mobile phase B was increased over 8 minutes to 60 %. At t= 17 minutes mobile phase B was decreased to 10 % over 1 minute. The method ran for 20 minutes total.

Method C: This HPLC method was run with water as mobile phase A and methanol as mobile phase B. The run began at 35 % mobile phase B and at t= 7 minutes mobile phase B was increased over 8 minutes to 75 %. At t= 17 minutes mobile phase B was decreased to 35 % over 1 minute. The method ran for 20 minutes total.

Method D: This HPLC method was run with water as mobile phase A and acetonitrile as mobile phase B. The run began at 10 % mobile phase B and at t= 0 minutes mobile phase B was increased over 5 minutes to 30 %. At t= 7 minutes mobile phase B was decreased to 10 % over 1 minute. The method ran for 10 minutes total.

Method E: This HPLC method was run with water as mobile phase A and acetonitrile as mobile phase B. The run began at 10 % mobile phase B and at t= 0 minutes mobile phase B was increased over 5 minutes to 60 %. At t= 7 minutes mobile phase B was decreased to 10 % over 1 minute. The method ran for 10 minutes total.

***N*-(2*H*-1,3-Benzodioxol-5-yl)-*N*'-[2-(2*H*-1,3-benzodioxol-5-yl) ethyl] urea (4)**

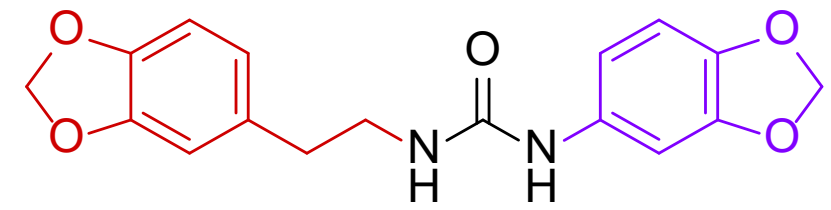

Method A was used to generate this chromatogram.

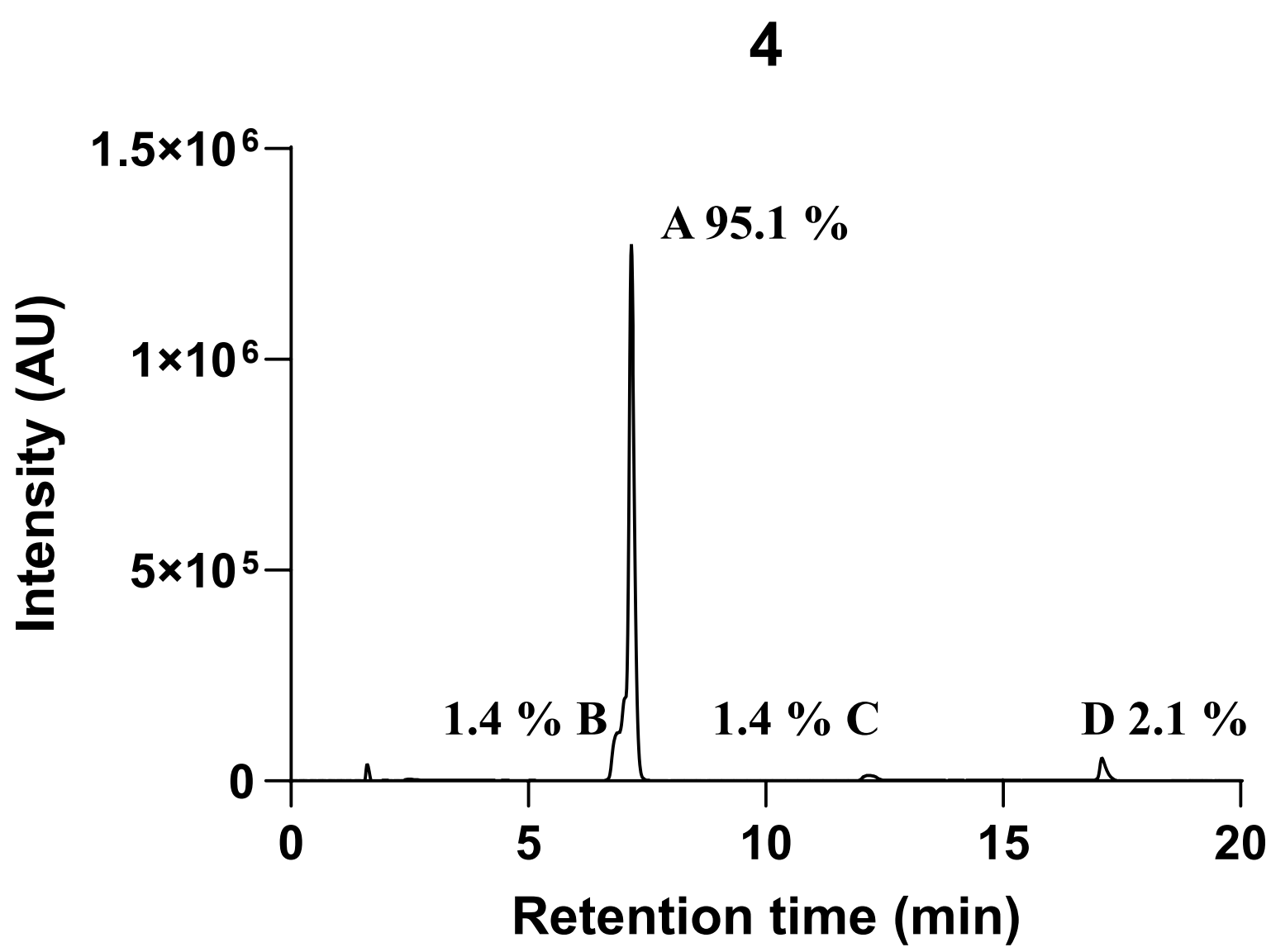

N-(3,4-Dihydroxyphenyl)-N'-[2-(3,4-dihydroxyphenyl) ethyl] urea (SN195)

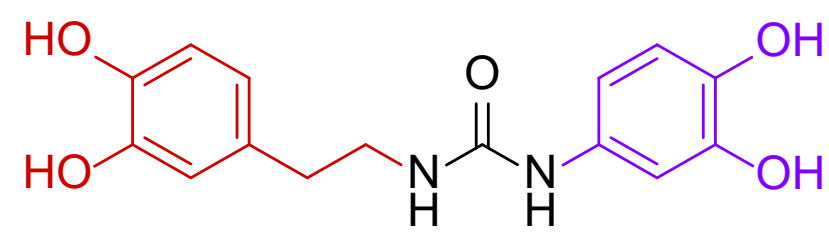

Method B was used to generate this chromatogram.

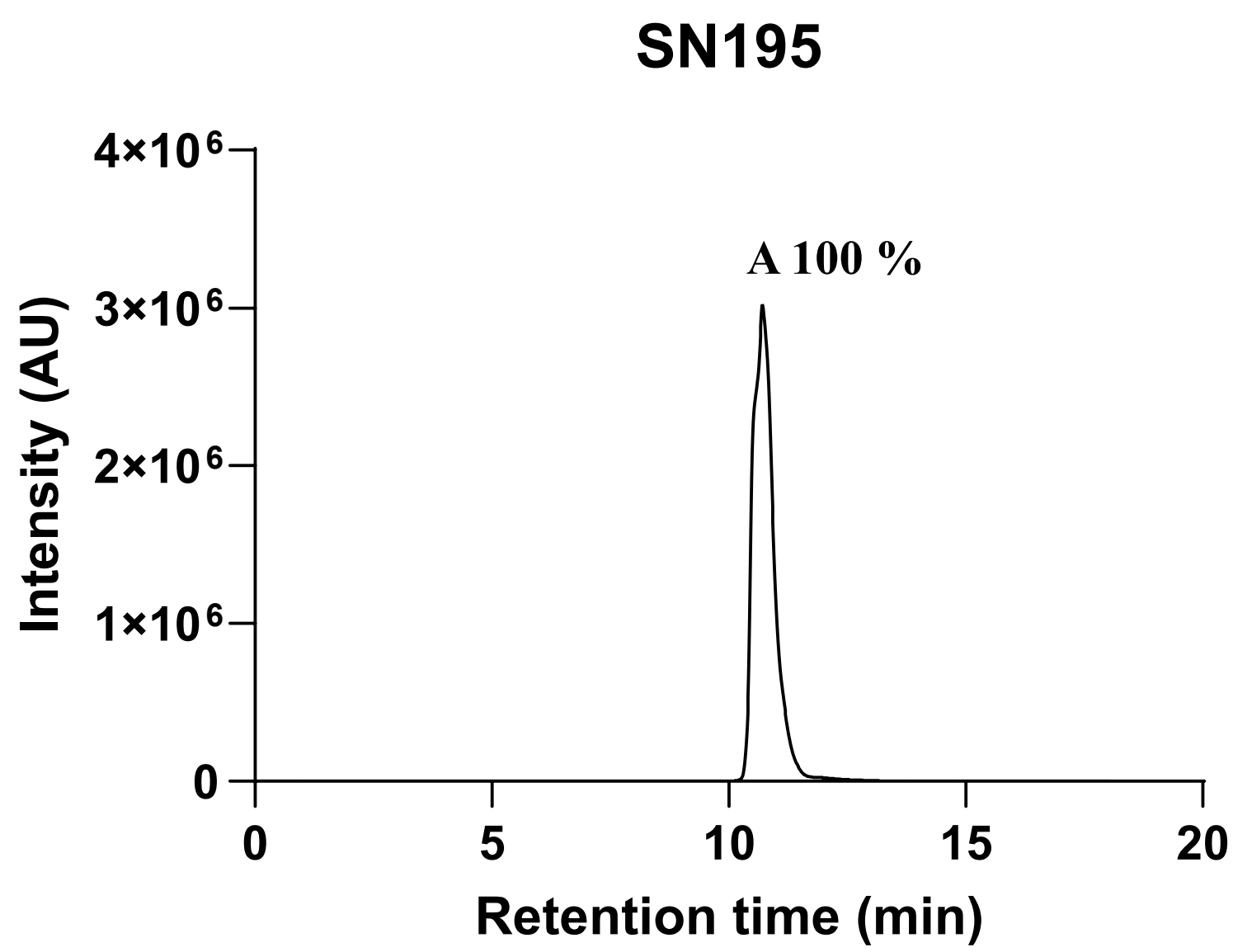

1-(2-(Benzo[d][1,3]dioxol-5-yl)ethyl)-3-(4-methoxyphenyl)urea (5a)

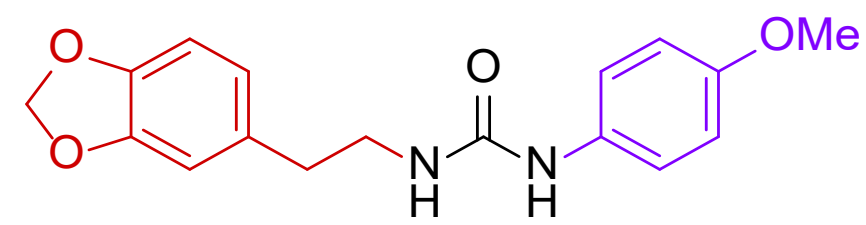

Method C was used to generate this chromatogram.

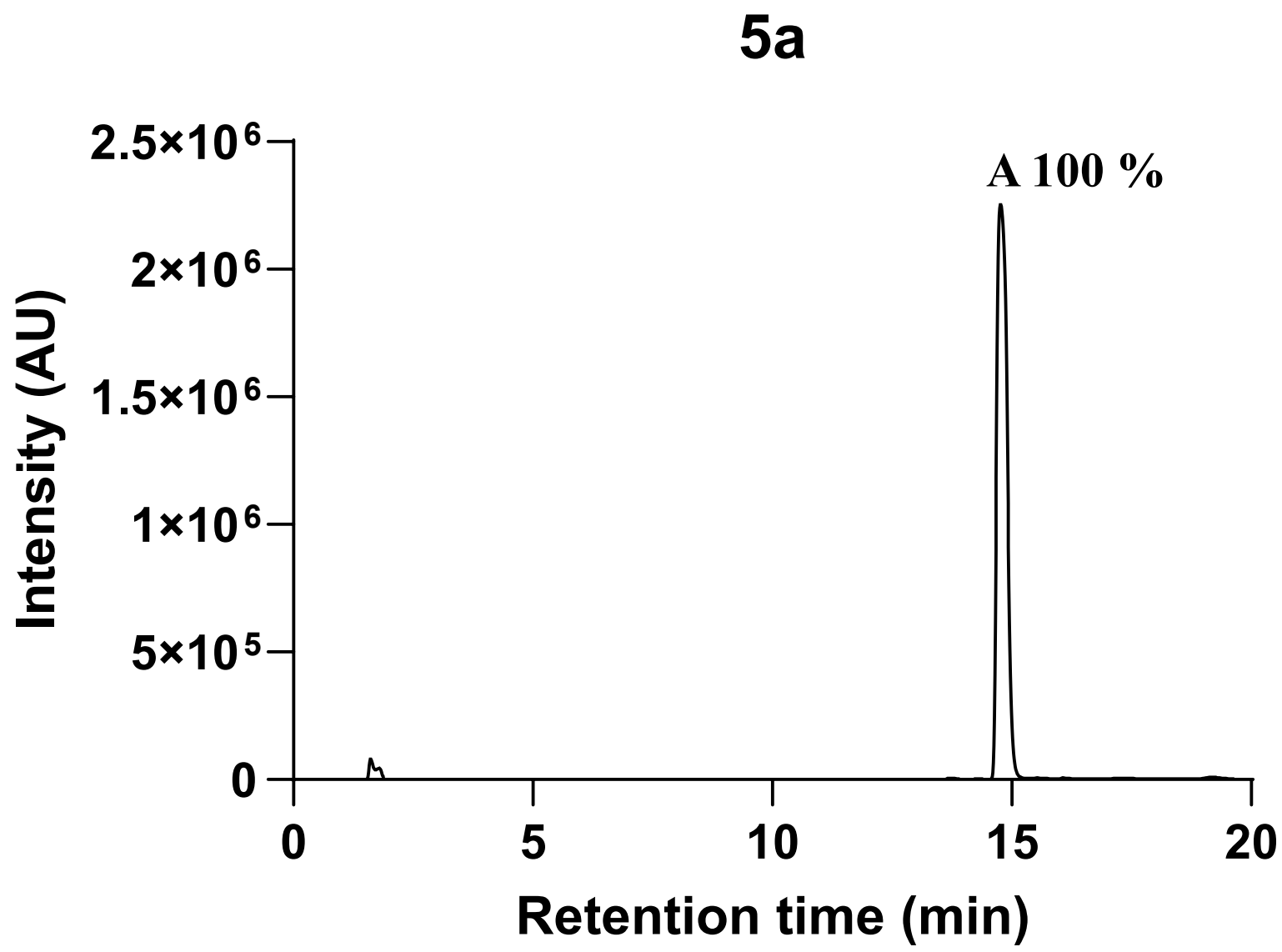

1-(Benzo[d][1,3]dioxol-5-yl)-3-(4-methoxyphenethyl)urea (5b)

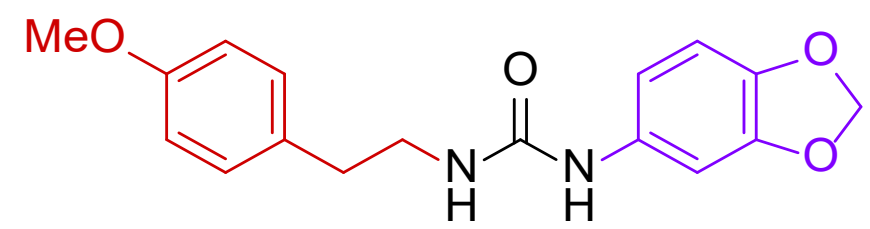

Method C was used to generate this chromatogram.

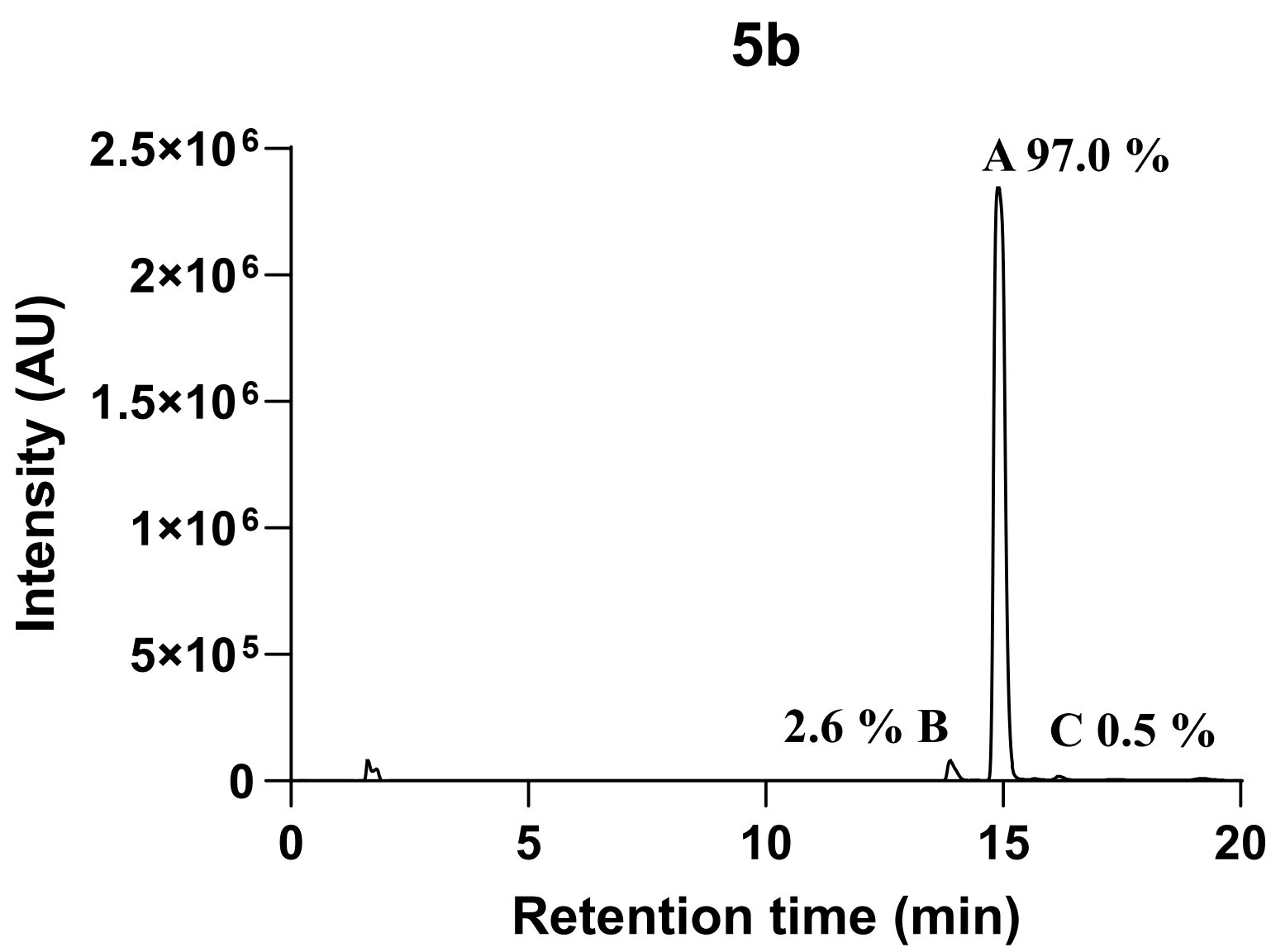

1-(2-(Benzo[d][1,3]dioxol-5-yl)ethyl)-3-(4-nitrophenyl)urea (5c)

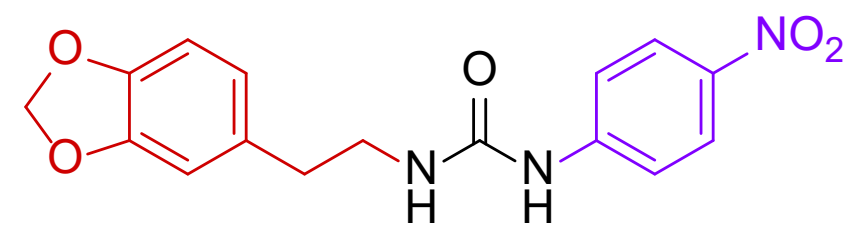

Method C was used to generate this chromatogram.

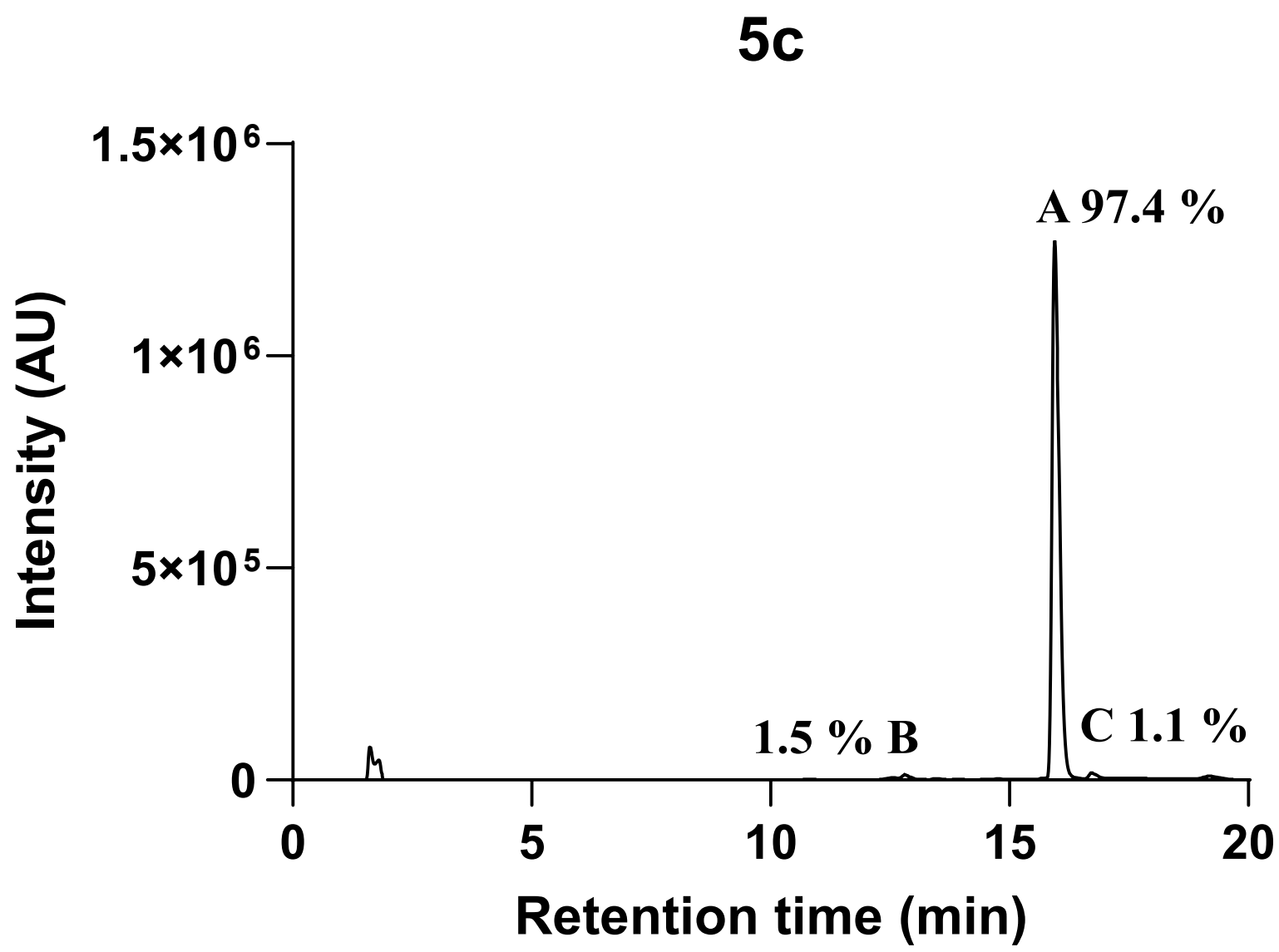

Methyl 4-(3-(2-(benzo[d][1,3]dioxol-5-yl)ethyl)ureido)benzoate (5d)

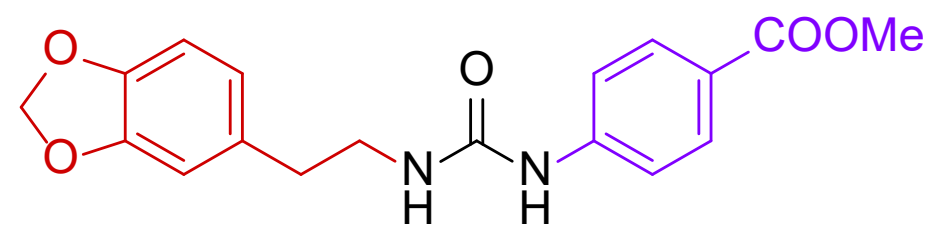

Method C was used to generate this chromatogram.

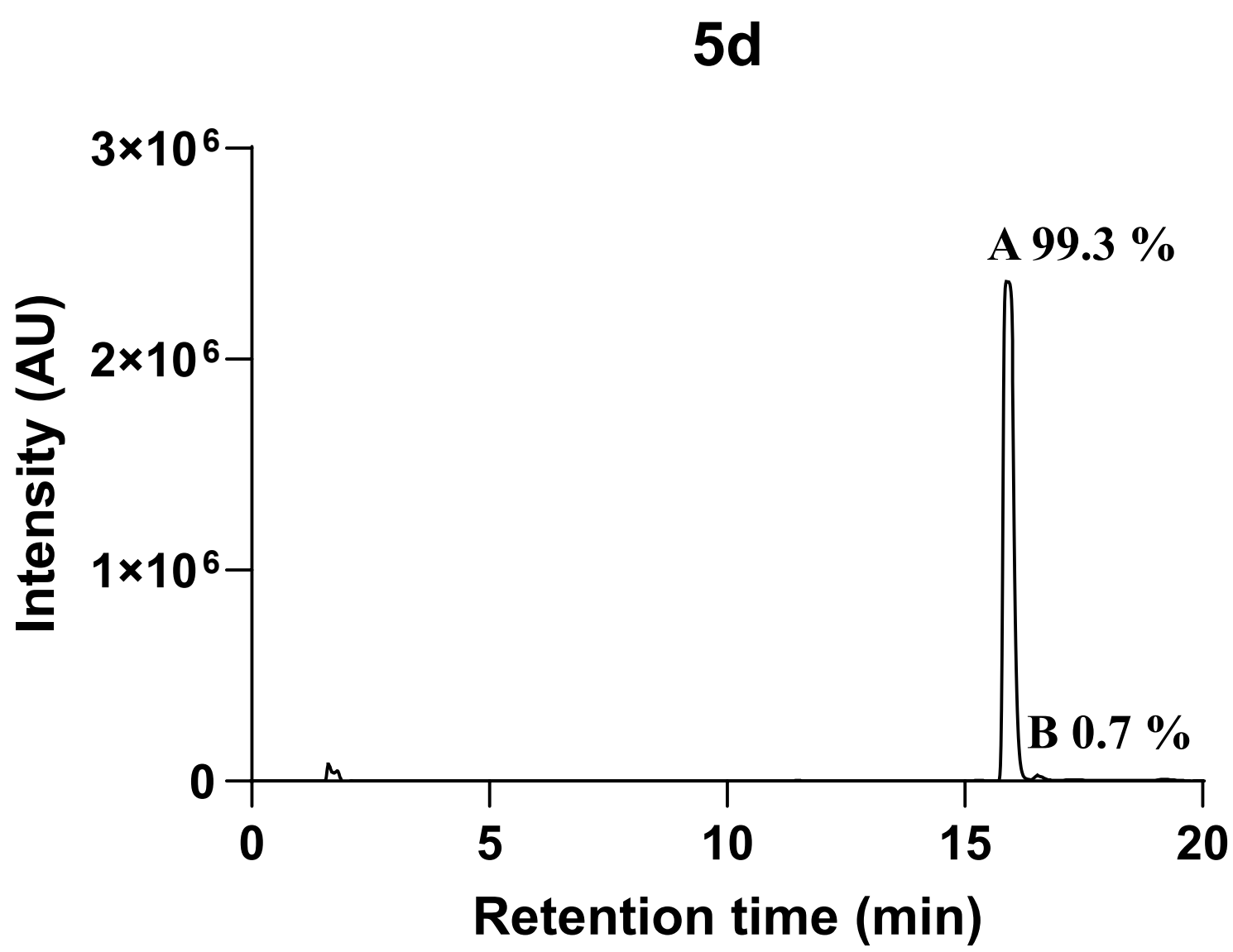

1-(3,4-Dihydroxyphenethyl)-3-(4-hydroxyphenyl)urea (1a)

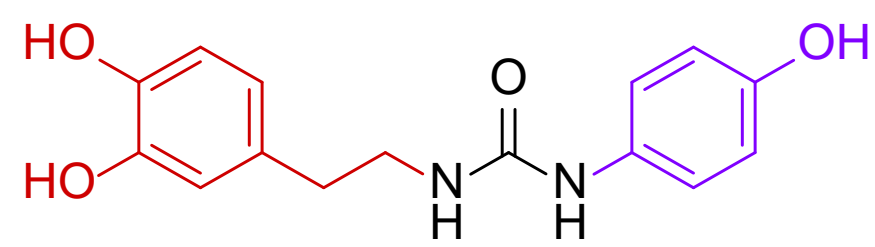

Method D was used to generate this chromatogram.

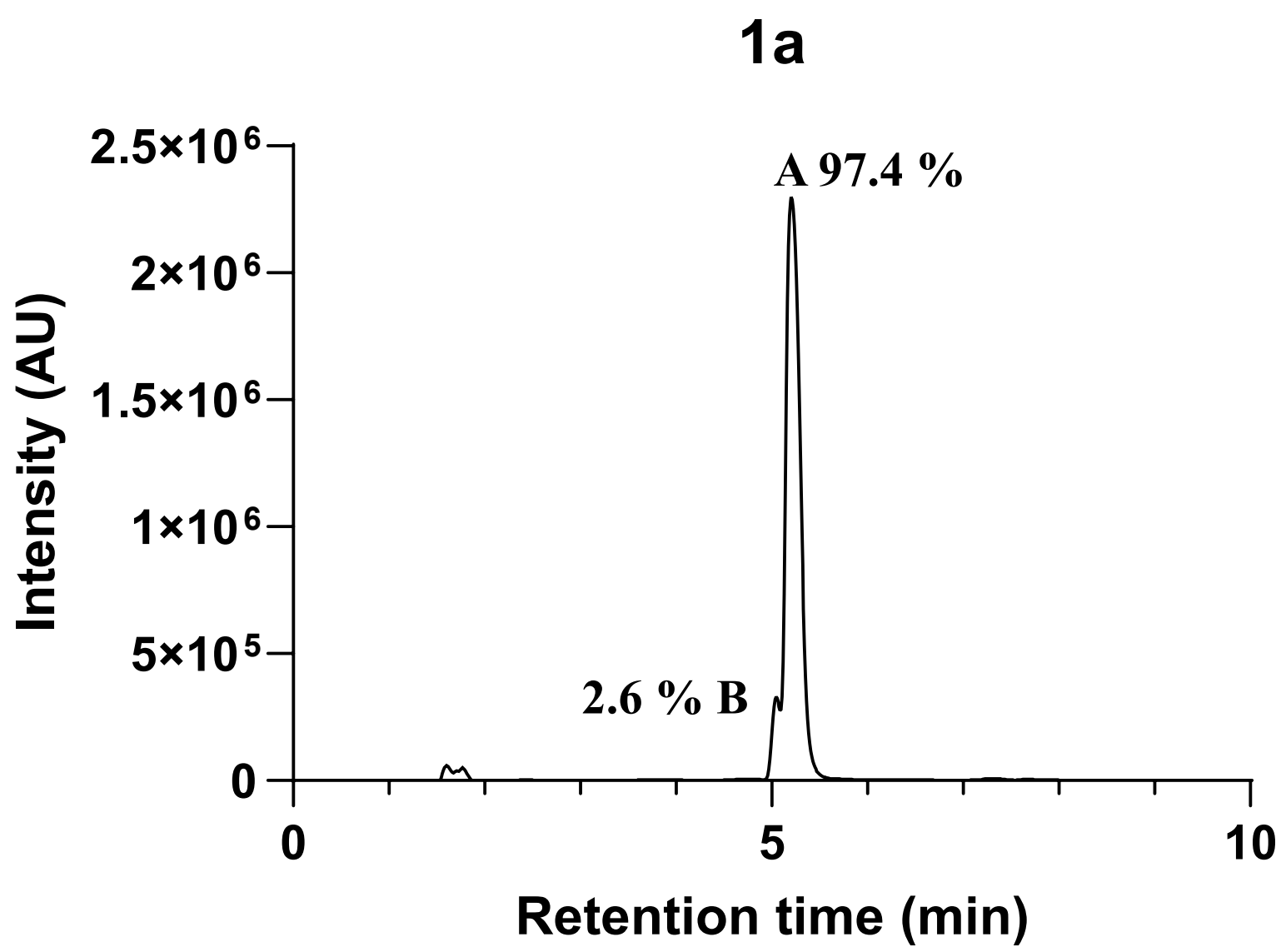

1-(3,4-Dihydroxyphenyl)-3-(4-hydroxyphenethyl)urea (1b)

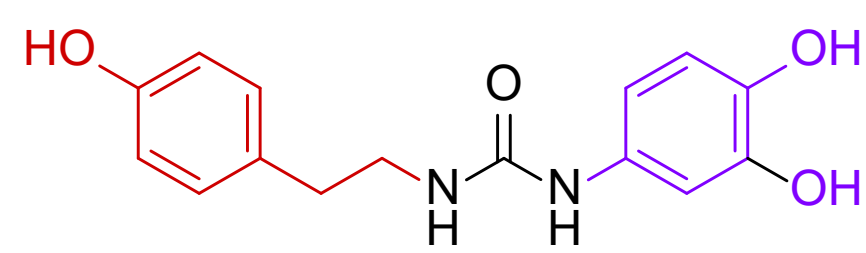

Method D was used to generate this chromatogram.

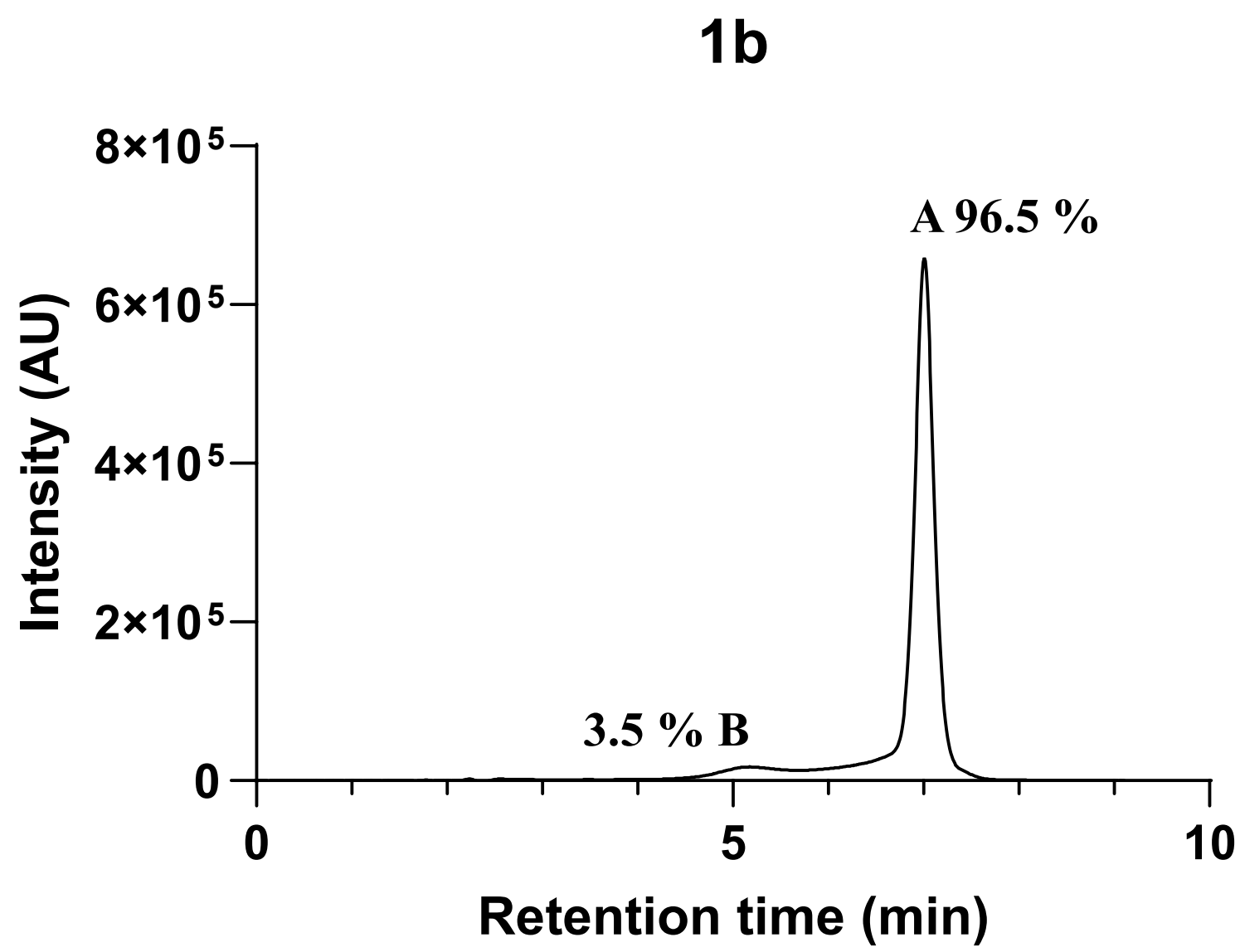

1-(3,4-Dihydroxyphenethyl)-3-(4-nitrophenyl)urea (1c)

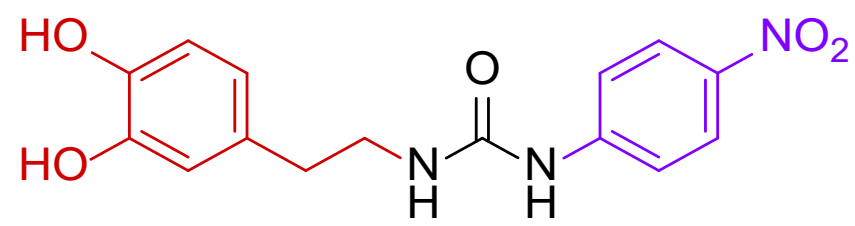

Method E was used to generate this chromatogram.

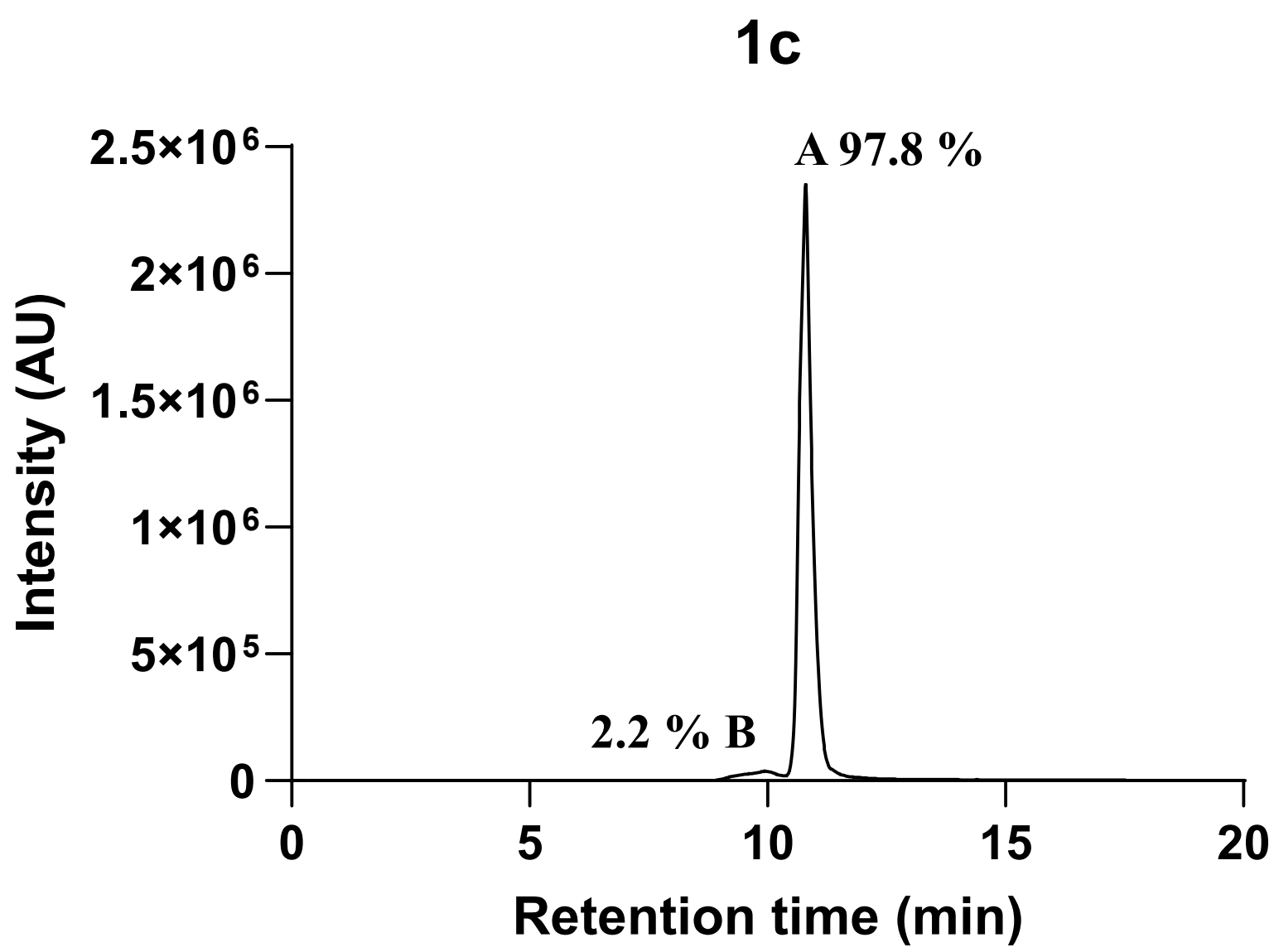

Methyl 4-[3-(3,4-dihydroxyphenethyl)ureido]benzoate (1d)

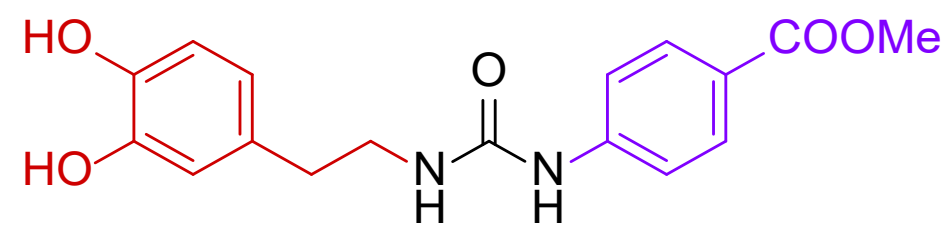

Method B was used to generate this chromatogram.

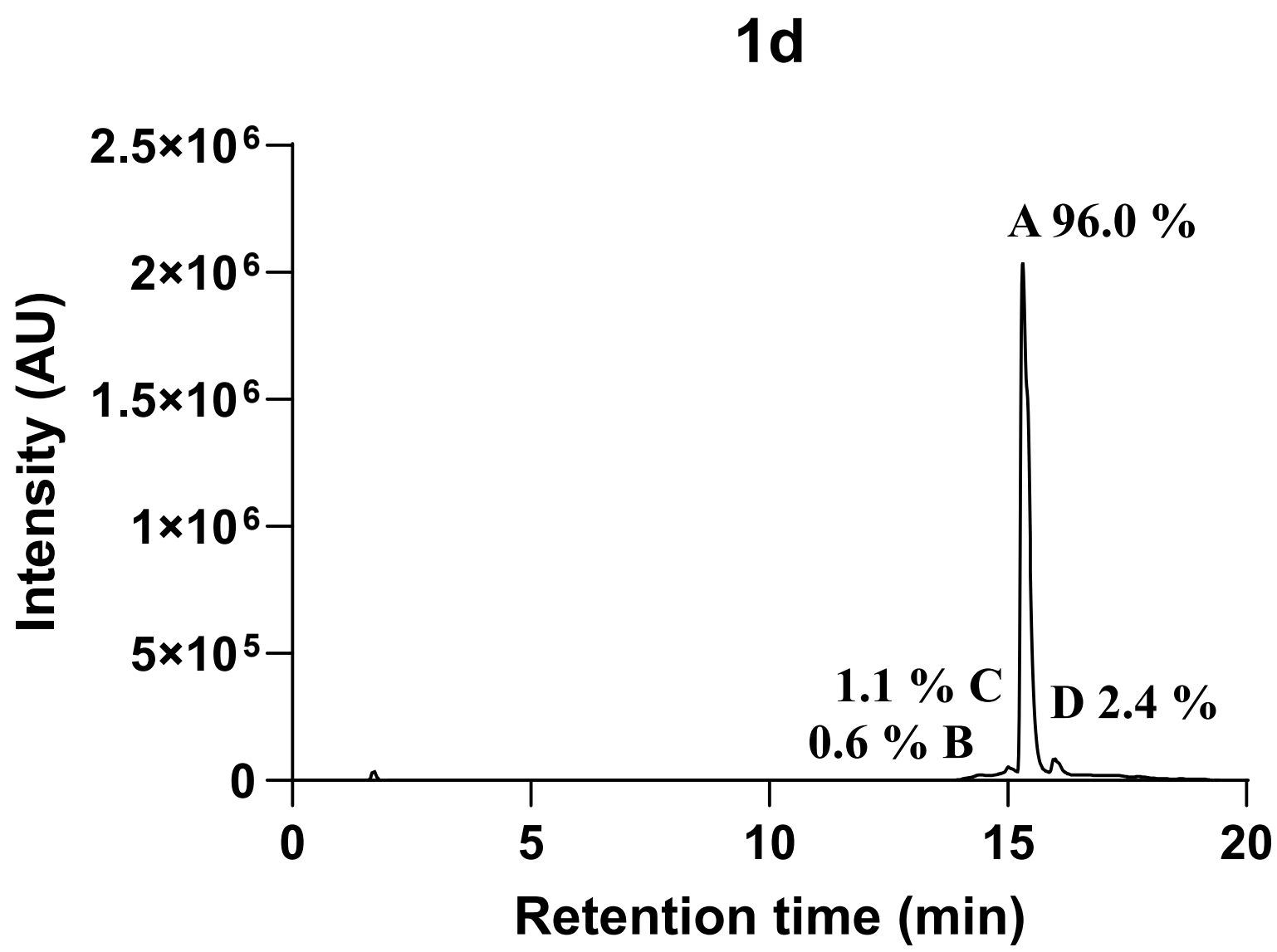

1-(4-Aminophenethyl)-3-(benzo[d][1,3]dioxol-5-yl)urea (7)

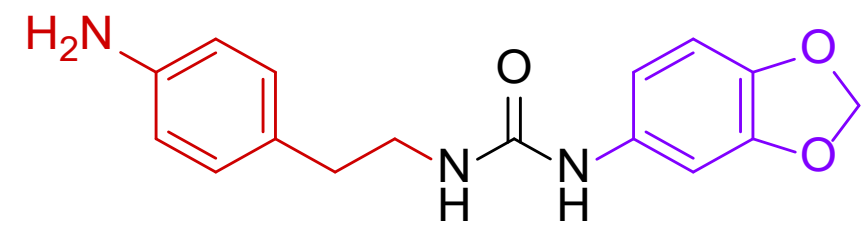

Method C was used to generate this chromatogram.

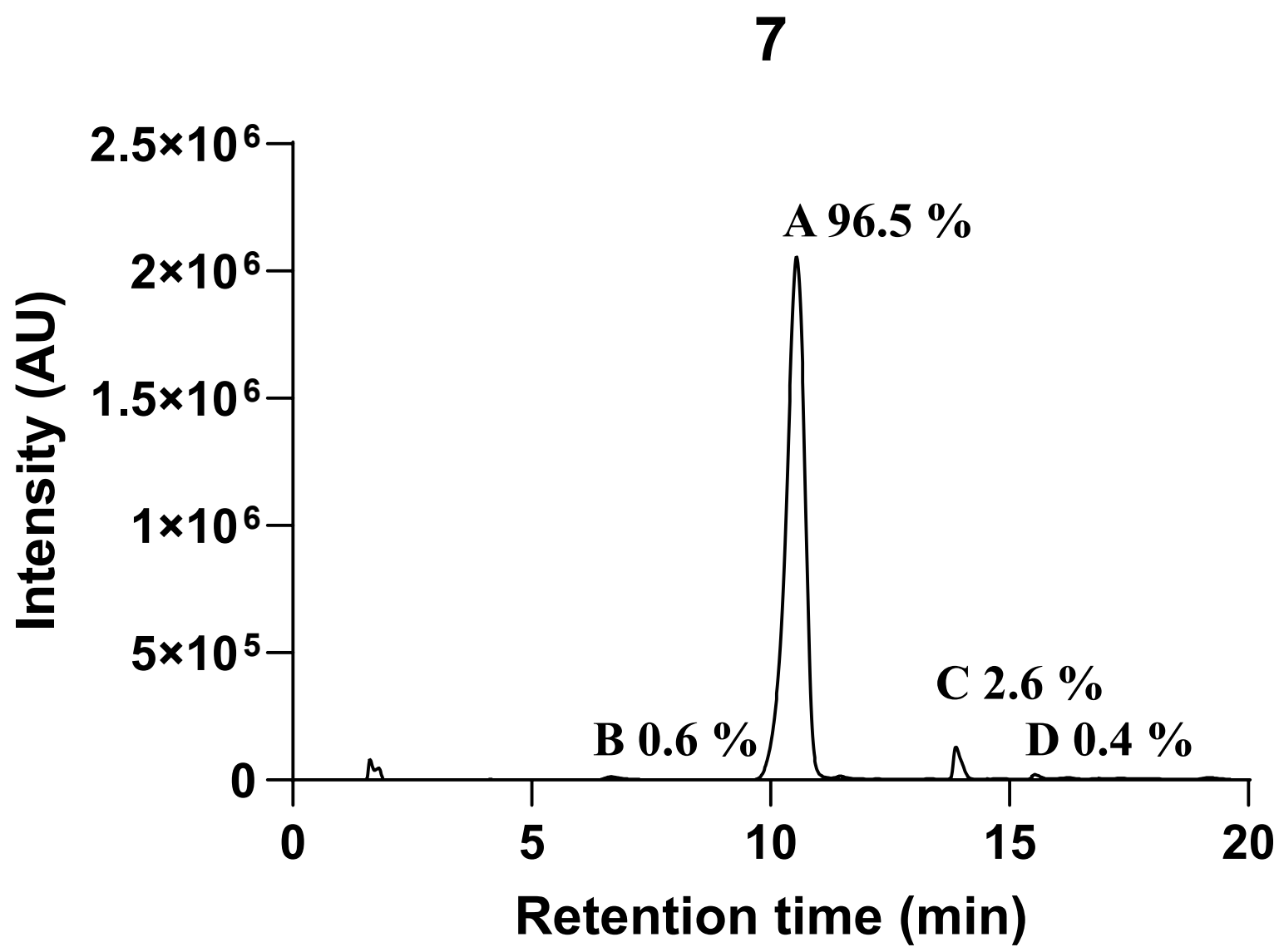

1-(4-Aminophenethyl)-3-(3,4-dihydroxyphenyl)urea (1e)

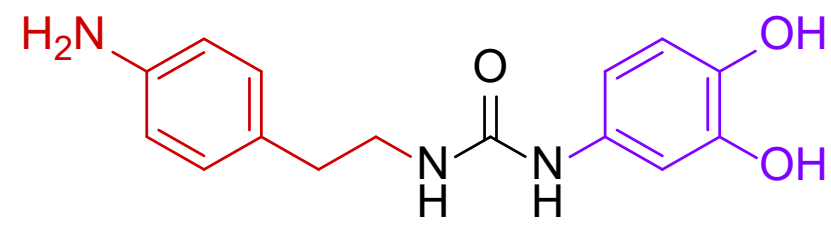

Method B was used to generate this chromatogram.

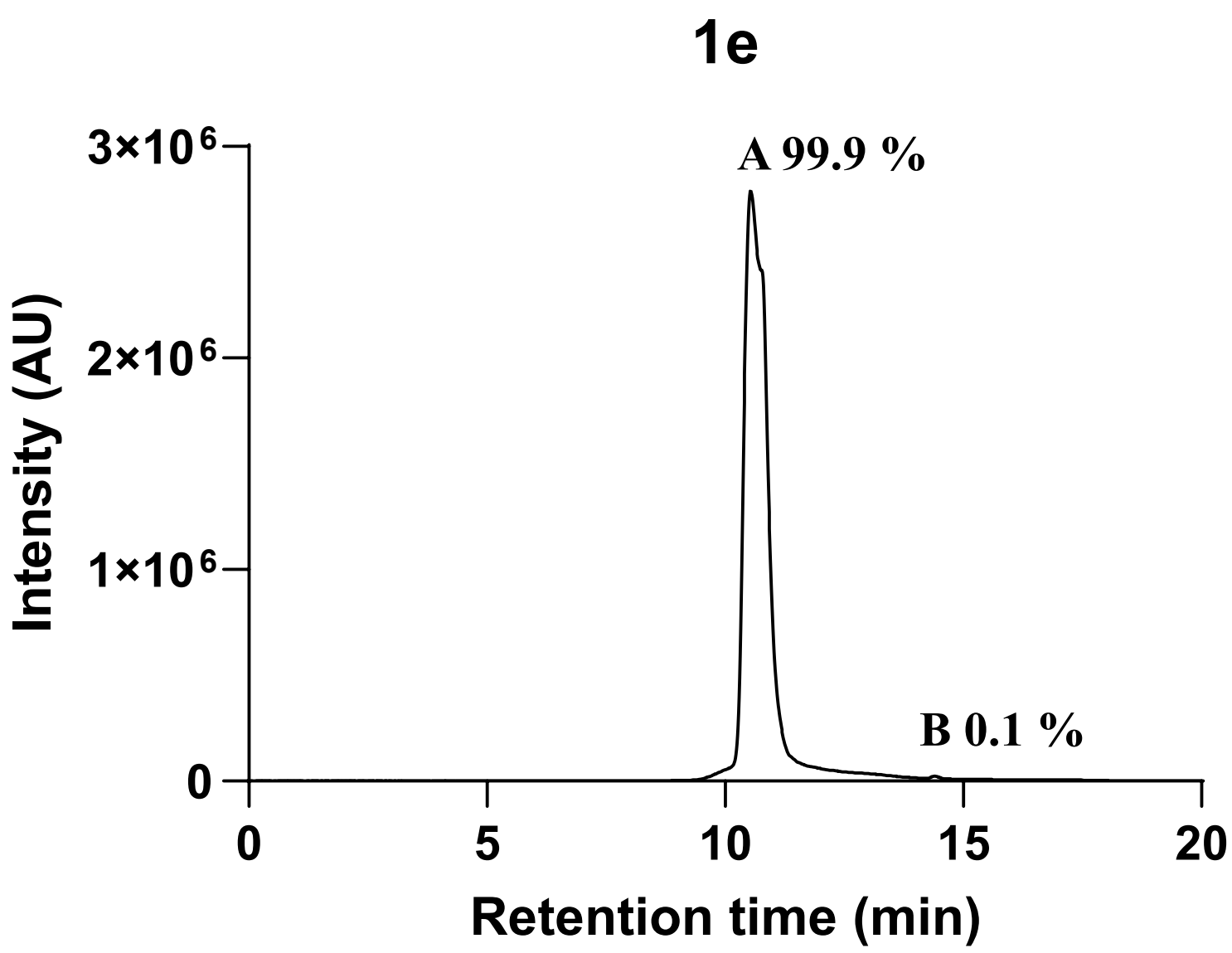

1-(4-Aminophenyl)-3-[2-(2H-1,3-benzodioxol-5-yl)ethyl]urea (8)

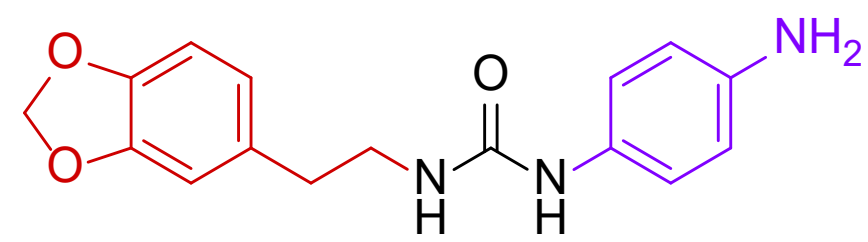

Method C was used to generate this chromatogram.

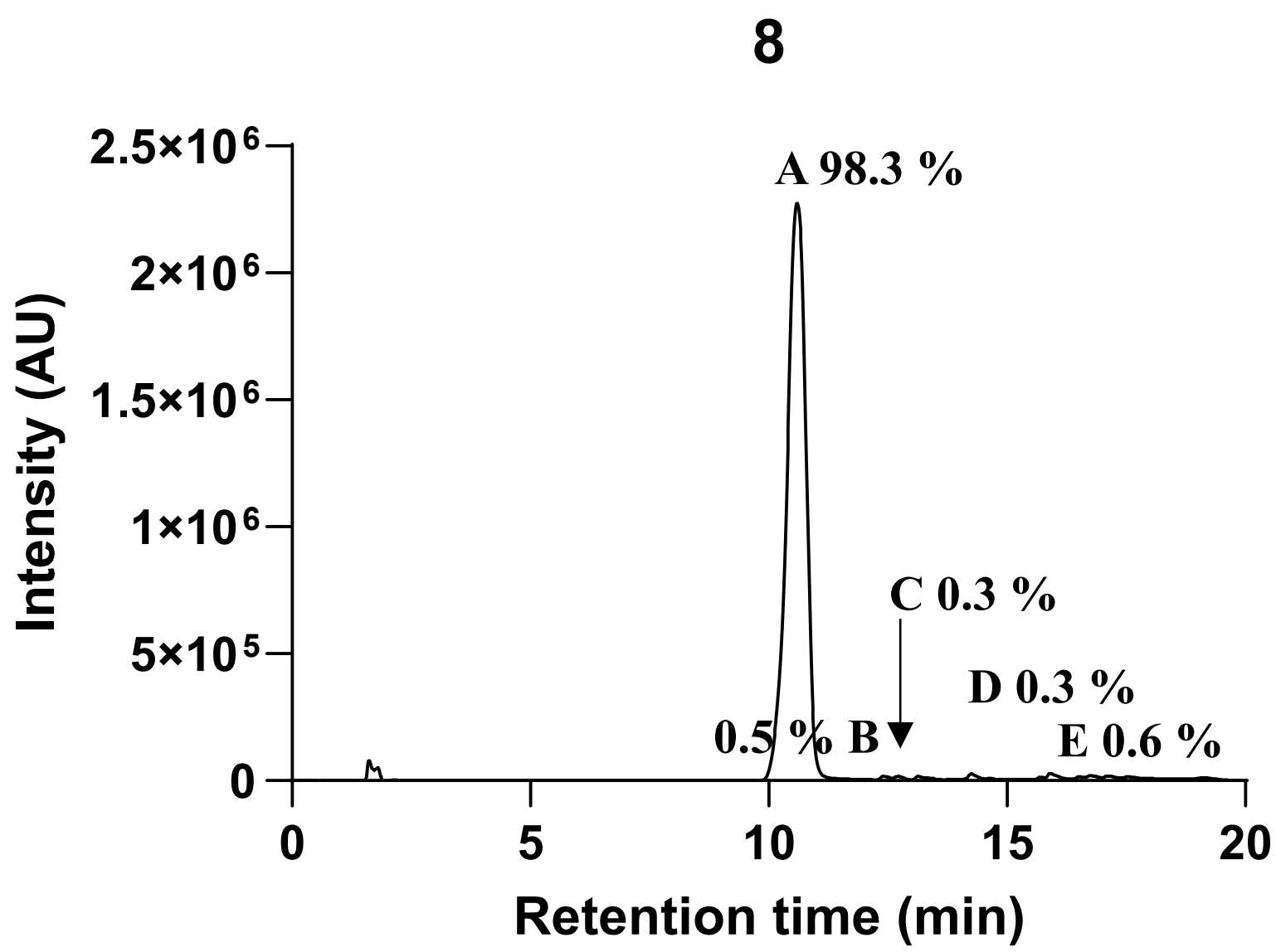

1-(4-Aminophenyl)-3-(3,4-dihydroxyphenethyl)urea (1f)

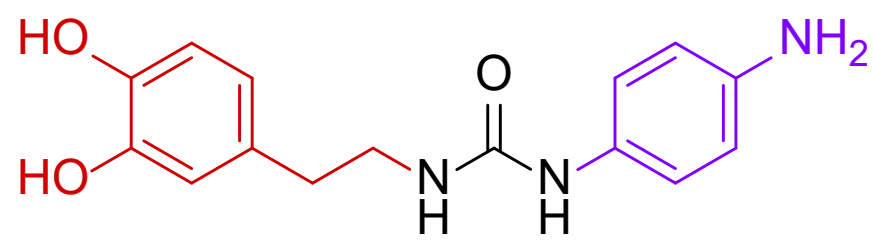

Method B with 0.1 % trifluoroacetic acid included in each mobile phase was used to generate this chromatogram.

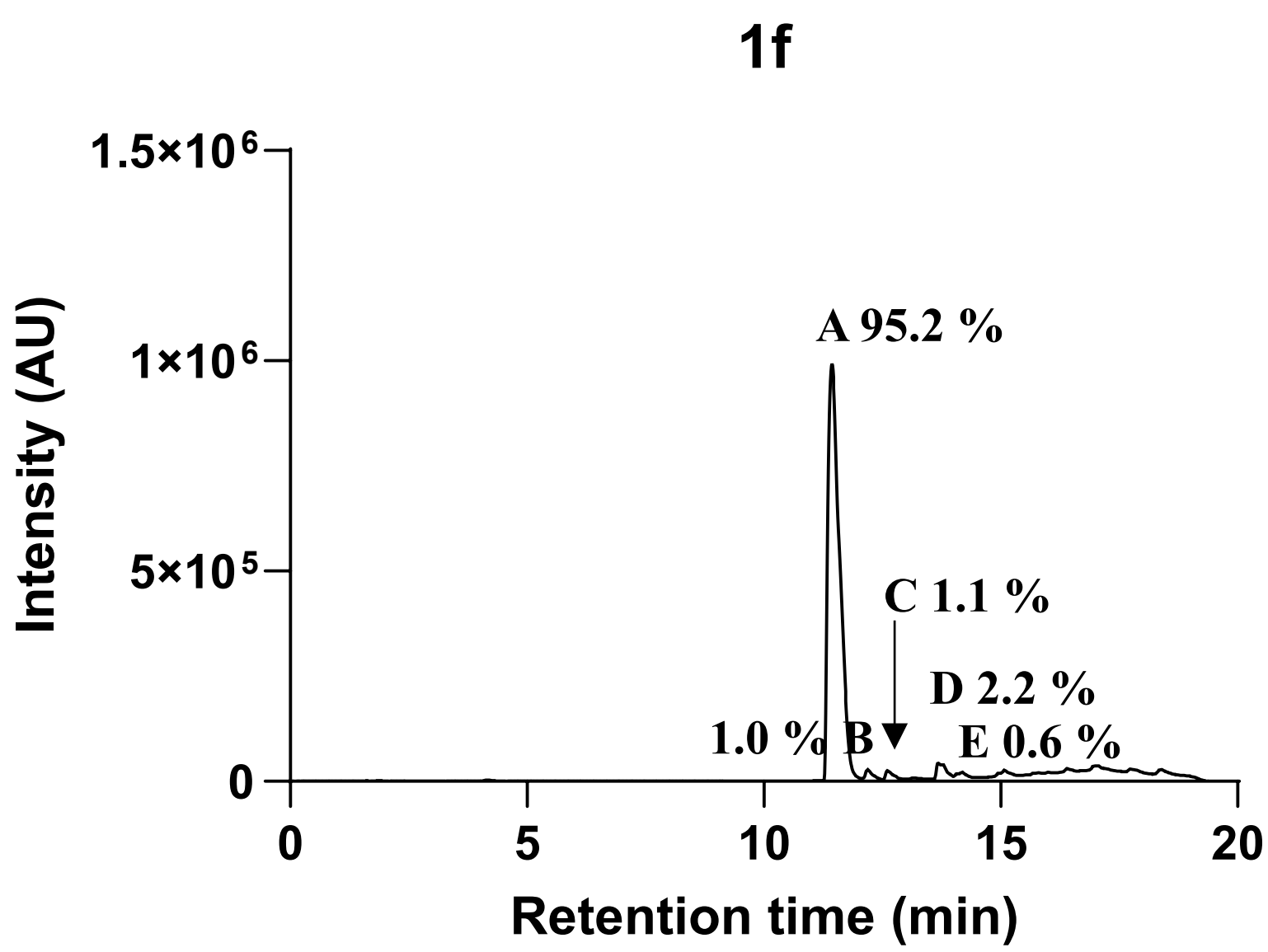

4-(3-(2-(Benzo[d][1,3]dioxol-5-yl)ethyl)ureido)benzoic acid (9)

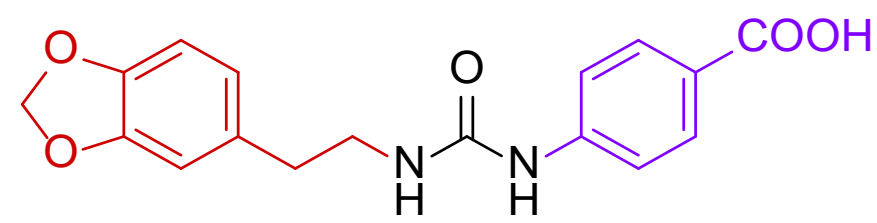

Method C was used to generate this chromatogram.

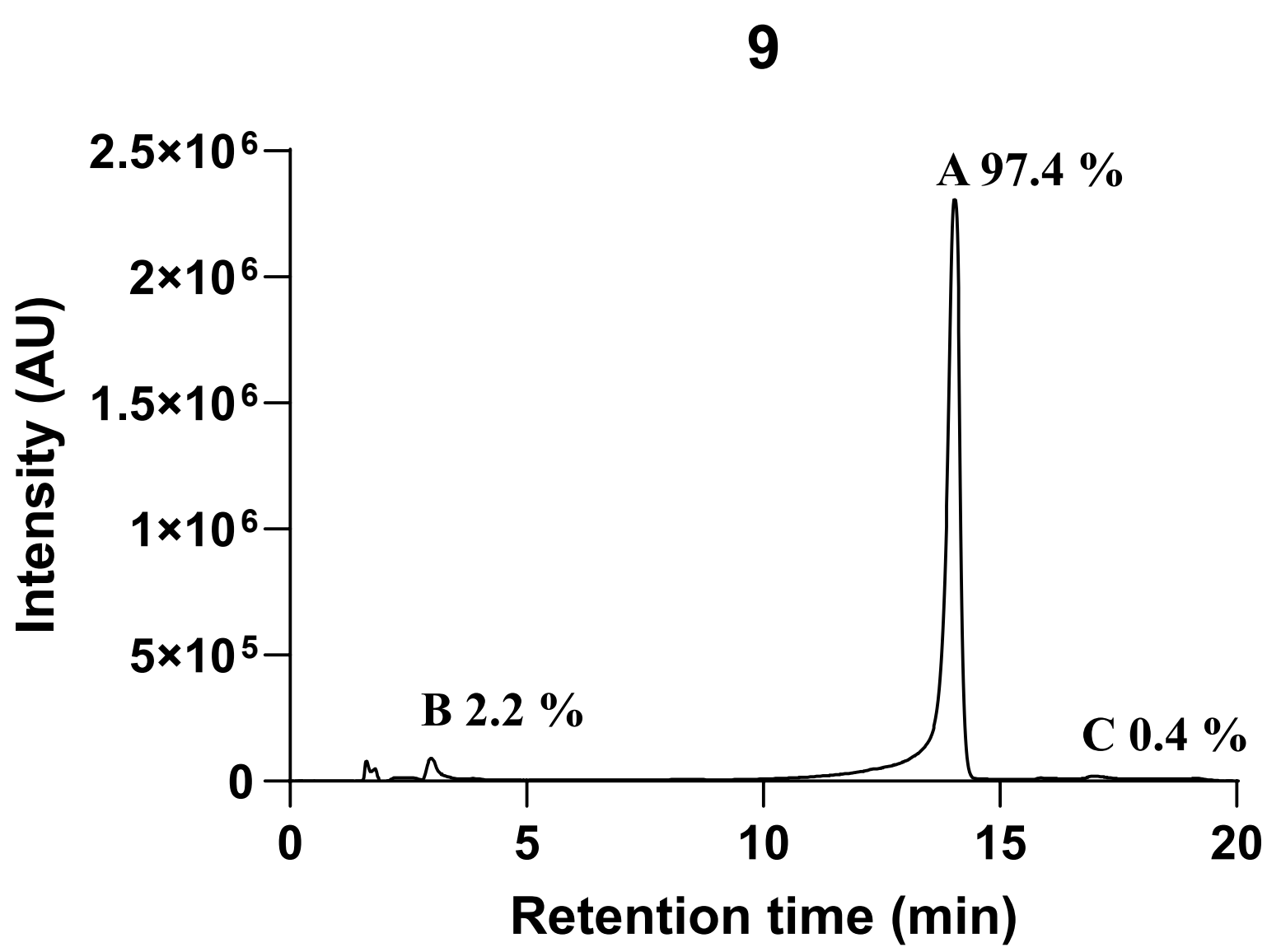

4-(3-(3,4-Dihydroxyphenethyl)ureido)benzoic acid (1g)

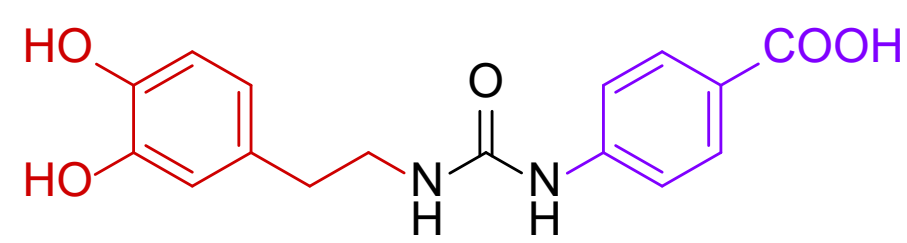

Method B was used to generate this chromatogram.

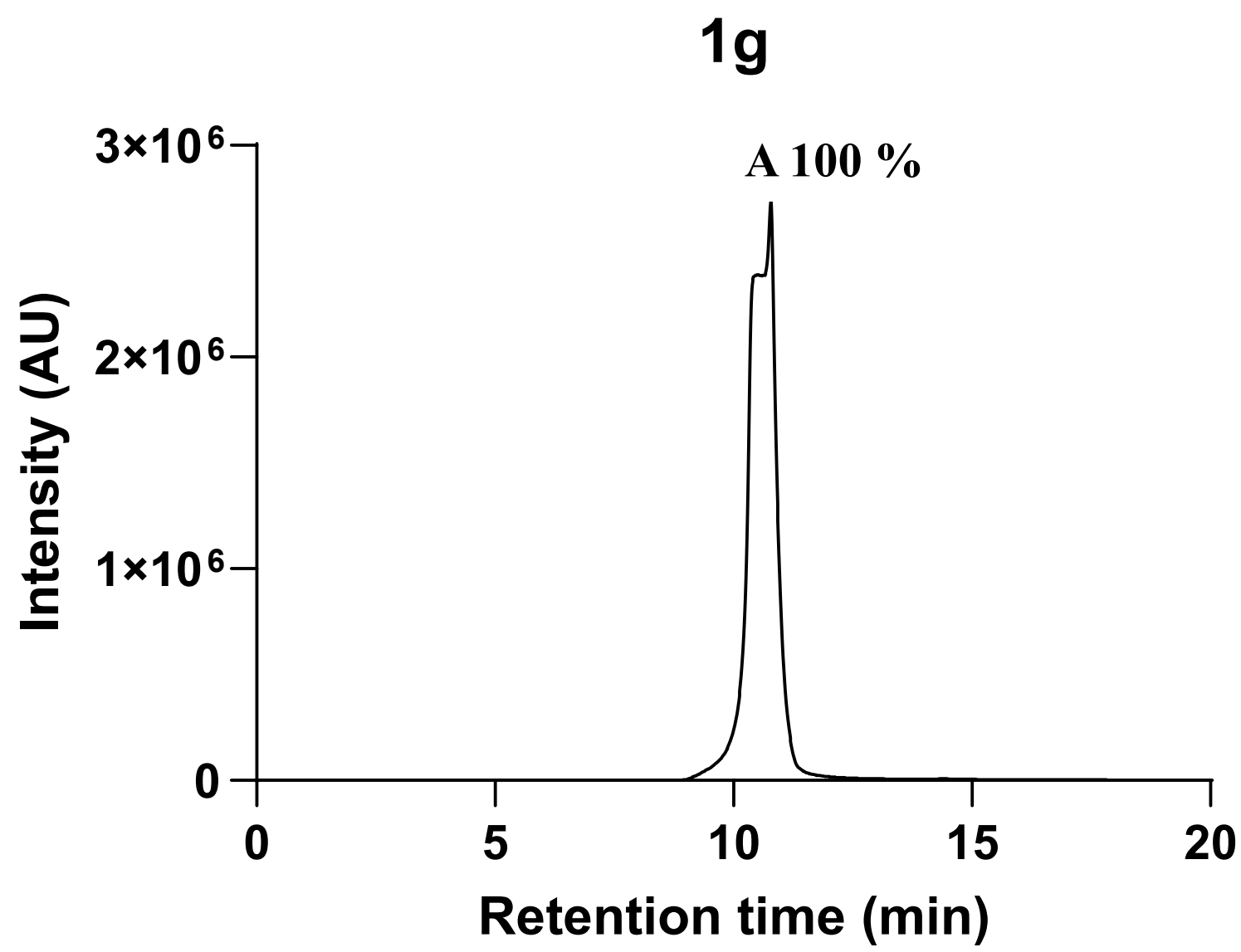

2-(Benzo[d][1,3]dioxol-5-yl)-N-(2-(benzo[d][1,3]dioxol-5-yl)ethyl)acetamide (11)

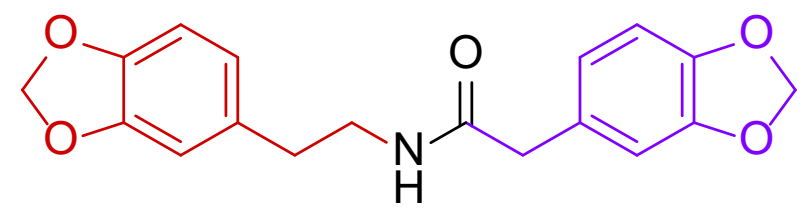

Method C was used to generate this chromatogram.

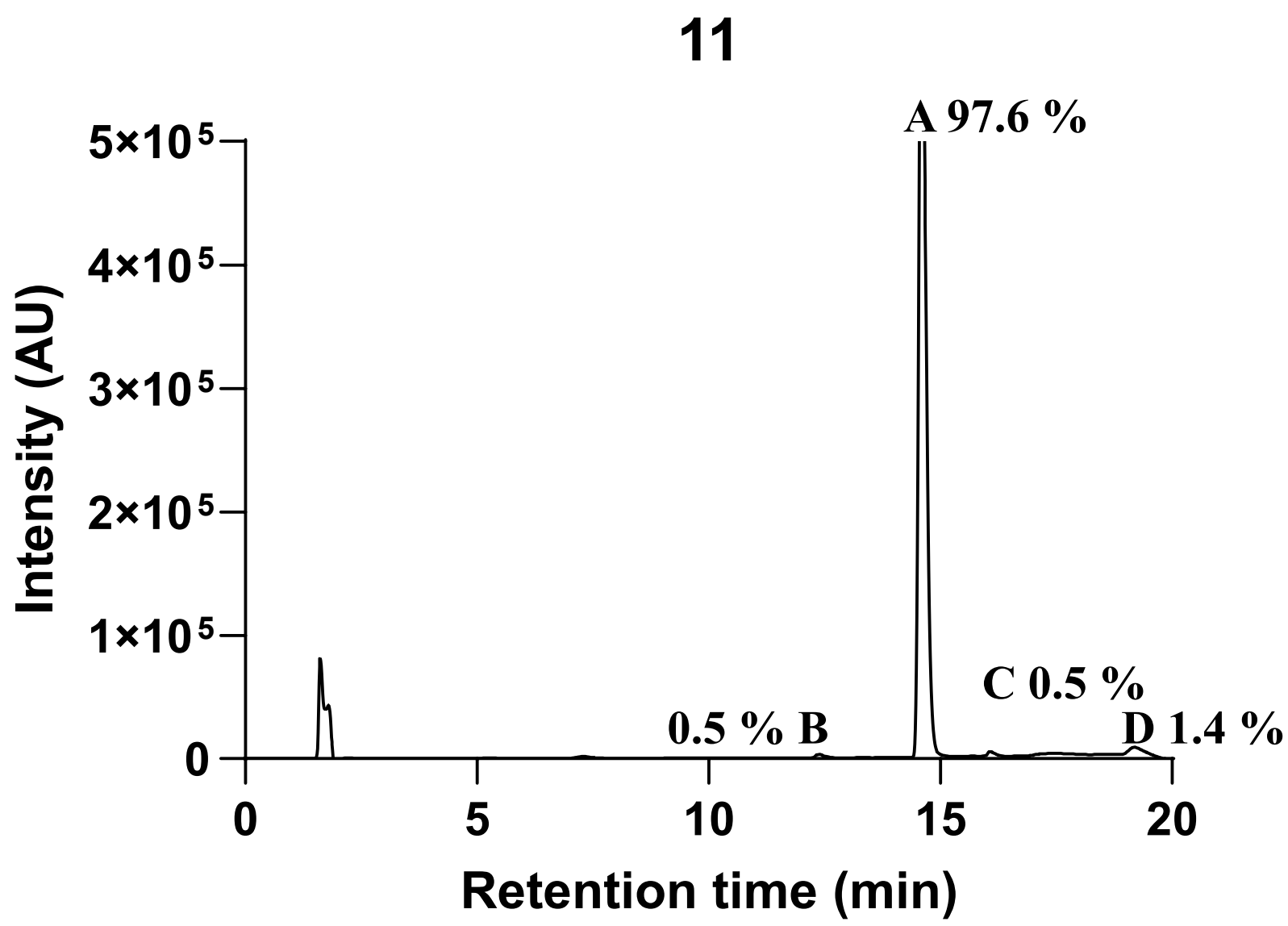

N-(3,4-Dihydroxyphenethyl)-2-(3,4-dihydroxyphenyl)acetamide (1h)

^{1}H-NMR (400 MHz, acetone-d_6)

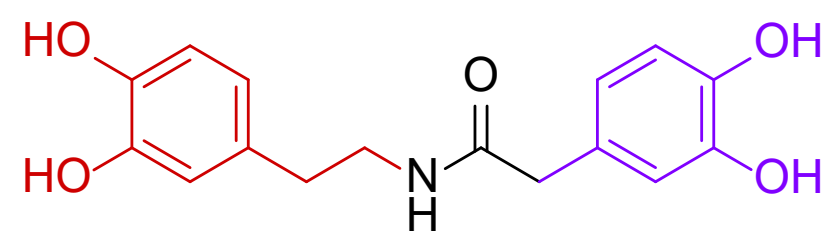

Method B was used to generate this chromatogram.

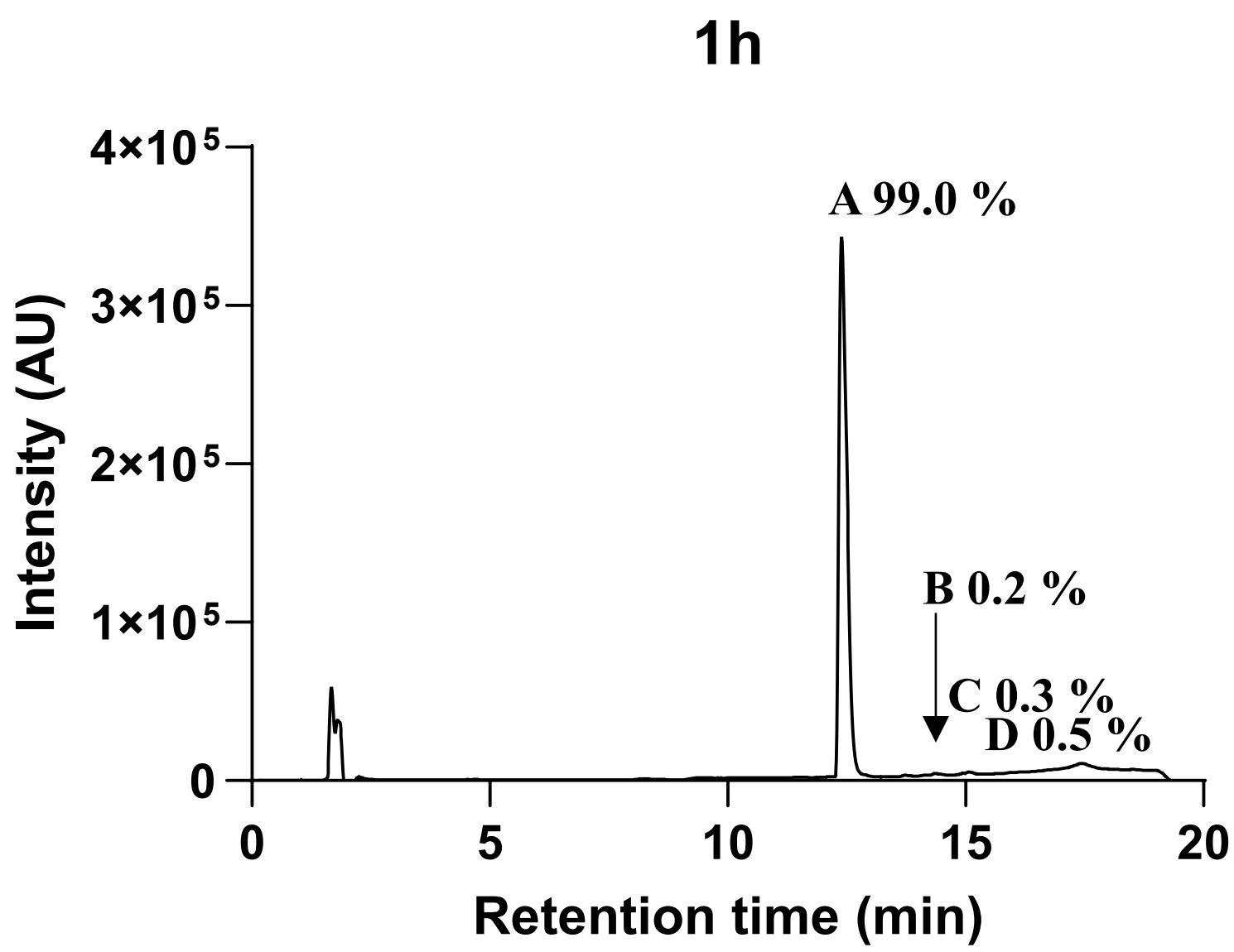

1,3-Bis(2-(benzo[d][1,3]dioxol-5-yl)ethyl)urea (13)

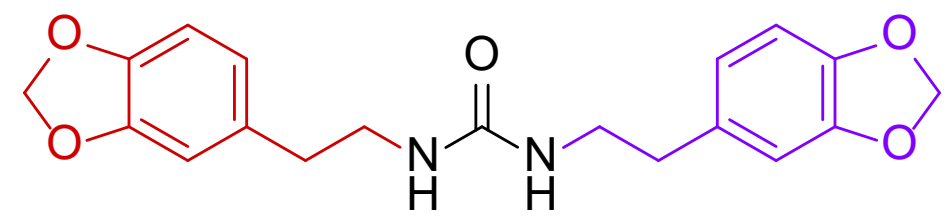

Method C was used to generate this chromatogram.

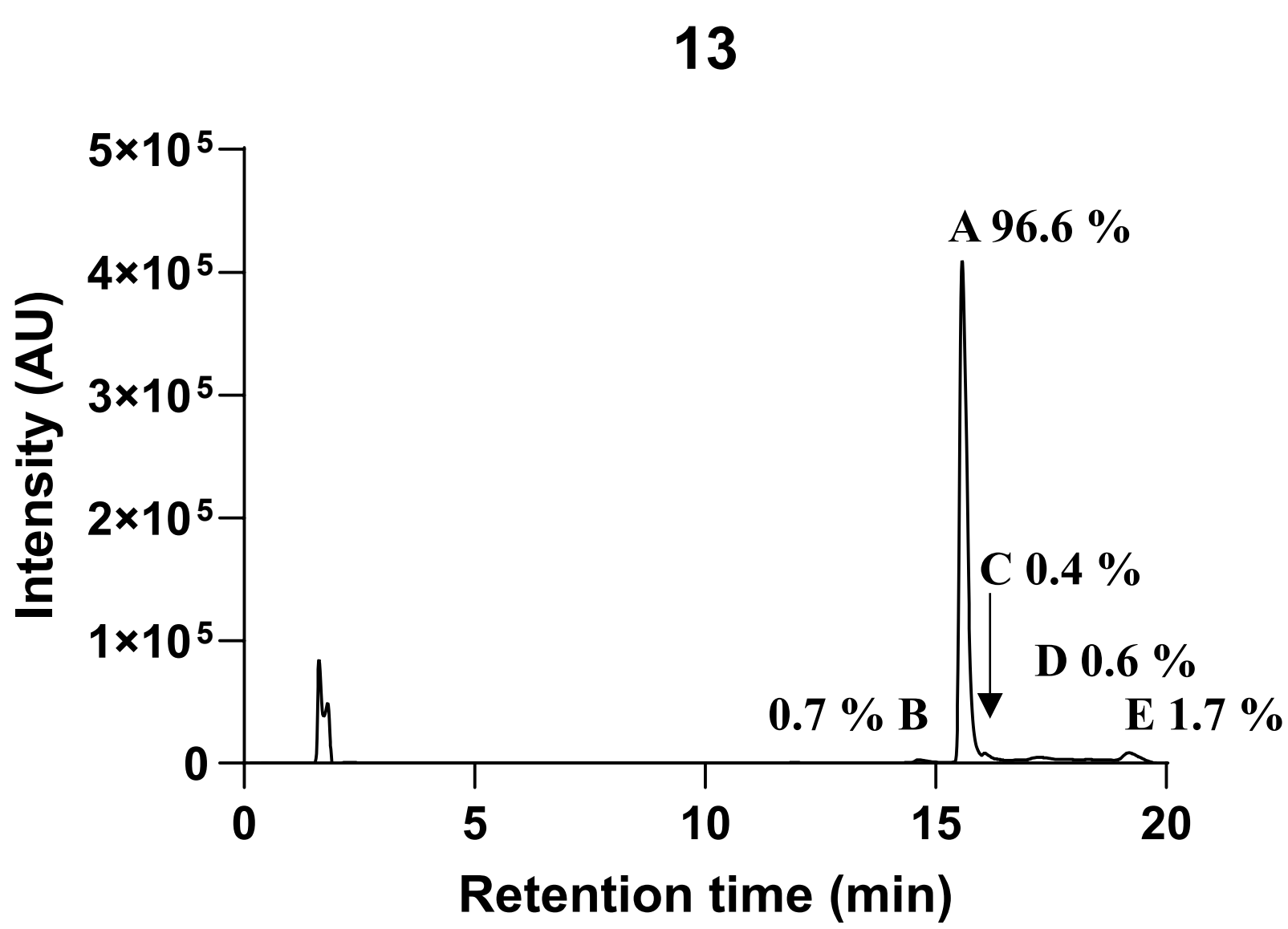

1,3-Bis(3,4-dihydroxyphenethyl)urea (1i)

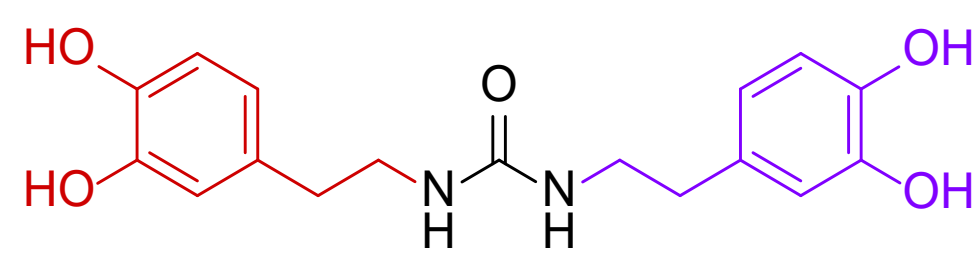

Method B was used to generate this chromatogram.

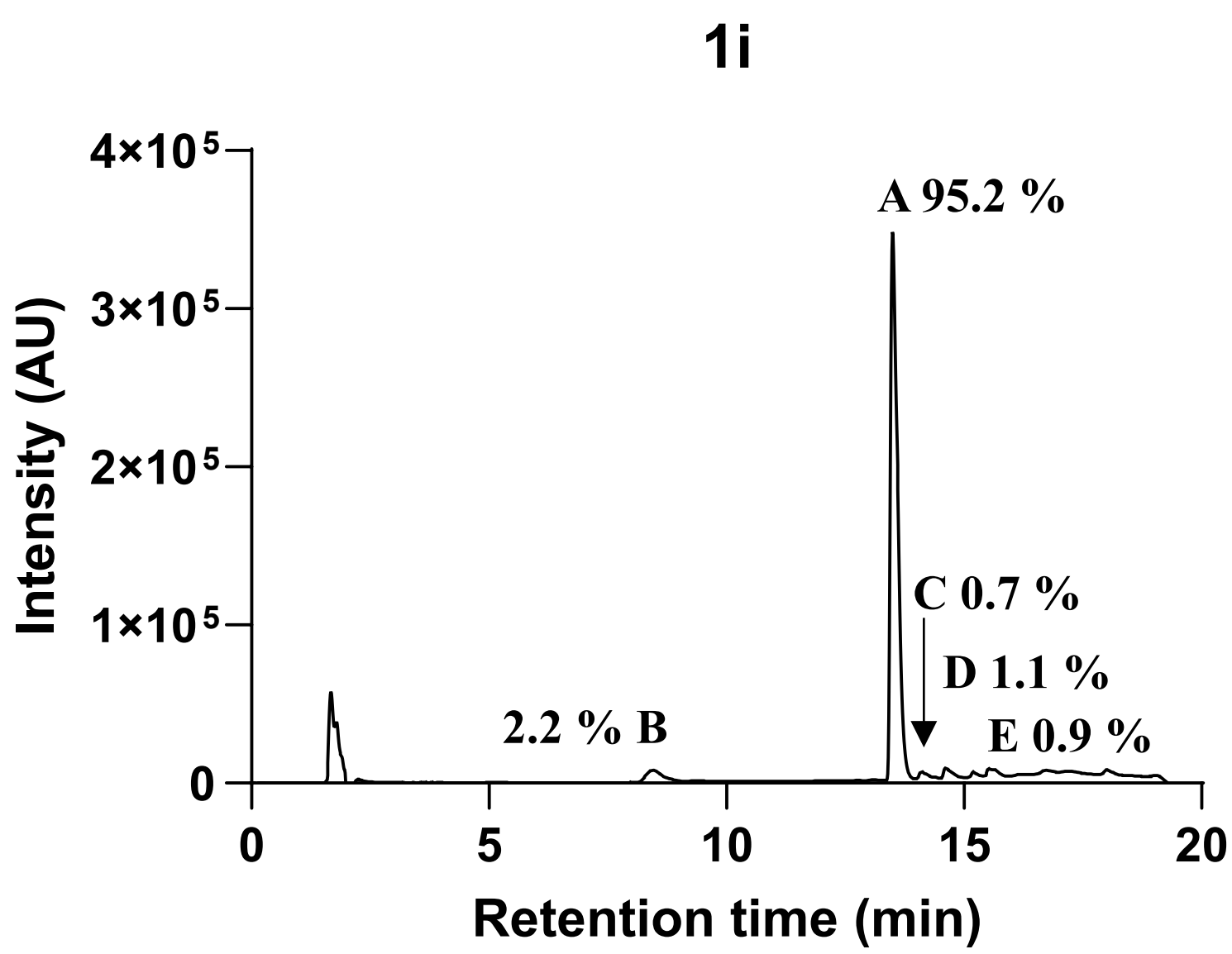

1-(Benzo[d][1,3]dioxol-5-yl)-3-(2-(benzo[d][1,3]dioxol-5-yl)ethyl)thiourea (15)

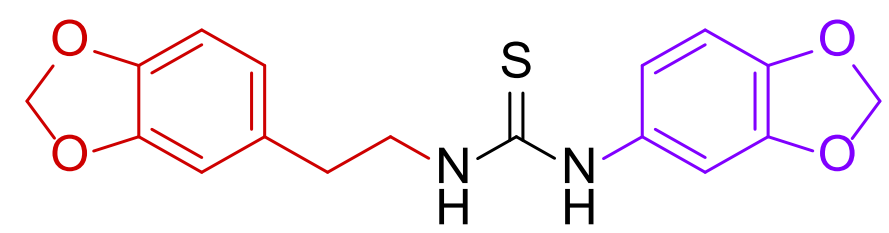

Method C was used to generate this chromatogram.

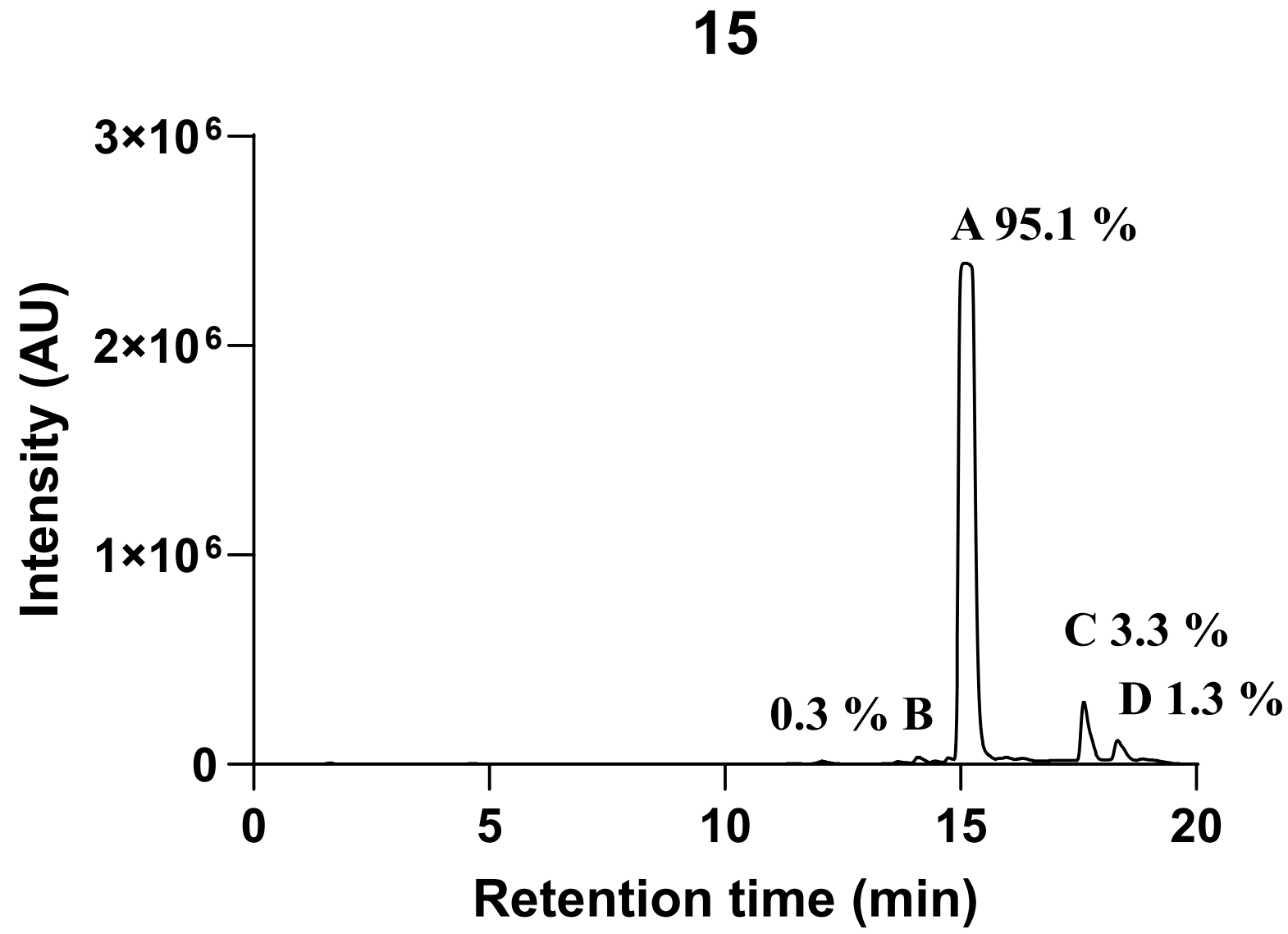

1-(3,4-Dihydroxyphenethyl)-3-(3,4-dihydroxyphenyl)thiourea (1j)

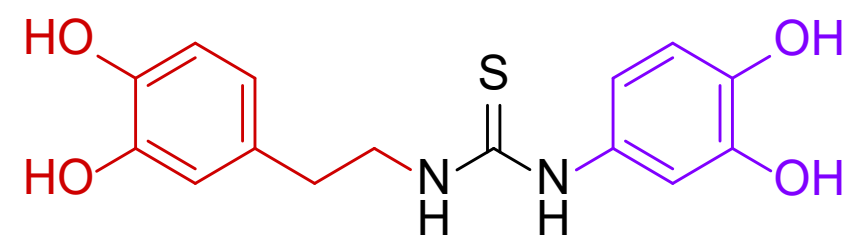

Method B was used to generate this chromatogram.

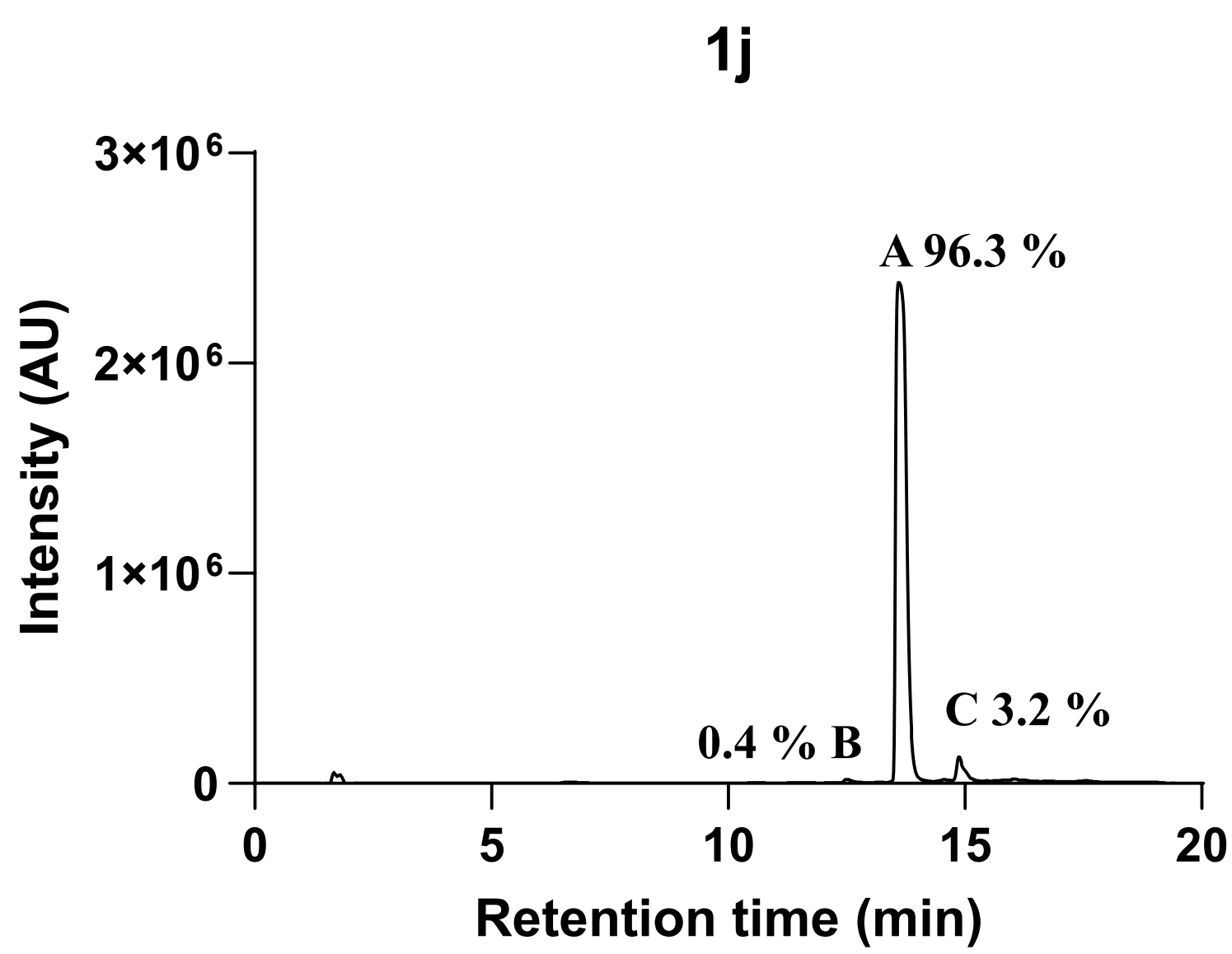

Supplement: Supplementary file 1 [file ml6c00275_si_001.pdf]
